# Vitiligo in Deutschland: Eine Abrechnungsdatenanalyse zu Prävalenz, Komorbidität und Behandlungsmustern

**DOI:** 10.1007/s00105-026-05764-5

**Published:** 2026-08-24

**Authors:** Markus Böhm, Lisa-Marie Müller, Alexa Benson, Alexander Heuck, Michael Sebastian

**Affiliations:** 1 Klinik für Hautkrankheiten, Universitätsklinikum Münster, Von-Esmarch-Str. 58, 48149 Münster, Deutschland; 2 Gesundheitsforen Leipzig GmbH, Leipzig, Deutschland; 3 AbbVie Deutschland GmbH & Co. KG, Wiesbaden, Deutschland; 4 Dermatologie Mahlow, Haut- und Studienzentrum, Blankenfelde-Mahlow, Deutschland

**Keywords:** Gesetzliche Krankenversicherung, Krankenkasse, Autoimmunerkrankung, Versorgungssituation, Unterversorgung, Statutory health insurance, Health insurance fund, Autoimmune disease, Healthcare situation, Undertreatment

## Abstract

**Hintergrund:**

Vitiligo ist eine chronische immunvermittelte Erkrankung, charakterisiert durch depigmentierte Hautareale und assoziiert mit vielfältigen psychischen und somatischen Begleiterkrankungen. Für Deutschland existieren nur begrenzt aktuelle Daten zu Prävalenz, Komorbidität und Therapieverhalten.

**Methodik:**

In dieser retrospektiven Analyse von Abrechnungsdaten der gesetzlichen Krankenversicherung (GKV) wurden Informationen von Versicherten (≥ 12 bzw. ≥ 18 Jahre) aus 2023 ausgewertet. Prävalenzfälle wurden anhand des ICD-10-GM-Codes für Vitiligo nach M2Q-Kriterien identifiziert, Inzidenzfälle durch fehlendes Auftreten im Vorjahr. Begleiterkrankungen wurden mit einer gematchten Kontrollkohorte verglichen. Die Therapieanalyse erfolgte für die Prävalenzkohorte und stratifiziert nach Fachgruppen und nach Ausschluss von Diagnosen mit überlappenden Therapien.

**Ergebnisse:**

Die GKV-adjustierte Prävalenz von Vitiligo lag bei 0,14 %, die Inzidenz bei 0,03 %, beide mit Höchstwerten in der Altersgruppe 55 bis 64 Jahre. Von 673 analysierten ICD-10-Diagnosen waren 343 (51,0 %) signifikant häufiger bei Personen mit Vitiligo als bei gematchten Kontrollen (OR: > 1; *p* ≤ 0,05), besonders Autoimmunerkrankungen wie Thyreoiditis, Alopecia areata und Diabetes mellitus Typ 1. In der Prävalenzkohorte erhielten nur 22,1 % der Betroffenen eine abgerechnete Behandlung. In der Subkohorte spezialisierter Fachgruppen und Diagnosen ohne überlappende Therapien wurden 8,2 % behandelt, davon rund 90 % mit topischen Therapien. Systemische Glukokortikoide und UV-Therapien waren selten.

**Schlussfolgerung:**

Die Analyse zeigt die aktuelle Versorgungssituation von Vitiligo in Deutschland, unterstreicht den systemischen Charakter der Erkrankung und deutet auf eine alarmierende Unterversorgung hin.

**Graphic abstract:**

**Figure:**
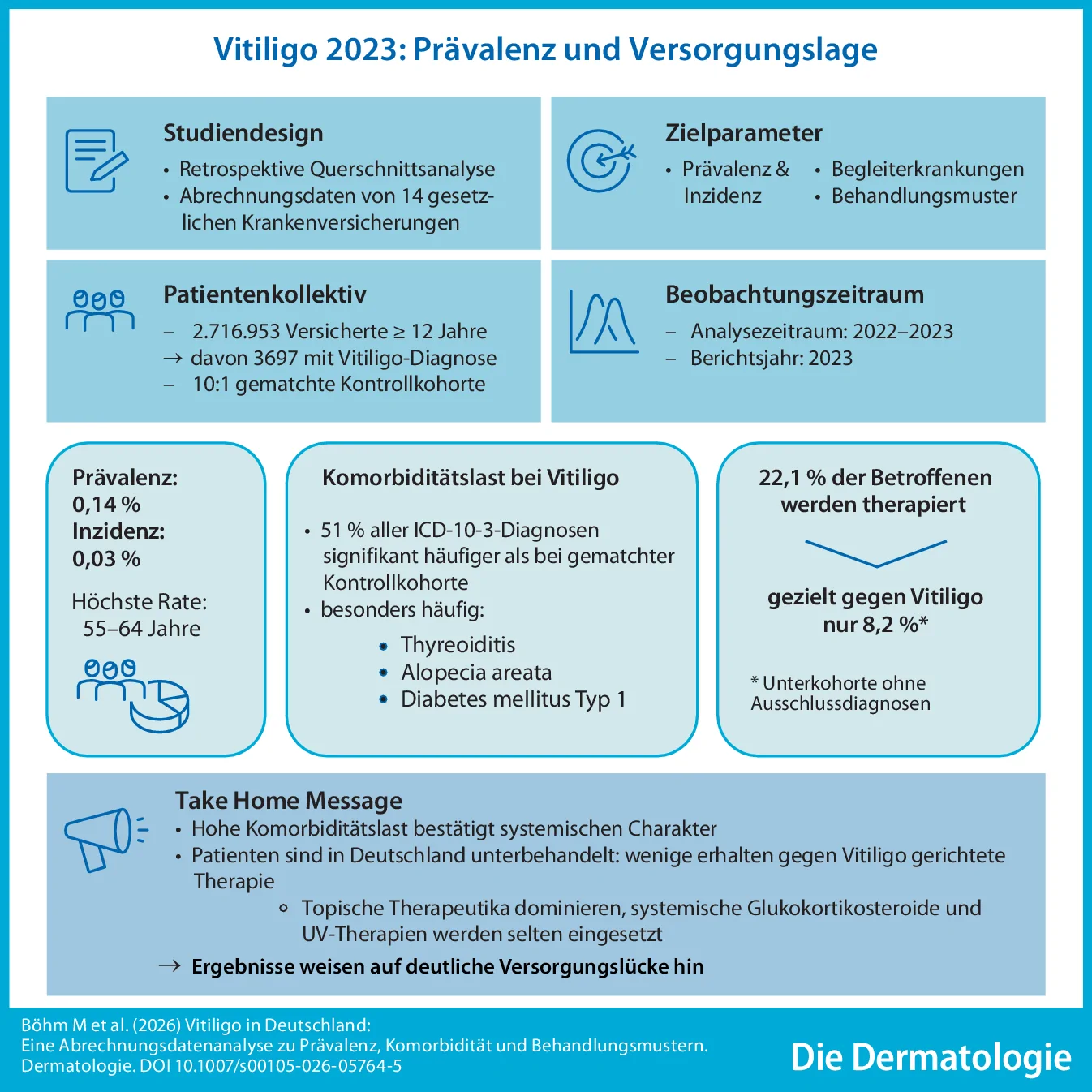

**Zusatzmaterial online:**

Die Online-Version dieses Beitrags (https://doi.org/10.1007/s00105-026-05764-5) enthält 9 zusätzliche Tabellen. Beitrag und Zusatzmaterial stehen Ihnen im elektronischen Volltextarchiv auf https://www.springermedizin.de/die-dermatologie zur Verfügung. Sie finden das Zusatzmaterial am Beitragsende.

## Abstract (en)

**Background:**

Vitiligo is a chronic immune-mediated disease characterized by depigmented skin areas and associated with a wide range of psychological and physical comorbidities. Data on prevalence, comorbidity, and treatment patterns in Germany are currently limited.

**Methods:**

In this retrospective analysis of statutory health insurance (SHI) claims data, information from insured individuals (≥ 12 and ≥ 18 years) from 2023 was evaluated. Prevalent cases were identified using the ICD-10-GM code for vitiligo according to M2Q criteria; incident cases were defined by the absence of diagnosis in the previous year. Comorbidities were compared with a matched control cohort. The therapy analysis was conducted for the prevalence cohort and stratified by medical specialty as well as by exclusion of diagnoses with overlapping therapies.

**Results:**

The SHI-adjusted prevalence of vitiligo was 0.14%, with an incidence of 0.03%, both peaking in the 55–64 age group. Of 673 examined ICD-10 diagnoses, 343 (51.0%) occurred significantly more often in vitiligo patients compared to matched controls (odds ratio > 1; *p* ≤ 0.05), particularly autoimmune diseases such as thyroiditis, alopecia areata, and type 1 diabetes mellitus. In the prevalence cohort, only 22.1% of those affected received any reimbursed treatment. In the subcohort of specialized medical groups and diagnoses without overlapping treatment patterns, 8.2% were treated, of whom around 90% received topical therapy. Systemic glucocorticoids and UV therapies were rarely prescribed.

**Conclusion:**

The analysis illustrates the current care situation for vitiligo in Germany, emphasizes the systemic nature of the disease, and suggests an alarming undertreatment.

Vitiligo ist eine chronische Erkrankung, die durch charakteristische Pigmentstörungen der Haut gekennzeichnet ist [13]. Die globale Prävalenz beträgt etwa 0,5–2 % mit teils deutlichen regionalen Unterschieden [2, 18]. Für Deutschland gibt es nur wenige aktuelle Daten; frühere Studien zeigen Werte zwischen 0,17 % und 0,77 % (2004–2014; in der Altersgruppe 16 bis 70 Jahre) [23]. In der vorliegenden retrospektiven Analyse wurden Krankenkassendaten aus 2023 zu Prävalenz, Inzidenz, Demografie, Begleiterkrankungen und Behandlungsmustern von Personen mit Vitiligo in Deutschland analysiert.

Vitiligo ist eine chronische Hauterkrankung mit depigmentierten Hautarealen unterschiedlicher Größe und Lokalisation [6]. Besonders die nichtsegmentale Vitiligo (NSV) beeinträchtigt den Alltag, verursacht erheblichen psychosozialen Leidensdruck und mindert die Lebensqualität [3]. Betroffene leiden oft an psychischen Komorbiditäten wie Angststörungen und Depressionen sowie somatischen Begleiterkrankungen, meist aus dem autoimmunen und chronisch entzündlichen Formenkreis [7, 8, 10, 19, 20]. Die NSV gilt als Autoimmunerkrankung mit systemischem Charakter.

Vitiligo wird meist unspezifisch immunsuppressiv oder immunmodulatorisch behandelt. Topische Glukokortikosteroide und Calcineurininhibitoren dienen der Entzündungshemmung und Krankheitsstabilisierung [5]. Bei ausgedehnten Verläufen kommen zusätzlich systemische Glukokortikosteroide oder physikalische Verfahren hinzu [5]. Diese Maßnahmen können eine partielle Repigmentierung induzieren, sind jedoch hinsichtlich Ansprechrate, Dauerhaftigkeit und Nebenwirkungsprofil limitiert [25]. Neue Therapieansätze sind erforderlich, die gezielt gegen immunologische Signalwege wirken [21]. Seit 2023 ist der JAK1/2-Inhibitor Ruxolitinib als topische, zielgerichtete Therapie in Europa verfügbar [9]. Diese Studie analysiert Prävalenz, Inzidenz, demografische Merkmale, Begleiterkrankungen und Behandlungsmuster von Personen mit Vitiligo anhand deutscher Krankenkassendaten aus 2023. Der klinische Stellenwert von topischem Ruxolitinib ist noch unklar.

## Methodik

### Design

Die retrospektive Querschnittsanalyse basiert auf anonymisierten Abrechnungsdaten der gesetzlichen Krankenversicherung (GKV) aus der Deutschen Analysedatenbank für Evaluation und Versorgungsforschung (DADB) der Gesundheitsforen Leipzig [17]. Sie umfasst Routinedaten von 14 gesetzlichen Krankenkassen im Analysezeitraum von Januar 2022 bis Dezember 2023 mit 2023 als Berichts- und 2022 als Vorbeobachtungsjahr.

### Kohorten

In die Prävalenzkohorte wurden Personen ≥ 12 Jahre eingeschlossen, die 2023 durchgehend versichert waren (Lücken ≤ 30 Tage) und eine Vitiligo-Diagnose (ICD-10-GM L80) nach M2Q-Kriterien aufwiesen (≥ 1 stationäre oder ≥ 2 ambulante Diagnosen in 2 Quartalen 2023). Das Indexdatum war die erste qualifizierte Diagnose 2023. Die Inzidenzkohorte erfüllte diese Kriterien und war zusätzlich 2022 voll beobachtbar, jedoch ohne Vitiligo-Diagnose. Die Kontrollkohorte umfasste Personen ohne Vitiligo-Diagnose 2022 bis 2023, ≥ 12 Jahre alt und 2023 durchgehend versichert. Für die Therapiekohorte wurde die Prävalenzkohorte nach spezialisierten Fachgruppen (Dermatologie, Allgemeinmedizin, Pädiatrie) und Ausschlussdiagnosen stratifiziert, bei denen sich die angewandten Therapien für Vitiligo (Online-Tab. 1–4) erheblich mit den Behandlungsoptionen anderer Erkrankungen überschneiden. Ausschlussdiagnosen waren Ekzeme, rheumatische Erkrankungen, chronisch entzündliche Dermatosen (u. a. auch Psoriasis), Lichen sclerosus et atrophicus und Morphea. Von allen Kohorten ausgeschlossen wurden Personen < 12 Jahren sowie während des Beobachtungszeitraums Verstorbene.

### Ziele

Das Hauptziel war die Bestimmung der Punktprävalenz von Vitiligo in Deutschland bei Personen ≥ 12 und ≥ 18 Jahren zum 31.12.2023. Weiterhin wurde die Inzidenzrate für 2023 berechnet, und Personen mit Vitiligo wurden nach Alter, Geschlecht und Komorbiditätslast beschrieben. Für die Komorbiditätsanalysen wurden Personen aus der Kontrollkohorte im Verhältnis 10:1 nach Alter, Geschlecht und Region mit der Prävalenzkohorte gematcht. Diagnosen wurden auf dreistelliger ICD-10-GM-Ebene ausgewertet.

Die Therapieauswertung umfasste topische Behandlungen, systemische Glukokortikosteroide und UV-Phototherapie.

### Statistische Analysen

Es wurden ausschließlich deskriptive Analysen durchgeführt. Prävalenz und Inzidenz wurden pro 100.000 Personen berechnet und auf die GKV-Gesamtbevölkerung in Deutschland nach Alter und Geschlecht hochgerechnet. Ergebnisse wurden als Personenanzahl, Häufigkeiten, Odds Ratios (OR) und 95 %-Konfidenzintervalle (KI) berichtet. Komorbiditäten wurden mit konditionaler logistischer Regression analysiert. Die Signifikanzprüfung erfolgte mittels Wald-Test unter Angabe der *p*-Werte.

## Ergebnisse

### Prävalenz und Inzidenz von Vitiligo

**Abb. 1:**
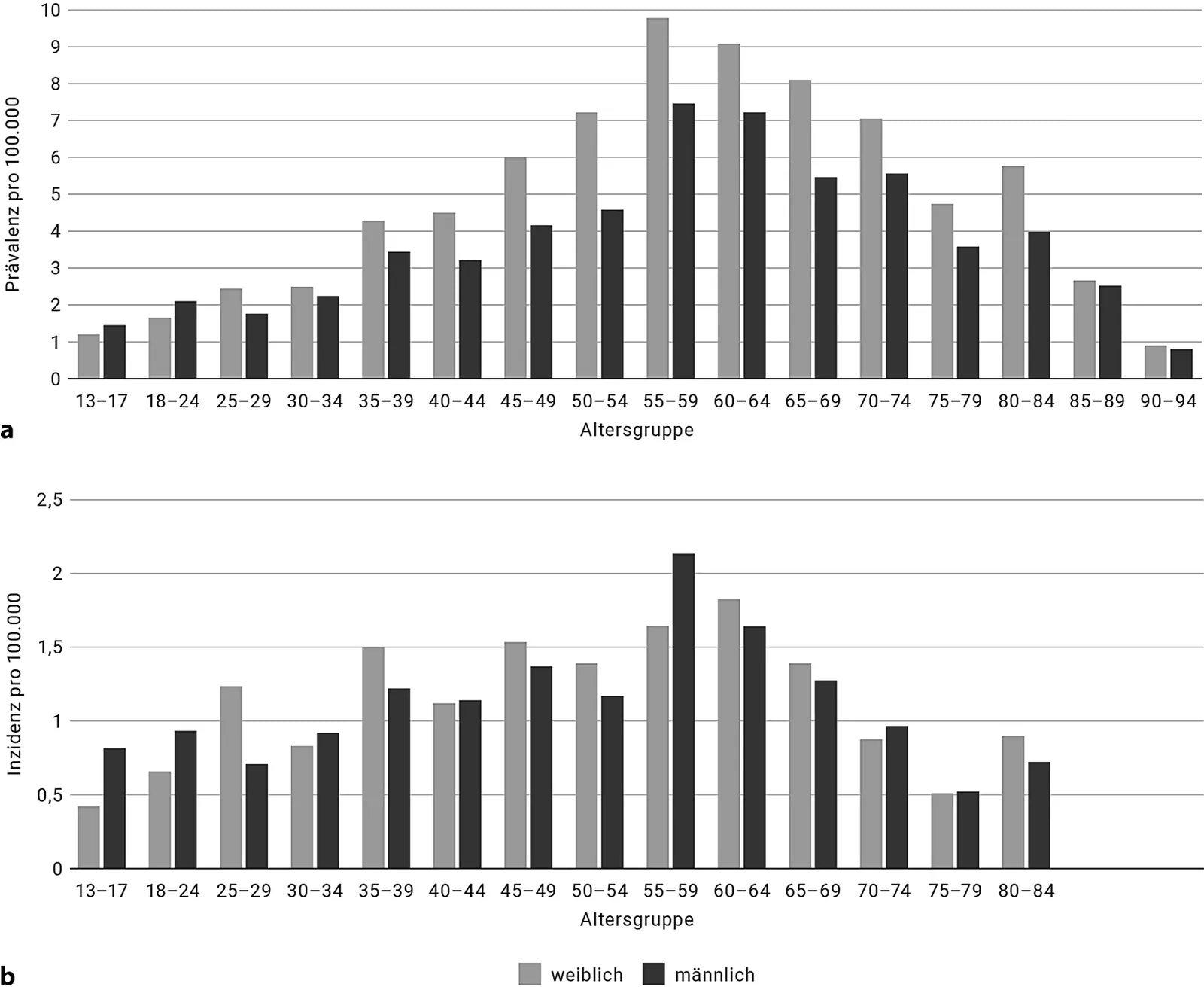
Alters- und geschlechtsspezifische Prävalenz (**a**) und Inzidenz (**b**) von Vitiligo in Deutschland 2023

Im Jahr 2023 umfasste die DADB 2.716.953 Versicherte ≥ 12 Jahre und 2.534.891 ≥ 18 Jahre. Vitiligo wurde bei 3697 Personen ≥ 12 Jahre und 3604 Personen ≥ 18 Jahre diagnostiziert (Tab. 1). Die GKV-adjustierte Prävalenz lag bei 138 (≥ 12 Jahre) und 144 (≥ 18 Jahre) pro 100.000 Personen. Für die Inzidenz wurden 2.612.090 Versicherte ≥ 12 Jahre und 2438.734 ≥ 18 Jahre ausgewertet. Es gab 871 bzw. 828 neu diagnostizierte Vitiligo-Fälle in 2023 (Tab. 1) mit einer GKV-adjustierten Inzidenz von 32 (≥ 12 Jahre) und 33 (≥ 18 Jahre) pro 100.000 Personen. Für Prävalenz und Inzidenz lagen die Höchstwerte im Alter von 55 bis 64 Jahren (Abb. 1). Erstdiagnosen erfolgten überwiegend ambulant durch Dermatologen, Allgemeinmediziner und Innere/hausärztliche Versorgung (Online-Tab. 5).

**Tab. 1:** Prävalenz und Inzidenz von Vitiligo in Deutschland im Jahr 2023

Alterskohorte	Anzahl der Personen mit Vitiligo, DADB	Anzahl der Personen mit Vitiligo, GKV-adjustiert [95 %-KI]	Anteil der Personen mit Vitiligo, GKV-adjustiert (%)	Prävalenz/Inzidenz pro 100.000, GKV-adjustiert
*Prävalenz*				
≥ 12 Jahre	3697	92.352 [89.381–95.450]	0,14	138
≥ 18 Jahre	3604	90.238 [87.297–93.304]	0,14	144
*Inzidenz*				
≥ 12 Jahre	871	21.772 [20.367–23.298]	0,03	32
≥ 18 Jahre	828	20.745 [19.370–22.236]	0,03	33

### Begleiterkrankungen und Behandlungsmuster

Die Ergebnisse zu Begleiterkrankungen und Behandlungsmustern waren in beiden Alterskohorten vergleichbar. Daher werden nur die Ergebnisse für die Population ≥ 12 Jahren genannt (alle Subgruppen in Online-Tab. 6–7).

Von 673 analysierten ICD-10-Diagnosen traten 343 (51,0 %) bei Personen mit Vitiligo signifikant häufiger auf als in der Kontrollkohorte, während nur 2 (0,3 %) signifikant seltener auftraten (OR: < 1; *p* ≤ 0,05) [Online-Tab. 7]. Die breite Komorbiditätslast erstreckte sich über fast alle großen Krankheitsgruppen, besonders Autoimmun- und entzündliche Erkrankungen sowie dermatologische, endokrinologische und psychische bzw. mentale Verhaltensstörungen (Tab. 2; [7, 19, 20]). Besonders starke Assoziationen zeigten sich bei Thyreoiditis (OR: 6,11; 95 %-KI: 5,52–6,75; *p* < 0,001), Alopecia areata (OR: 5,59; 95 %-KI: 3,97–7,86; *p* < 0,001) und Diabetes mellitus Typ 1 (OR: 4,54; 95 %-KI: 3,81–5,42; *p* < 0,001).

**Tab. 2:** Ausgewählte Begleiterkrankungen bei Personen mit Vitiligo

Diagnose	ICD-10-Code	Vitiligo, *n* (%), *N* = 3697	Kontrolle, *n* (%), *N* = 36.970	OR [95 %-KI]	*p*-Wert
*Autoimmun-/endokrine Erkrankungen*					
Thyreoiditis	E06	751 (20,31)	1567 (4,24)	6,11	< 0,001
				[5,52–6,75]	
Hyperthyreose (Thyreotoxikose)	E05	230 (6,22)	1031 (2,79)	2,25	< 0,001
				[1,94–2,61]	
Sonstige Hypothyreose	E03	821 (22,21)	4127 (11,16)	2,25	< 0,001
				[2,06–2,46]	
Sonstige Krankheiten der Schilddrüse	E07	234 (6,33)	1057 (2,86)	2,25	< 0,001
				[1,94–2,61]	
Alopecia areata	L63	53 (1,43)	91 (0,25)	5,59	< 0,001
				[3,97–7,86]	
Diabetes mellitus Typ 1	E10	190 (5,14)	415 (1,12)	4,54	< 0,001
				[3,81–5,42]	
Systemische Sklerose	M34	16 (0,43)	36 (0,10)	4,30	< 0,001
				[2,38–7,75]	
Seropositive chronische Polyarthritis	M05	39 (1,05)	214 (0,58)	1,76	0,001
				[1,25–2,48]	
Sonstige chronische Polyarthritis	M06	149 (4,03)	845 (2,29)	1,74	< 0,001
				[1,45–2,08]	
Arthritis psoriatica und Arthritiden bei gastrointestinalen Grundkrankheiten	M07	38 (1,03)	159 (0,43)	2,32	< 0,001
				[1,62–3,31]	
*Entzündliche Hauterkrankungen/immunologische Hautkomorbiditäten*					
Lichen simplex chronicus und Prurigo	L28	41 (1,11)	157 (0,42)	2,52	< 0,001
				[1,78–3,55]	
Atopisches (endogenes) Ekzem	L20	266 (7,2)	1196 (3,24)	2,20	< 0,001
				[1,92–2,53]	
Psoriasis	L40	257 (6,95)	1280 (3,46)	2,00	< 0,001
				[1,74–2,31]	
Allergische Kontaktdermatitis	L23	118 (3,19)	604 (1,63)	1,91	< 0,001
				[1,56–2,33]	
Sonstige Dermatitis	L30	505 (13,66)	2727 (7,38)	1,90	< 0,001
				[1,72–2,11]	
Urtikaria	L50	102 (2,76)	403 (1,09)	2,46	< 0,001
				[1,98–3,07]	
Seborrhoisches Ekzem	L21	86 (2,33)	439 (1,19)	1,89	< 0,001
				[1,49–2,38]	
*Psychische Komorbiditäten*					
Phobische Störungen	F40	90 (2,43)	504 (1,36)	1,72	< 0,001
				[1,37–2,16]	
Sexuelle Funktionsstörungen, nicht verursacht durch eine organische Störung oder Krankheit	F52	162 (4,38)	995 (2,69)	1,61	< 0,001
				[1,35–1,91]	
Somatoforme Störungen	F45	666 (18,01)	4652 (12,58)	1,46	< 0,001
				[1,33–1,60]	
Andere neurotische Störungen	F48	209 (5,65)	1491 (4,03)	1,35	< 0,001
				[1,16–1,57]	
Depressive Episode	F32	697 (18,85)	5428 (14,68)	1,28	< 0,001
				[1,17–1,40]	

Dargestellte Begleiterkrankungen sind nach Krankheitsgruppen geordnet und zeigen signifikant mit Vitiligo assoziierte Diagnosen. Sie dienen der deskriptiven Abbildung des Komorbiditätsprofils

Im Jahr 2023 erhielten 22,1 % der Prävalenzkohorte eine Vitiligo-Therapie (Tab. 3/Online-Tab. 8). Topische Therapien (16,8 %) waren häufiger als systemische Therapien (6,5 %) und UV-Therapien (0,8 %). Die Prävalenzkohorte wurde zusätzlich nach Fachgruppen und Ausschlussdiagnosen in eine Therapiekohorte unterteilt, da sich Vitiligo-Behandlungen oft mit Therapien anderer Erkrankungen überschneiden. In der Therapiekohorte ist es folglich wahrscheinlicher, dass die erfassten Verordnungen tatsächlich der Behandlung von Vitiligo dienen.

**Tab. 3:** Behandlungsprävalenz von Personen mit Vitiligo

	≥ 12 Jahre		
Populationsgruppe	Anzahl der Personen, DADB	Anteil der Personen in der Prävalenzkohorte (%)	Anteil der prävalenten Personen in der Therapiekohorte (%)
**Prävalenzkohorte**			
*Alle Personen mit Vitiligo*	3697	100,0	–
Behandelt durch beliebige Ärzt*innen	816	22,1	–
– Topische Therapeutika^1^	622	16,8	–
– Systemische Glukokortikosteroide^1^	241	6,5	–
– UV-Therapie^1^	28	0,8	–
**Therapiekohorte**			
*Personen mit Vitiligo ohne Ausschlussdiagnosen*	1310	35,4	100,0
Behandelt durch spezialisierte Fachärzt*innen	107	–	8,2
– Topische Therapeutika^1^	94	–	7,2
– Systemische Glukokortikosteroide^1^	15	–	1,1
– UV-Therapie^1^	< 10	–	–

^1^Personen, die Behandlungen aus mehr als einer Therapieform erhielten, wurden mehrfach erfasst

In dieser Kohorte erhielten nur 8,2 % eine medikamentöse Behandlung – 7,2 % topisch, 1,1 % systemisch und nur wenige Personen eine UV-Therapie. Personen, die Behandlungen aus mehr als einer Kategorie erhielten, wurden mehrfach erfasst.

Bei den eingesetzten Wirkstoffen in der Prävalenzkohorte waren topische Glukokortikosteroide mit 48,9 % die häufigste Option, gefolgt von Calcineurininhibitoren (10,4 %) und topischem Ruxolitinib (7,5 %). Systemische Glukokortikosteroide wurden bei 29,5 %, UV-Therapien bei 3,4 % der behandelten Personen eingesetzt, etwa die Hälfte der UV-Therapien erfolgte kombiniert mit topischen Therapien. Von allen behandelten Personen in der Therapiekohorte erhielten 35,5 % topische Glukokortikosteroide, 19,6 % topisches Ruxolitinib und 18,7 % Calcineurininhibitoren. Systemische Glukokortikosteroide wurden bei 14,0 % verordnet; UV-Therapien waren selten. Ruxolitinib wurde in der Therapiekohorte deutlich häufiger eingesetzt als in der Prävalenzkohorte (19,6 % vs. 7,5 %), während systemische Glukokortikosteroide seltener verordnet wurden (14,0 % vs. 29,5 %) (Tab. 4/Online-Tab. 9).

**Tab. 4:** Übersicht der eingesetzten Therapien bei Personen mit Vitiligo

		Prävalenzkohorte		Therapiekohorte	
		Alle behandelten Personen mit Vitiligo		Behandelte Personen mit Vitiligo ohne Ausschlussdiagnosen, behandelt durch spezialisierte Fachärzt*innen	
Therapieform	Wirkstoffkategorie	Anzahl der Personen, DADB	Anteil der behandelten Personen (%)	Anzahl der Personen, DADB	Anteil der behandelten Personen (%)
*≥12 Jahre*					
Topisch medikamentös^1^	Ruxolitinib und/oder Glukokortikosteroide und/oder Calcineurininhibitoren	622	76,2	94	87,9
	Glukokortikosteroide und/oder Calcineurininhibitoren	561	68,8	73	68,2
	Nur Glukokortikosteroide	399	48,9	38	35,5
	Nur Calcineurininhibitoren	85	10,4	20	18,7
	Nur Ruxolitinib	61	7,5	21	19,6
Systemisch medikamentös^1^	Systemische Glukokortikosteroide	241	29,5	15	14,0
UV-Therapie^1^	UV-Therapie gesamt	28	3,4	< 10	–
	UV- und topische Therapeutika	14	1,7	0	0,0
	UV-Therapie und systemische Glukokortikosteroide	< 10	–	0	0,0

^1^Personen, die Behandlungen aus mehr als einer Therapieform erhielten, wurden mehrfach erfasst

## Diskussion

Die Analyse zeigt anhand deutscher Krankenkassendaten aus 2023 die Versorgungssituation von Personen mit Vitiligo. Die GKV-adjustierte Prävalenz lag bei 0,14 %. Betroffene wiesen deutlich häufiger Begleiterkrankungen auf als die Normalbevölkerung; 22,1 % erhielten eine Therapie mit nochmals niedrigeren Raten in der Therapiekohorte.

Die Prävalenz von Vitiligo in Deutschland liegt im unteren Bereich internationaler Schätzungen von 0,5–2,0 % [2, 4, 18, 23]. Ein direkter Vergleich mit einzelnen Studien ist jedoch nur eingeschränkt möglich, da je nach Studiendesign unterschiedliche Altersgruppen oder Falldefinitionen zugrunde liegen. Diese Analyse beschränkt sich auf Personen im Alter von ≥ 12 Jahren.

Unsere Ergebnisse decken sich mit einer aktuellen Auswertung von Krankenkassendaten aus 2016 bis 2020. Interessant ist, dass die 3‑Jahres-Prävalenz nur wenig höher als die 1‑Jahres-Prävalenz ausfällt. Die Werte sind also unabhängig von der Falldefinition vergleichbar [16].

Die Prävalenz zeigte eine altersabhängige Verteilung mit Höchstwerten zwischen 55 und 64 Jahren, was den publizierten Daten entspricht [23]. Die Inzidenzrate von 32 bzw. 33 pro 100.000 Personen im Jahr 2023 liegt im Rahmen bisheriger globaler Inzidenzen von 16 bis 62 pro 100.000 Personenjahren [1].

Das mittlere Alter bei Krankheitsbeginn wird in der Literatur mit 24 bis 33 Jahren angegeben [3, 4, 11, 29], während in unserer Analyse die höchste Inzidenz in der Altersgruppe der 55- bis 64-Jährigen lag. Eine Untererfassung ist möglich, da durchschnittlich 2,4 Jahre bis zur Diagnose vergehen [3] und bis zu 40 % der Fälle unerkannt bleiben [4].

Etwa 15–20 % der Betroffenen haben mindestens eine begleitende Autoimmunerkrankung [14, 28], besonders Thyreoiditis, die mit verstärkter Vitiligo-Manifestation zusammenhängen kann [7, 19, 20, 28]. Zudem besteht eine erhöhte Komorbidität mit entzündlichen Dermatosen, was auf eine überschneidende Prädisposition für chronisch inflammatorische Hauterkrankungen hinweist [7, 12, 19].

Klinisch relevant sind psychische Begleiterkrankungen wie depressive Episoden, Angst- und somatoforme Störungen sowie sexuelle Funktionsstörungen [2, 10, 12]. Diese Komorbiditäten können zu reduzierter sozialer Teilnahme, eingeschränkter Lebensqualität und erhöhtem Suizidrisiko führen [3, 4, 8, 10]. Die Ergebnisse werden auch durch Klinger et al. gestützt, die ebenfalls ein erhöhtes Risiko für Autoimmunerkrankungen wie Alopecia areata und für psychische Störungen zeigen [16].

Die vorliegenden Daten unterstreichen neben dem in der S1-Leitlinie [5] empfohlenen Schilddrüsenscreening auch die mögliche Relevanz einer Abklärung weiterer dermatologischer, psychischer oder autoimmuner Erkrankungen. Aufgrund des explorativen Charakters der Analyse lassen sich jedoch in unserer Abrechnungsdatenanalyse keine generellen Screeningempfehlungen ableiten; diese werden in der kürzlich erschienenen S2k-Leitlinie „Diagnostik und Therapie der Vitiligo“ (AWMF-Register-Nr.: 013-093) und der in Arbeit befindlichen S3-Leitlinie „Psychosomatische Dermatologie“ (AWMF-Register-Nr.: 013-024) spezifiziert. Sinnvoll erscheint immer eine risikoadaptierte und individuelle Abklärung je nach Anamnese und klinischen Symptomen.

Im Jahr 2023 erhielten 22,1 % aller Personen mit Vitiligo eine Behandlung. Da viele Betroffene zusätzlich an immunologischen oder autoimmunen Erkrankungen leiden (Online-Tab. 6) und Therapien für Vitiligo meist unspezifisch sind, wurden die Behandlungsraten nach Fachgruppen und Ausschlussdiagnosen stratifiziert (Therapiekohorte), um gezielte Vitiligo-Behandlungen zu identifizieren. Dabei sank die Behandlungsrate auf 8,2 % (≥ 12 Jahre), was auf eine geringe Zahl gezielter Behandlungen hindeutet. Gründe können milde oder stabile Krankheitsverläufe, eine geringe subjektive Krankheitslast oder ein eingeschränkter Zugang zu Spezialisten sein [4, 27]. Eine aktuelle Umfrage unter niedergelassenen deutschen Dermatologen zeigte, dass Vitiligo oft als kosmetisches Problem angesehen wird und leitliniengerechte Diagnostik und Therapie unzureichend umgesetzt werden [22].

Die niedrige Behandlungsrate könnte durch kosmetische oder frei verkäufliche Präparate bedingt sein, die in Abrechnungsdaten nicht erfasst werden [26]. In einer aktuellen Patientenbefragung erhielten nur 12,4 % der Betroffenen keine Behandlung [11]. Die Unterschiede resultieren vermutlich aus methodischen Aspekten, da Befragungen frei verkäufliche Produkte berücksichtigen und eher therapieerfahrene Betroffene erreichen. Da wirksame Behandlungen die Lebensqualität verbessern können [24], weisen die Ergebnisse auf eine relevante Versorgungslücke hin.

Die deutsche S1-Leitlinie empfiehlt topische antiinflammatorische Therapien wie Glukokortikosteroide und Calcineurininhibitoren als zentrale medikamentöse Optionen bei Vitiligo [5]. In der Therapiekohorte erhielten nur 35,5 % topische Glukokortikosteroide gegenüber 48,9 % in der Prävalenzkohorte, was auf nicht Vitiligo-spezifische Verordnungen hindeutet. Systemische Glukokortikosteroide wurden seltener eingesetzt (14,0 % bzw. 29,5 %) und kommen nur für eine kleine Patientengruppe infrage, etwa bei rasch fortschreitenden Verläufen [5]. Eine Ganzkörperphototherapie mit UV-B-Schmalband (selektive UV-B-Phototherapie) ist bei ausgedehnter und/oder rasch fortschreitender Verlaufsform der NSV empfohlen [5], wurde in den vorliegenden Abrechnungsdaten aber überraschend selten dokumentiert. Dabei ist zu berücksichtigen, dass nur über die GKV abgerechnete Leistungen auswertbar waren.

Neue zielgerichtete Therapien wie Ruxolitinib wurden in der Prävalenzkohorte selten (7,5 %), in der Therapiekohorte häufiger (19,6 %) verordnet, was auf einen gezielten Einsatz bei Vitiligo hindeutet. Ruxolitinib ist erst seit April 2023 für NSV ab 12 Jahren zugelassen [9], was eine Implementierung dieser innovativen Behandlung zeigt, die auch für stärker Betroffene eine potenzielle Alternative zu konventionellen topischen Therapien darstellt.

Die Analyse basiert auf Abrechnungsdaten von 14 gesetzlichen Krankenkassen mit über 2,7 (≥ 12 Jahre) bzw. über 2,5 (≥ 18 Jahre) Millionen Versicherten. Dadurch ließ sich eine repräsentative Population von Personen mit Vitiligo beschreiben, die Demografie, weitere Diagnosen und Verordnungen sowie die Versorgungssituation in Deutschland realistisch widerspiegelt. Eine gematchte Kontrollgruppe ermöglichte den Vergleich von Komorbiditätsprofilen.

Die Studie hat methodische Limitationen: Die Daten enthalten keine klinischen Details zur Krankheitsschwere oder zu Behandlungsergebnissen. Eine entsprechende Stratifizierung war daher nicht möglich, obwohl schwerere Verläufe eher behandelt werden [26]. Fehlkodierungen oder inkonsistente Dokumentation können Prävalenz- und Behandlungsraten unterschätzen. Die Anwendung der M2Q-Kriterien erhöht die diagnostische Spezifität, kann jedoch zu einer konservativen Schätzung der Prävalenz führen. Das querschnittliche Studiendesign erlaubt nur Assoziationen, keine Kausalitäten. Ergebnisse zu Begleiterkrankungen sind ohne Adjustierung für multiples Testen als explorativ zu betrachten. Zudem war eine Unterscheidung zwischen NSV und der segmentalen Vitiligo-Form aufgrund des gemeinsamen ICDs nicht möglich.

## Fazit für die Praxis

- Die Ergebnisse zeigen, dass Vitiligo häufig mit vielfältigen Begleiterkrankungen verbunden ist.
- Ein systematisches Screening ist wichtig, um diese früh zu erkennen und angemessen zu behandeln.
- Nur wenige Betroffene erhalten eine dokumentierte Vitiligo-Therapie, was auf eine unzureichende Versorgungsituation hinweist.
- Künftige Studien sollten diese Versorgungslücken genauer und flächendeckender analysieren und Strategien zur Verbesserung des Zugangs zu wirksamen Behandlungen entwickeln.

## Supplementary Information

ESM1: Zusatzmaterial 1

## Data Availability

Die in dieser Studie erhobenen Datensätze können auf begründete Anfrage beim Korrespondenzautor angefordert werden.
