# Supplementary material for: Vitiligo in Germany: A claims data analysis on prevalence, comorbidity, and treatment patterns
Source: Dermatologie (Heidelb). 2026 Aug 24;77(10):673–80. [Article in German] doi: 10.1007/s00105-026-05764-5 (PMC13631043; doi:10.1007/s00105-026-05764-5)
Supplement: Supplementary file 1 [file 105_2026_5764_MOESM1_ESM.pdf]

Online-Tabellen 1 bis 9

Online-Tabelle 1 – ATC-Codes zur Identifizierung von Calcineurininhibitoren und JAK-Inhibitoren

	Wirkstoff	ATC-Code*
Calcineurininhibitoren	Tacrolimus	D11AH01
	Pimecrolimus	D11AH02
JAK-Inhibitoren	Ruxolitinib	D11AH09

Online-Tabelle 2 – OPS- und EBM-Codes zur Identifizierung von Phototherapie

	EBM*-Code		OPS*-Code
Selektive Phototherapie	30430	PUVA	8-560.1
Zuschlag für Photochemotherapie	30431	Excimerlaser	5-985.4
Balneophototherapie	10350	Salzphototherapie	8-560.5
		Selektive UV-Phototherapie	8-560.0
		Lichttherapie, nicht näher bezeichnet	8-560.y
		UVA	8-560.3

*EBM (Einheitlicher Bewertungsmaßstab); OPS (Operationen- und Prozedurenschlüssel)

Online-Tabelle 3 – PZN-Codes zur Identifizierung systemischer Glukokortikoide

PZN	Artikelbezeichnung	Wirkstoff / Menge	Hersteller
6846637	DEXAMETHASONE acis 4 mg Tabletten	Dexamethason 4 mg	Acis
745450	DEXAMETHASONE 0,5 mg GALEN Tabletten	Dexamethason 0,5 mg	Galenpharma
745473	DEXAMETHASONE 0,5 mg GALEN Tabletten	Dexamethason 0,5 mg	Galenpharma
745496	DEXAMETHASONE 0,5 mg GALEN Tabletten	Dexamethason 0,5 mg	Galenpharma
4007832	DEXAMETHASONE 0,5 mg JENAPHARM Tabletten	Dexamethason 0,5 mg	Mibe
6342894	DEXAMETHASONE 0,5 mg JENAPHARM Tabletten	Dexamethason 0,5 mg	Mibe
14366035	DEXAMETHASONE TAD 0,5 mg Tabletten	Dexamethason 0,5 mg	TAD Pharma
14294169	DEXAMETHASONE TAD 0,5 mg Tabletten	Dexamethason 0,5 mg	TAD Pharma
14294175	DEXAMETHASONE TAD 0,5 mg Tabletten	Dexamethason 0,5 mg	TAD Pharma
14294206	DEXAMETHASONE TAD 0,5 mg Tabletten	Dexamethason 0,5 mg	TAD Pharma
4862431	FORTECORTIN 0,5 mg Tabletten	Dexamethason 0,5 mg	Merck Healthc.
4862448	FORTECORTIN 0,5 mg Tabletten	Dexamethason 0,5 mg	Merck Healthc.
4862454	FORTECORTIN 0,5 mg Tabletten	Dexamethason 0,5 mg	Merck Healthc.
745504	DEXAMETHASONE 1,5 mg GALEN Tabletten	Dexamethason 1,5 mg	Galenpharma
745510	DEXAMETHASONE 1,5 mg GALEN Tabletten	Dexamethason 1,5 mg	Galenpharma
745527	DEXAMETHASONE 1,5 mg GALEN Tabletten	Dexamethason 1,5 mg	Galenpharma
4007849	DEXAMETHASONE 1,5 mg JENAPHARM Tabletten	Dexamethason 1,5 mg	Mibe
6342902	DEXAMETHASONE 1,5 mg JENAPHARM Tabletten	Dexamethason 1,5 mg	Mibe
2452829	FORTECORTIN 2 mg Tabletten	Dexamethason 2 mg	Merck Healthc.
2452835	FORTECORTIN 2 mg Tabletten	Dexamethason 2 mg	Merck Healthc.
2452841	FORTECORTIN 2 mg Tabletten	Dexamethason 2 mg	Merck Healthc.
13721907	DEXAMETHASONE TAD 20 mg Tabletten	Dexamethason 20 mg	TAD Pharma
15571435	DEXAMETHASONE TAD 20 mg Tabletten	Dexamethason 20 mg	TAD Pharma
13721913	DEXAMETHASONE TAD 20 mg Tabletten	Dexamethason 20 mg	TAD Pharma
13721936	DEXAMETHASONE TAD 20 mg Tabletten	Dexamethason 20 mg	TAD Pharma
745740	DEXAMETHASONE 4 mg GALEN Tabletten	Dexamethason 4 mg	Galenpharma
745651	DEXAMETHASONE 4 mg GALEN Tabletten	Dexamethason 4 mg	Galenpharma
745645	DEXAMETHASONE 4 mg GALEN Tabletten	Dexamethason 4 mg	Galenpharma

8918803	DEXAMETHASONE 4 mg JENAPHARM Tabletten	Dexamethason 4 mg	Mibe
14062588	DEXAMETHASONE 4 mg JENAPHARM Tabletten	Dexamethason 4 mg	Mibe
8918826	DEXAMETHASONE 4 mg JENAPHARM Tabletten	Dexamethason 4 mg	Mibe
8918832	DEXAMETHASONE 4 mg JENAPHARM Tabletten	Dexamethason 4 mg	Mibe
6846637	DEXAMETHASONE acis 4 mg Tabletten	Dexamethason 4 mg	Acis
6846643	DEXAMETHASONE acis 4 mg Tabletten	Dexamethason 4 mg	Acis
13754367	DEXAMETHASONE TAD 4 mg Tabletten	Dexamethason 4 mg	TAD Pharma
13754373	DEXAMETHASONE TAD 4 mg Tabletten	Dexamethason 4 mg	TAD Pharma
13754404	DEXAMETHASONE TAD 4 mg Tabletten	Dexamethason 4 mg	TAD Pharma
2214739	DEXAMETHASONE-ratiopharm 4 mg Tabletten	Dexamethason 4 mg	ratiopharm
2214751	DEXAMETHASONE-ratiopharm 4 mg Tabletten	Dexamethason 4 mg	ratiopharm
2214768	DEXAMETHASONE-ratiopharm 4 mg Tabletten	Dexamethason 4 mg	ratiopharm
4862508	FORTECORTIN 4 mg Tabletten	Dexamethason 4 mg	Merck Healthc.
14191357	FORTECORTIN 4 mg Tabletten	Dexamethason 4 mg	Merck Healthc.
4862514	FORTECORTIN 4 mg Tabletten	Dexamethason 4 mg	Merck Healthc.
2469273	FORTECORTIN 4 mg Tabletten	Dexamethason 4 mg	Merck Healthc.
13721942	DEXAMETHASONE TAD 40 mg Tabletten	Dexamethason 40 mg	TAD Pharma
15319397	DEXAMETHASONE TAD 40 mg Tabletten	Dexamethason 40 mg	TAD Pharma
13721959	DEXAMETHASONE TAD 40 mg Tabletten	Dexamethason 40 mg	TAD Pharma
13721965	DEXAMETHASONE TAD 40 mg Tabletten	Dexamethason 40 mg	TAD Pharma
745786	DEXAMETHASONE 8 mg GALEN Tabletten	Dexamethason 8 mg	Galenpharma
745763	DEXAMETHASONE 8 mg GALEN Tabletten	Dexamethason 8 mg	Galenpharma
745757	DEXAMETHASONE 8 mg GALEN Tabletten	Dexamethason 8 mg	Galenpharma
1436478	DEXAMETHASONE 8 mg JENAPHARM Tabletten	Dexamethason 8 mg	Mibe
1436484	DEXAMETHASONE 8 mg JENAPHARM Tabletten	Dexamethason 8 mg	Mibe
14028947	DEXAMETHASONE 8 mg JENAPHARM Tabletten	Dexamethason 8 mg	Mibe
1436490	DEXAMETHASONE 8 mg JENAPHARM Tabletten	Dexamethason 8 mg	Mibe
1436509	DEXAMETHASONE 8 mg JENAPHARM Tabletten	Dexamethason 8 mg	Mibe
6846672	DEXAMETHASONE acis 8 mg Tabletten	Dexamethason 8 mg	Acis
6846689	DEXAMETHASONE acis 8 mg Tabletten	Dexamethason 8 mg	Acis
13754410	DEXAMETHASONE TAD 8 mg Tabletten	Dexamethason 8 mg	TAD Pharma

13754456	DEXAMETHASONE TAD 8 mg Tabletten	Dexamethason 8 mg	TAD Pharma
13754427	DEXAMETHASONE TAD 8 mg Tabletten	Dexamethason 8 mg	TAD Pharma
2214685	DEXAMETHASONE-ratiopharm 8 mg Tabletten	Dexamethason 8 mg	ratiopharm
2214691	DEXAMETHASONE-ratiopharm 8 mg Tabletten	Dexamethason 8 mg	ratiopharm
2214716	DEXAMETHASONE-ratiopharm 8 mg Tabletten	Dexamethason 8 mg	ratiopharm
7449944	FORTECORTIN 8 mg Tabletten	Dexamethason 8 mg	Merck Healthc.
14191363	FORTECORTIN 8 mg Tabletten	Dexamethason 8 mg	Merck Healthc.
7449950	FORTECORTIN 8 mg Tabletten	Dexamethason 8 mg	Merck Healthc.
7449967	FORTECORTIN 8 mg Tabletten	Dexamethason 8 mg	Merck Healthc.
7509072	FORTECORTIN 8 mg Tabletten	Dexamethason 8 mg	Merck Healthc.
17944685	DEXAMETHASONE GALEN 2 mg/ml Lösung zum Einnehmen	Dexamethason sodium phosphate 2,63 mg	Galenpharma
17955938	DEXAMETHASONE GALEN 2 mg/ml Lösung zum Einnehmen	Dexamethason sodium phosphate 2,63 mg	Galenpharma
17955944	DEXAMETHASONE GALEN 2 mg/ml Lösung zum Einnehmen	Dexamethason sodium phosphate 2,63 mg	Galenpharma
17944691	DEXAMETHASONE GALEN 4 mg/ml Lösung zum Einnehmen	Dexamethason sodium phosphate 5,26 mg	Galenpharma
17955950	DEXAMETHASONE GALEN 4 mg/ml Lösung zum Einnehmen	Dexamethason sodium phosphate 5,26 mg	Galenpharma
17955967	DEXAMETHASONE GALEN 4 mg/ml Lösung zum Einnehmen	Dexamethason sodium phosphate 5,26 mg	Galenpharma
2562015	M PREDNIHEXAL 16 mg Tabletten	MethylPrednisolon 16 mg	Hexal
2562021	M PREDNIHEXAL 16 mg Tabletten	MethylPrednisolon 16 mg	Hexal
2562038	M PREDNIHEXAL 16 mg Tabletten	MethylPrednisolon 16 mg	Hexal
8424231	METHYLPREDNISOLONEE 16 mg Jenapharm Tabletten	MethylPrednisolon 16 mg	Mibe
8424248	METHYLPREDNISOLONEE 16 mg Jenapharm Tabletten	MethylPrednisolon 16 mg	Mibe
8424254	METHYLPREDNISOLONEE 16 mg Jenapharm Tabletten	MethylPrednisolon 16 mg	Mibe
8424260	METHYLPREDNISOLONEE 16 mg Jenapharm Tabletten	MethylPrednisolon 16 mg	Mibe
12340988	METYPRED 16 mg GALEN Tabletten	MethylPrednisolon 16 mg	Galenpharma
1484431	METYPRED 16 mg GALEN Tabletten	MethylPrednisolon 16 mg	Galenpharma
12340994	METYPRED 16 mg GALEN Tabletten	MethylPrednisolon 16 mg	Galenpharma
1484460	METYPRED 16 mg GALEN Tabletten	MethylPrednisolon 16 mg	Galenpharma
1484477	METYPRED 16 mg GALEN Tabletten	MethylPrednisolon 16 mg	Galenpharma
7567023	PREDNI M Tablinen 16 mg Tabletten	MethylPrednisolon 16 mg	Zentiva Pharma
7567046	PREDNI M Tablinen 16 mg Tabletten	MethylPrednisolon 16 mg	Zentiva Pharma
3444973	PREDNI M Tablinen 16 mg Tabletten	MethylPrednisolon 16 mg	Zentiva Pharma

7567052	PREDNI M Tablinen 16 mg Tabletten	MethylPrednisolon 16 mg	Zentiva Pharma
7567069	PREDNI M Tablinen 16 mg Tabletten	MethylPrednisolon 16 mg	Zentiva Pharma
1971361	URBASON 16 mg Tabletten	MethylPrednisolon 16 mg	Fidia Pharma
1971378	URBASON 16 mg Tabletten	MethylPrednisolon 16 mg	Fidia Pharma
2511414	URBASON 16 mg Tabletten	MethylPrednisolon 16 mg	Fidia Pharma
1451124	URBASON 16 mg Tabletten	MethylPrednisolon 16 mg	Docpharm
1457687	URBASON 16 mg Tabletten	MethylPrednisolon 16 mg	Docpharm
4881641	METHYLPREDNISOLONEE 32 mg Jenapharm Tabletten	MethylPrednisolon 32 mg	Mibe
4881664	METHYLPREDNISOLONEE 32 mg Jenapharm Tabletten	MethylPrednisolon 32 mg	Mibe
4887827	METHYLPREDNISOLONEE 32 mg Jenapharm Tabletten	MethylPrednisolon 32 mg	Mibe
2562127	M PREDNIHEXAL 4 mg Tabletten	MethylPrednisolon 4 mg	Hexal
2562133	M PREDNIHEXAL 4 mg Tabletten	MethylPrednisolon 4 mg	Hexal
2562156	M PREDNIHEXAL 4 mg Tabletten	MethylPrednisolon 4 mg	Hexal
8424136	METHYLPREDNISOLONEE 4 mg Jenapharm Tabletten	MethylPrednisolon 4 mg	Mibe
8424142	METHYLPREDNISOLONEE 4 mg Jenapharm Tabletten	MethylPrednisolon 4 mg	Mibe
14141419	METHYLPREDNISOLONEE 4 mg Jenapharm Tabletten	MethylPrednisolon 4 mg	Mibe
8424159	METHYLPREDNISOLONEE 4 mg Jenapharm Tabletten	MethylPrednisolon 4 mg	Mibe
8424165	METHYLPREDNISOLONEE 4 mg Jenapharm Tabletten	MethylPrednisolon 4 mg	Mibe
12340959	METYPRED 4 mg GALEN Tabletten	MethylPrednisolon 4 mg	Galenpharma
1484371	METYPRED 4 mg GALEN Tabletten	MethylPrednisolon 4 mg	Galenpharma
1484388	METYPRED 4 mg GALEN Tabletten	MethylPrednisolon 4 mg	Galenpharma
1484394	METYPRED 4 mg GALEN Tabletten	MethylPrednisolon 4 mg	Galenpharma
7516818	PREDNI M Tablinen 4 mg Tabletten	MethylPrednisolon 4 mg	Zentiva Pharma
4969181	PREDNI M Tablinen 4 mg Tabletten	MethylPrednisolon 4 mg	Zentiva Pharma
4969198	PREDNI M Tablinen 4 mg Tabletten	MethylPrednisolon 4 mg	Zentiva Pharma
3831902	PREDNI M Tablinen 4 mg Tabletten	MethylPrednisolon 4 mg	Zentiva Pharma
1064663	URBASON 4 mg Tabletten	MethylPrednisolon 4 mg	Fidia Pharma
1064686	URBASON 4 mg Tabletten	MethylPrednisolon 4 mg	Fidia Pharma
4784197	URBASON 4 mg Tabletten	MethylPrednisolon 4 mg	Docpharm
3416735	URBASON 4 mg Tabletten	MethylPrednisolon 4 mg	Emra Med
3416741	URBASON 4 mg Tabletten	MethylPrednisolon 4 mg	Emra Med

4966484	URBASON 4 mg Tabletten	MethylPrednisolon 4 mg	kohlpharma
4966490	URBASON 4 mg Tabletten	MethylPrednisolon 4 mg	kohlpharma
3463835	URBASON 4 mg Tabletten B	MethylPrednisolon 4 mg	Docpharm
6443342	METYPRED 40 mg GALEN Tabletten	MethylPrednisolon 40 mg	Galenpharma
6443359	METYPRED 40 mg GALEN Tabletten	MethylPrednisolon 40 mg	Galenpharma
6443365	METYPRED 40 mg GALEN Tabletten	MethylPrednisolon 40 mg	Galenpharma
1064723	URBASON 40 mg Tabletten	MethylPrednisolon 40 mg	Fidia Pharma
2725718	URBASON 40 mg Tabletten	MethylPrednisolon 40 mg	Fidia Pharma
4759928	URBASON 40 mg Tabletten	MethylPrednisolon 40 mg	Fidia Pharma
1064746	URBASON 40 mg Tabletten	MethylPrednisolon 40 mg	Fidia Pharma
4966509	URBASON 40 mg Tabletten	MethylPrednisolon 40 mg	kohlpharma
4966515	URBASON 40 mg Tabletten	MethylPrednisolon 40 mg	kohlpharma
4966521	URBASON 40 mg Tabletten	MethylPrednisolon 40 mg	kohlpharma
3513214	URBASON 40 mg Tabletten B	MethylPrednisolon 40 mg	Docpharm
7242278	URBASON 40 mg Tabletten B	MethylPrednisolon 40 mg	Docpharm
3513220	URBASON 40 mg Tabletten B	MethylPrednisolon 40 mg	Docpharm
2562050	M PREDNIHEXAL 8 mg Tabletten	MethylPrednisolon 8 mg	Hexal
2562067	M PREDNIHEXAL 8 mg Tabletten	MethylPrednisolon 8 mg	Hexal
2562096	M PREDNIHEXAL 8 mg Tabletten	MethylPrednisolon 8 mg	Hexal
8424188	METHYLPREDNISOLONEE 8 mg Jenapharm Tabletten	MethylPrednisolon 8 mg	Mibe
8424194	METHYLPREDNISOLONEE 8 mg Jenapharm Tabletten	MethylPrednisolon 8 mg	Mibe
8424202	METHYLPREDNISOLONEE 8 mg Jenapharm Tabletten	MethylPrednisolon 8 mg	Mibe
8424219	METHYLPREDNISOLONEE 8 mg Jenapharm Tabletten	MethylPrednisolon 8 mg	Mibe
12340971	METYPRED 8 mg GALEN Tabletten	MethylPrednisolon 8 mg	Galenpharma
1484402	METYPRED 8 mg GALEN Tabletten	MethylPrednisolon 8 mg	Galenpharma
13891532	METYPRED 8 mg GALEN Tabletten	MethylPrednisolon 8 mg	Galenpharma
1484419	METYPRED 8 mg GALEN Tabletten	MethylPrednisolon 8 mg	Galenpharma
1484425	METYPRED 8 mg GALEN Tabletten	MethylPrednisolon 8 mg	Galenpharma
8693675	PREDNI M Tablinen 8 mg Tabletten	MethylPrednisolon 8 mg	Zentiva Pharma
8693681	PREDNI M Tablinen 8 mg Tabletten	MethylPrednisolon 8 mg	Zentiva Pharma
8693698	PREDNI M Tablinen 8 mg Tabletten	MethylPrednisolon 8 mg	Zentiva Pharma

8693706	PREDNI M Tablinen 8 mg Tabletten	MethylPrednisolon 8 mg	Zentiva Pharma
4862135	DECORTIN H 1 mg Tabletten	Prednisolon 1 mg	Merck Healthc.
4862141	DECORTIN H 1 mg Tabletten	Prednisolon 1 mg	Merck Healthc.
3740080	DECORTIN H 1 mg Tabletten	Prednisolon 1 mg	Merck Healthc.
4022398	PREDNISOLONE 1 mg Jenapharm Tabletten	Prednisolon 1 mg	Mibe
6343008	PREDNISOLONE 1 mg Jenapharm Tabletten	Prednisolon 1 mg	Mibe
4821484	PREDNISOLONE 1 mg Jenapharm Tabletten	Prednisolon 1 mg	Mibe
2471407	DECORTIN H 10 mg Tabletten	Prednisolon 10 mg	Merck Healthc.
14191239	DECORTIN H 10 mg Tabletten	Prednisolon 10 mg	Merck Healthc.
2471413	DECORTIN H 10 mg Tabletten	Prednisolon 10 mg	Merck Healthc.
2471436	DECORTIN H 10 mg Tabletten	Prednisolon 10 mg	Merck Healthc.
2471442	DECORTIN H 10 mg Tabletten	Prednisolon 10 mg	Merck Healthc.
2561949	PREDNIHEXAL 10 mg Tabletten	Prednisolon 10 mg	Hexal
5004818	PREDNISOLONE 10 mg GALEN Tabletten	Prednisolon 10 mg	Galenpharma
5004847	PREDNISOLONE 10 mg GALEN Tabletten	Prednisolon 10 mg	Galenpharma
5005048	PREDNISOLONE 10 mg GALEN Tabletten	Prednisolon 10 mg	Galenpharma
2065110	PREDNISOLONE 10 mg Jenapharm Tabletten	Prednisolon 10 mg	Mibe
2065127	PREDNISOLONE 10 mg Jenapharm Tabletten	Prednisolon 10 mg	Mibe
2065133	PREDNISOLONE 10 mg Jenapharm Tabletten	Prednisolon 10 mg	Mibe
2107854	PREDNISOLONE ACIS 10 mg Tabletten	Prednisolon 10 mg	Acis
2107877	PREDNISOLONE ACIS 10 mg Tabletten	Prednisolon 10 mg	Acis
2107908	PREDNISOLONE ACIS 10 mg Tabletten	Prednisolon 10 mg	Acis
4208364	PREDNISOLONE AL 10 mg Tabletten	Prednisolon 10 mg	Aliud
4208370	PREDNISOLONE AL 10 mg Tabletten	Prednisolon 10 mg	Aliud
4208401	PREDNISOLONE AL 10 mg Tabletten	Prednisolon 10 mg	Aliud
7625996	PREDNISOLONE STADA 10 mg Tabletten	Prednisolon 10 mg	Stadapharm
7626004	PREDNISOLONE STADA 10 mg Tabletten	Prednisolon 10 mg	Stadapharm
7626010	PREDNISOLONE STADA 10 mg Tabletten	Prednisolon 10 mg	Stadapharm
4143340	PREDNISOLONE 2 mg GALEN Tabletten	Prednisolon 2 mg	Galenpharma
745792	PREDNISOLONE 2 mg GALEN Tabletten	Prednisolon 2 mg	Galenpharma
745800	PREDNISOLONE 2 mg GALEN Tabletten	Prednisolon 2 mg	Galenpharma

3740045	DECORTIN H 20 mg Tabletten	Prednisolon 20 mg	Merck Healthc.
14191245	DECORTIN H 20 mg Tabletten	Prednisolon 20 mg	Merck Healthc.
4862187	DECORTIN H 20 mg Tabletten	Prednisolon 20 mg	Merck Healthc.
3740068	DECORTIN H 20 mg Tabletten	Prednisolon 20 mg	Merck Healthc.
913657	DERMOSOLON 20 mg Tabletten	Prednisolon 20 mg	Dermapharm
913663	DERMOSOLON 20 mg Tabletten	Prednisolon 20 mg	Dermapharm
913686	DERMOSOLON 20 mg Tabletten	Prednisolon 20 mg	Dermapharm
677180	PREDNI H Tablinen 20 mg Tabletten	Prednisolon 20 mg	Zentiva Pharma
677197	PREDNI H Tablinen 20 mg Tabletten	Prednisolon 20 mg	Zentiva Pharma
677205	PREDNI H Tablinen 20 mg Tabletten	Prednisolon 20 mg	Zentiva Pharma
677211	PREDNI H Tablinen 20 mg Tabletten	Prednisolon 20 mg	Zentiva Pharma
2561895	PREDNIHEXAL 20 mg Tabletten	Prednisolon 20 mg	Hexal
2561903	PREDNIHEXAL 20 mg Tabletten	Prednisolon 20 mg	Hexal
15629519	PREDNISOLONE 20 mg GALEN Tabletten	Prednisolon 20 mg	Galenpharma
1484537	PREDNISOLONE 20 mg GALEN Tabletten	Prednisolon 20 mg	Galenpharma
1484543	PREDNISOLONE 20 mg GALEN Tabletten	Prednisolon 20 mg	Galenpharma
1484566	PREDNISOLONE 20 mg GALEN Tabletten	Prednisolon 20 mg	Galenpharma
235803	PREDNISOLONE 20 mg Jenapharm Tabletten	Prednisolon 20 mg	Mibe
235826	PREDNISOLONE 20 mg Jenapharm Tabletten	Prednisolon 20 mg	Mibe
14142005	PREDNISOLONE 20 mg Jenapharm Tabletten	Prednisolon 20 mg	Mibe
235832	PREDNISOLONE 20 mg Jenapharm Tabletten	Prednisolon 20 mg	Mibe
235849	PREDNISOLONE 20 mg Jenapharm Tabletten	Prednisolon 20 mg	Mibe
985148	PREDNISOLONE ACIS 20 mg Tabletten	Prednisolon 20 mg	Acis
1401385	PREDNISOLONE ACIS 20 mg Tabletten	Prednisolon 20 mg	Acis
985154	PREDNISOLONE ACIS 20 mg Tabletten	Prednisolon 20 mg	Acis
985160	PREDNISOLONE ACIS 20 mg Tabletten	Prednisolon 20 mg	Acis
4216085	PREDNISOLONE AL 20 mg Tabletten	Prednisolon 20 mg	Aliud
4216091	PREDNISOLONE AL 20 mg Tabletten	Prednisolon 20 mg	Aliud
4216116	PREDNISOLONE AL 20 mg Tabletten	Prednisolon 20 mg	Aliud
4216139	PREDNISOLONE AL 20 mg Tabletten	Prednisolon 20 mg	Aliud
7626056	PREDNISOLONE STADA 20 mg Tabletten	Prednisolon 20 mg	Stadapharm

7626079	PREDNISOLONE STADA 20 mg Tabletten	Prednisolon 20 mg	Stadapharm
7626085	PREDNISOLONE STADA 20 mg Tabletten	Prednisolon 20 mg	Stadapharm
4862158	DECORTIN H 5 mg Tabletten	Prednisolon 5 mg	Merck Healthc.
14191222	DECORTIN H 5 mg Tabletten	Prednisolon 5 mg	Merck Healthc.
4862164	DECORTIN H 5 mg Tabletten	Prednisolon 5 mg	Merck Healthc.
263047	DECORTIN H 5 mg Tabletten	Prednisolon 5 mg	Merck Healthc.
913628	DERMOSOLON 5 mg Tabletten	Prednisolon 5 mg	Dermapharm
4969206	PREDNI H Tablinen 5 mg Tabletten	Prednisolon 5 mg	Zentiva Pharma
4969212	PREDNI H Tablinen 5 mg Tabletten	Prednisolon 5 mg	Zentiva Pharma
831675	PREDNI H Tablinen 5 mg Tabletten	Prednisolon 5 mg	Zentiva Pharma
745817	PREDNISOLONE 5 mg GALEN Tabletten	Prednisolon 5 mg	Galenpharma
15629502	PREDNISOLONE 5 mg GALEN Tabletten	Prednisolon 5 mg	Galenpharma
745823	PREDNISOLONE 5 mg GALEN Tabletten	Prednisolon 5 mg	Galenpharma
745846	PREDNISOLONE 5 mg GALEN Tabletten	Prednisolon 5 mg	Galenpharma
4022406	PREDNISOLONE 5 mg Jenapharm Tabletten	Prednisolon 5 mg	Mibe
6343014	PREDNISOLONE 5 mg Jenapharm Tabletten	Prednisolon 5 mg	Mibe
4022412	PREDNISOLONE 5 mg Jenapharm Tabletten	Prednisolon 5 mg	Mibe
1300402	PREDNISOLONE ACIS 5 mg Tabletten	Prednisolon 5 mg	Acis
1300419	PREDNISOLONE ACIS 5 mg Tabletten	Prednisolon 5 mg	Acis
1300425	PREDNISOLONE ACIS 5 mg Tabletten	Prednisolon 5 mg	Acis
4208335	PREDNISOLONE AL 5 mg Tabletten	Prednisolon 5 mg	Aliud
3792622	PREDNISOLONE AL 5 mg Tabletten	Prednisolon 5 mg	Aliud
4208341	PREDNISOLONE AL 5 mg Tabletten	Prednisolon 5 mg	Aliud
4208358	PREDNISOLONE AL 5 mg Tabletten	Prednisolon 5 mg	Aliud
7625849	PREDNISOLONE STADA 5 mg Tabletten	Prednisolon 5 mg	Stadapharm
17827815	PREDNISOLONE STADA 5 mg Tabletten	Prednisolon 5 mg	Stadapharm
7625973	PREDNISOLONE STADA 5 mg Tabletten	Prednisolon 5 mg	Stadapharm
263107	DECORTIN H 50 mg Tabletten	Prednisolon 50 mg	Merck Healthc.
14191274	DECORTIN H 50 mg Tabletten	Prednisolon 50 mg	Merck Healthc.
263113	DECORTIN H 50 mg Tabletten	Prednisolon 50 mg	Merck Healthc.
4547675	PREDNI H Tablinen 50 mg Tabletten	Prednisolon 50 mg	Zentiva Pharma

2719617	PREDNI H Tablinen 50 mg Tabletten	Prednisolon 50 mg	Zentiva Pharma
2561866	PREDNIHEXAL 50 mg Tabletten	Prednisolon 50 mg	Hexal
1484603	PREDNISOLONE 50 mg GALEN Tabletten	Prednisolon 50 mg	Galenpharma
1484632	PREDNISOLONE 50 mg GALEN Tabletten	Prednisolon 50 mg	Galenpharma
235861	PREDNISOLONE 50 mg Jenapharm Tabletten	Prednisolon 50 mg	Mibe
14028930	PREDNISOLONE 50 mg Jenapharm Tabletten	Prednisolon 50 mg	Mibe
235878	PREDNISOLONE 50 mg Jenapharm Tabletten	Prednisolon 50 mg	Mibe
985177	PREDNISOLONE ACIS 50 mg Tabletten	Prednisolon 50 mg	Acis
985183	PREDNISOLONE ACIS 50 mg Tabletten	Prednisolon 50 mg	Acis
4216168	PREDNISOLONE AL 50 mg Tabletten	Prednisolon 50 mg	Aliud
4216180	PREDNISOLONE AL 50 mg Tabletten	Prednisolon 50 mg	Aliud
7626116	PREDNISOLONE STADA 50 mg Tabletten	Prednisolon 50 mg	Stadapharm
7626139	PREDNISOLONE STADA 50 mg Tabletten	Prednisolon 50 mg	Stadapharm
11518958	PREDNISONE 10 mg GALEN Tabletten	Prednison 10 mg	Galenpharma
11518964	PREDNISONE 10 mg GALEN Tabletten	Prednison 10 mg	Galenpharma
11518970	PREDNISONE 10 mg GALEN Tabletten	Prednison 10 mg	Galenpharma
4689872	DECORTIN 20 mg Tabletten	Prednison 20 mg	Merck Healthc.
14191191	DECORTIN 20 mg Tabletten	Prednison 20 mg	Merck Healthc.
178347	DECORTIN 20 mg Tabletten	Prednison 20 mg	Merck Healthc.
4862112	DECORTIN 20 mg Tabletten	Prednison 20 mg	Merck Healthc.
4689895	DECORTIN 20 mg Tabletten	Prednison 20 mg	Merck Healthc.
4689903	DECORTIN 20 mg Tabletten	Prednison 20 mg	Merck Healthc.
1484649	PREDNISONE 20 mg GALEN Tabletten	Prednison 20 mg	Galenpharma
1484655	PREDNISONE 20 mg GALEN Tabletten	Prednison 20 mg	Galenpharma
1484661	PREDNISONE 20 mg GALEN Tabletten	Prednison 20 mg	Galenpharma
1484678	PREDNISONE 20 mg GALEN Tabletten	Prednison 20 mg	Galenpharma
2231620	PREDNISONE acis 20 mg Tabletten	Prednison 20 mg	Acis
2173437	PREDNISONE acis 20 mg Tabletten	Prednison 20 mg	Acis
2173443	PREDNISONE acis 20 mg Tabletten	Prednison 20 mg	Acis
2173466	PREDNISONE acis 20 mg Tabletten	Prednison 20 mg	Acis
2461142	PREDNISONE HEXAL 20 mg Tabletten	Prednison 20 mg	Hexal

2461159	PREDNISONE HEXAL 20 mg Tabletten	Prednison 20 mg	Hexal
2461165	PREDNISONE HEXAL 20 mg Tabletten	Prednison 20 mg	Hexal
4862081	DECORTIN 5 mg Tabletten	Prednison 5 mg	Merck Healthc.
14191185	DECORTIN 5 mg Tabletten	Prednison 5 mg	Merck Healthc.
4862098	DECORTIN 5 mg Tabletten	Prednison 5 mg	Merck Healthc.
262763	DECORTIN 5 mg Tabletten	Prednison 5 mg	Merck Healthc.
6315070	DECORTIN 5 mg Tabletten	Prednison 5 mg	Merck Healthc.
745875	PREDNISONE 5 mg GALEN Tabletten	Prednison 5 mg	Galenpharma
745881	PREDNISONE 5 mg GALEN Tabletten	Prednison 5 mg	Galenpharma
745898	PREDNISONE 5 mg GALEN Tabletten	Prednison 5 mg	Galenpharma
1998472	PREDNISONE acis 5 mg Tabletten	Prednison 5 mg	Acis
1998489	PREDNISONE acis 5 mg Tabletten	Prednison 5 mg	Acis
1998495	PREDNISONE acis 5 mg Tabletten	Prednison 5 mg	Acis
2461107	PREDNISONE HEXAL 5 mg Tabletten	Prednison 5 mg	Hexal
2461113	PREDNISONE HEXAL 5 mg Tabletten	Prednison 5 mg	Hexal
2461136	PREDNISONE HEXAL 5 mg Tabletten	Prednison 5 mg	Hexal
262823	DECORTIN 50 mg Tabletten	Prednison 50 mg	Merck Healthc.
14191216	DECORTIN 50 mg Tabletten	Prednison 50 mg	Merck Healthc.
262846	DECORTIN 50 mg Tabletten	Prednison 50 mg	Merck Healthc.
1529139	PREDNISONE 50 mg GALEN Tabletten	Prednison 50 mg	Galenpharma
745869	PREDNISONE 50 mg GALEN Tabletten	Prednison 50 mg	Galenpharma
2173472	PREDNISONE acis 50 mg Tabletten	Prednison 50 mg	Acis
2173489	PREDNISONE acis 50 mg Tabletten	Prednison 50 mg	Acis
2561843	PREDNISONE HEXAL 50 mg Tabletten	Prednison 50 mg	Hexal
2461188	PREDNISONE HEXAL 50 mg Tabletten	Prednison 50 mg	Hexal
1476845	VOLON 16 mg Tabletten	Triamcinolon 16 mg	Dermapharm
1115774	VOLON 4 mg Tabletten	Triamcinolon 4 mg	Dermapharm
1115780	VOLON 4 mg Tabletten	Triamcinolon 4 mg	Dermapharm
7371627	VOLON 4 mg Tabletten	Triamcinolon 4 mg	Emra Med
7371633	VOLON 4 mg Tabletten	Triamcinolon 4 mg	Emra Med
3134770	VOLON 4 mg Tabletten	Triamcinolon 4 mg	Eurim

3267005	VOLON 4 mg Tabletten B	Triamcinolon 4 mg	Docpharm
3267011	VOLON 4 mg Tabletten B	Triamcinolon 4 mg	Docpharm
1115828	VOLON 8 mg Tabletten	Triamcinolon 8 mg	Dermapharm
1115834	VOLON 8 mg Tabletten	Triamcinolon 8 mg	Dermapharm
7371604	VOLON 8 mg Tabletten	Triamcinolon 8 mg	Emra Med
7371610	VOLON 8 mg Tabletten	Triamcinolon 8 mg	Emra Med
3293712	VOLON 8 mg Tabletten B	Triamcinolon 8 mg	Docpharm
3293729	VOLON 8 mg Tabletten B	Triamcinolon 8 mg	Docpharm

*PZN (Pharmazentralnummer)

Online-Tabelle 4 – PZN-Codes zur Identifizierung topischer Glukokortikoide

PZN	Artikelbezeichnung	Wirkstoff / Menge	Hersteller
3082665	AMCIDERM Creme	Amcinonid	Almirall Hermal
3276808	AMCIDERM Creme	Amcinonid	Almirall Hermal
3082659	AMCIDERM Creme	Amcinonid	Almirall Hermal
1697724	DIPROSONE Creme	Betamethasondipropionat	Organon Health.
1854276	DIPROSONE Creme	Betamethasondipropionat	Organon Health.
1854299	DIPROSONE Salbe	Betamethasondipropionat	Organon Health.
2777589	DIPROSIS Salbe	Betamethasondipropionat	Organon Health.
2777595	DIPROSIS Salbe	Betamethasondipropionat	Organon Health.
3007132	DIPROSIS Salbe	Betamethasondipropionat	Organon Health.
6160791	DIPROSONE Creme	Betamethasondipropionat	Organon Health.
6160816	DIPROSONE Salbe	Betamethasondipropionat	Organon Health.
120922	BETNESOL V 0,1% Lotion	Betamethasonvalerat	GlaxoSmithKl.
165215	SODERM Salbe	Betamethasonvalerat	Dermapharm
165221	SODERM Salbe	Betamethasonvalerat	Dermapharm
165238	SODERM Salbe	Betamethasonvalerat	Dermapharm
165244	SODERM Creme	Betamethasonvalerat	Dermapharm
165250	SODERM Creme	Betamethasonvalerat	Dermapharm
165267	SODERM Creme	Betamethasonvalerat	Dermapharm

165273	SODERM Lotion	Betamethasonvalerat	Dermapharm
165296	SODERM Lotion	Betamethasonvalerat	Dermapharm
165304	SODERM Lotion	Betamethasonvalerat	Dermapharm
236286	BEMON 0,1% Creme	Betamethasonvalerat	Esteve
236292	BEMON 0,1% Creme	Betamethasonvalerat	Esteve
236300	BEMON 0,1% Creme	Betamethasonvalerat	Esteve
1292447	SODERM Creme	Betamethasonvalerat	Dermapharm
1292453	SODERM Salbe	Betamethasonvalerat	Dermapharm
1356414	BETAGALEN 0.1% Lotion	Betamethasonvalerat	Galenpharma
1639738	BETNESOL V 0,1% Salbe	Betamethasonvalerat	GlaxoSmithKl.
1639744	BETNESOL V 0,1% Creme	Betamethasonvalerat	GlaxoSmithKl.
2339429	BETNESOL V 0,1% Lotion	Betamethasonvalerat	GlaxoSmithKl.
3280572	BETNESOL V 0,1% Lotion	Betamethasonvalerat	GlaxoSmithKl.
3675514	CORDES BETA Salbe	Betamethasonvalerat	Ichthyol-Ges.
3675543	CORDES BETA Salbe	Betamethasonvalerat	Ichthyol-Ges.
3898160	CORDES BETA Creme	Betamethasonvalerat	Ichthyol-Ges.
3898208	CORDES BETA Creme	Betamethasonvalerat	Ichthyol-Ges.
6180150	BETNESOL V 0,1% Salbe	Betamethasonvalerat	GlaxoSmithKl.
6880284	BETAGALEN 0,1% Creme	Betamethasonvalerat	Galenpharma
6880290	BETAGALEN 0,1% Creme	Betamethasonvalerat	Galenpharma

6880309	BETAGALEN 0,1% Creme	Betamethasonvalerat	Galenpharma
6880315	BETAGALEN 0,1% Salbe	Betamethasonvalerat	Galenpharma
6880321	BETAGALEN 0,1% Salbe	Betamethasonvalerat	Galenpharma
6880338	BETAGALEN 0,1% Salbe	Betamethasonvalerat	Galenpharma
6880344	BETAGALEN 0.1% Lotion	Betamethasonvalerat	Galenpharma
6880350	BETAGALEN 0.1% Lotion	Betamethasonvalerat	Galenpharma
6975381	BETA CREME Lichtenstein	Betamethasonvalerat	Zentiva Pharma
6975398	BETA CREME Lichtenstein	Betamethasonvalerat	Zentiva Pharma
6975412	BETA CREME Lichtenstein	Betamethasonvalerat	Zentiva Pharma
12378815	BETNESOL V 0,1% Creme	Betamethasonvalerat	GlaxoSmithKl.
14054092	SODERM Creme	Betamethasonvalerat	Dermapharm
14054117	SODERM Lotion	Betamethasonvalerat	Dermapharm
16912269	BETAGALEN 0,1% Salbe	Betamethasonvalerat	Galenpharma
16912275	BETAGALEN 0,1% Creme	Betamethasonvalerat	Galenpharma
16912306	BETAGALEN 0.1% Lotion	Betamethasonvalerat	Galenpharma
1911330	DERMOXIN Creme	Clobetasol-17-propionat	GlaxoSmithKl.
1911347	DERMOXIN Creme	Clobetasol-17-propionat	GlaxoSmithKl.
1911407	DERMOXIN Salbe	Clobetasol-17-propionat	GlaxoSmithKl.
1911413	DERMOXIN Salbe	Clobetasol-17-propionat	GlaxoSmithKl.
2339441	DERMOXINALE Lösung	Clobetasol-17-propionat	GlaxoSmithKl.

2662076	CLOBEGALEN 0,05% Creme	Clobetasol-17-propionat	Galenpharma
2662099	CLOBEGALEN 0,05% Creme	Clobetasol-17-propionat	Galenpharma
2662107	CLOBEGALEN 0,05% Creme	Clobetasol-17-propionat	Galenpharma
2662260	CLOBEGALEN Lotion 0,05% Emulsion z.Anw.auf d.Haut	Clobetasol-17-propionat	Galenpharma
2662277	CLOBEGALEN Lotion 0,05% Emulsion z.Anw.auf d.Haut	Clobetasol-17-propionat	Galenpharma
2662283	CLOBEGALEN Lotion 0,05% Emulsion z.Anw.auf d.Haut	Clobetasol-17-propionat	Galenpharma
2662308	CLOBEGALEN Lösung 0,05% z.Anw.auf d.Haut	Clobetasol-17-propionat	Galenpharma
2662314	CLOBEGALEN Lösung 0,05% z.Anw.auf d.Haut	Clobetasol-17-propionat	Galenpharma
2662320	CLOBEGALEN Lösung 0,05% z.Anw.auf d.Haut	Clobetasol-17-propionat	Galenpharma
3178721	BUTAVATE Lösung	Clobetasol-17-propionat	Eurim
4712424	BUTAVATE Lösung	Clobetasol-17-propionat	Emra Med
4881836	CLOBETASOL acis Salbe	Clobetasol-17-propionat	Acis
4881871	CLOBETASOL acis Salbe	Clobetasol-17-propionat	Acis
4881888	CLOBETASOL acis Salbe	Clobetasol-17-propionat	Acis
4882072	CLOBETASOL acis Fettsalbe	Clobetasol-17-propionat	Acis
4882149	CLOBETASOL acis Fettsalbe	Clobetasol-17-propionat	Acis
4882155	CLOBETASOL acis Fettsalbe	Clobetasol-17-propionat	Acis
4882161	CLOBETASOL acis Creme	Clobetasol-17-propionat	Acis
4882853	CLOBETASOL acis Creme	Clobetasol-17-propionat	Acis
4883404	CLOBETASOL acis Creme	Clobetasol-17-propionat	Acis

4883427	CLOBETASOL acis Crinale Lösung	Clobetasol-17-propionat	Acis
4884326	CLOBETASOL acis Crinale Lösung	Clobetasol-17-propionat	Acis
6978020	KARISON Crinale Lösung	Clobetasol-17-propionat	Dermapharm
6978037	KARISON Crinale Lösung	Clobetasol-17-propionat	Dermapharm
6978043	KARISON Crinale Lösung	Clobetasol-17-propionat	Dermapharm
6978066	KARISON Creme	Clobetasol-17-propionat	Dermapharm
6978072	KARISON Creme	Clobetasol-17-propionat	Dermapharm
6978089	KARISON Creme	Clobetasol-17-propionat	Dermapharm
6978095	KARISON Fettsalbe	Clobetasol-17-propionat	Dermapharm
6978103	KARISON Fettsalbe	Clobetasol-17-propionat	Dermapharm
6978126	KARISON Fettsalbe	Clobetasol-17-propionat	Dermapharm
6978132	KARISON Salbe	Clobetasol-17-propionat	Dermapharm
6978149	KARISON Salbe	Clobetasol-17-propionat	Dermapharm
6978155	KARISON Salbe	Clobetasol-17-propionat	Dermapharm
8753213	CLOBEGALEN 0,05% Salbe	Clobetasol-17-propionat	Galenpharma
8753236	CLOBEGALEN 0,05% Salbe	Clobetasol-17-propionat	Galenpharma
8753242	CLOBEGALEN 0,05% Salbe	Clobetasol-17-propionat	Galenpharma
9403981	DERMOVATE 0,5 mg/g Salbe	Clobetasol-17-propionat	Eurim
9403998	DERMOVATE 0,5 mg/g Salbe	Clobetasol-17-propionat	Eurim
14043668	KARISON Creme	Clobetasol-17-propionat	Dermapharm

14053980	KARISON Crinale Lösung	Clobetasol-17-propionat	Dermapharm
14053997	KARISON Fettsalbe	Clobetasol-17-propionat	Dermapharm
14054005	KARISON 0,5 mg/g Salbe	Clobetasol-17-propionat	Dermapharm
15398385	DERMOVATE 0,5 mg/g Salbe	Clobetasol-17-propionat	Emra Med
15398391	DERMOVATE 0,5 mg/g Salbe	Clobetasol-17-propionat	Emra Med
17922293	CLOBEGALEN 0,05% Salbe	Clobetasol-17-propionat	Galenpharma
17922301	CLOBEGALEN 0,05% Creme	Clobetasol-17-propionat	Galenpharma
17922318	CLOBEGALEN Lotion 0,05% Emulsion z.Anw.auf d.Haut	Clobetasol-17-propionat	Galenpharma
17922324	CLOBEGALEN Lösung 0,05% z.Anw.auf d.Haut	Clobetasol-17-propionat	Galenpharma
18226503	BUTAVATE 0,5 mg/g Lösung	Clobetasol-17-propionat	kohlpharma
18302670	CLOBEGALEN Lotion 0,05% Emulsion z.Anw.auf d.Haut	Clobetasol-17-propionat	Galenpharma
4097775	DEXAMETHASON Creme	Dexamethason	Abanta Pharma
4909291	DEXAMETHASON Creme	Dexamethason	Abanta Pharma
4909316	DEXAMETHASON Creme	Dexamethason	Abanta Pharma
247315	NERISONE Creme	Diflucortolon-21-valerat	kohlpharma
247338	NERISONE Creme	Diflucortolon-21-valerat	kohlpharma
247545	NERISONE Salbe	Diflucortolon-21-valerat	kohlpharma
247893	NERISONE Salbe	Diflucortolon-21-valerat	kohlpharma
1692661	NERISONA 0,1% Salbe	Diflucortolon-21-valerat	Leo Pharma
1692721	NERISONA 0,1% Creme	Diflucortolon-21-valerat	Leo Pharma

4963505	NERISONA Fettsalbe	Diflucortolon-21-valerat	kohlpharma
4963511	NERISONA Fettsalbe	Diflucortolon-21-valerat	kohlpharma
7548273	NERISONE Creme	Diflucortolon-21-valerat	Emra Med
7548296	NERISONE Creme	Diflucortolon-21-valerat	Emra Med
8595352	NERISONA Salbe	Diflucortolon-21-valerat	Emra Med
8595369	NERISONA Salbe	Diflucortolon-21-valerat	Emra Med
8595381	NERISONA Fettsalbe	Diflucortolon-21-valerat	Emra Med
8595398	NERISONA Fettsalbe	Diflucortolon-21-valerat	Emra Med
17875369	NERISONA 0,1% Creme	Diflucortolon-21-valerat	Eurim
18754946	NERISONA 0,1% Salbe	Diflucortolon-21-valerat	Eurim
18754952	NERISONA 0,1% Salbe	Diflucortolon-21-valerat	Eurim
344343	JELLIN Creme	Fluocinolonacetonid	Teofarma
344389	JELLIN Salbe	Fluocinolonacetonid	Teofarma
3654831	JELLIN Creme	Fluocinolonacetonid	Teofarma
3654848	JELLIN Creme	Fluocinolonacetonid	Teofarma
3654860	JELLIN Salbe	Fluocinolonacetonid	Teofarma
3654877	JELLIN Salbe	Fluocinolonacetonid	Teofarma
4010969	FLUCINAR 0,025% Creme	Fluocinolonacetonid	Abanta Pharma
4010975	FLUCINAR Salbe 0,025%	Fluocinolonacetonid	Abanta Pharma
4674758	FLUCINAR 0,025% Creme	Fluocinolonacetonid	Abanta Pharma

4674764	FLUCINAR 0,025% Salbe	Fluocinolonacetonid	Abanta Pharma
6320177	FLUCINAR 0,025% Salbe	Fluocinolonacetonid	Abanta Pharma
9264077	FLUCINAR 0,025% Creme	Fluocinolonacetonid	Abanta Pharma
3122100	TOPSYM Salbe	Fluocinonid	Teofarma
3182131	TOPSYM F Salbe	Fluocinonid	Teofarma
3196328	TOPSYM Creme	Fluocinonid	Teofarma
3196334	TOPSYM Creme	Fluocinonid	Teofarma
3799423	TOPSYM Lösung	Fluocinonid	Teofarma
10730283	TOPSYM Lösung	Fluocinonid	Teofarma
4950773	FLUTIVATE Creme	Fluticason-17-propionat	GlaxoSmithKl.
278014	LATICORT 1% Creme	Hydrocortison	Almirall Hermal
278132	LATICORT 1% Creme	Hydrocortison	Almirall Hermal
587749	HYDROCUTAN 1% Salbe	Hydrocortison	Dermapharm
587755	HYDROCUTAN 1% Salbe	Hydrocortison	Dermapharm
587761	HYDROCUTAN 1% Salbe	Hydrocortison	Dermapharm
694801	SYSTRAL HYDROCORT Emulsion	Hydrocortison	Cooper Consumer
694818	SYSTRAL HYDROCORT Emulsion	Hydrocortison	Cooper Consumer
1138717	HYDROCUTAN 0.25% Creme	Hydrocortison	Dermapharm
1138723	HYDROCUTAN 0.25% Creme	Hydrocortison	Dermapharm
1234065	SYSTRAL HYDROCORT 0.5% Creme	Hydrocortison	Cooper Consumer

1356466	HYDROGALEN LOTION Emulsion	Hydrocortison	Galenpharma
1884751	LATICORT 1% Creme	Hydrocortison	Almirall Hermal
1995025	ALFASON Creme	Hydrocortison	Cheplapharm
1995083	ALFASON Salbe	Hydrocortison	Cheplapharm
2094761	ALFASON Creme	Hydrocortison	Cheplapharm
2094778	ALFASON Salbe	Hydrocortison	Cheplapharm
2138990	LINOLA AKUT 0.5% Creme	Hydrocortison	Wolff Bielefeld
2387486	ALFASON CRESA Creme	Hydrocortison	Cheplapharm
2387492	ALFASON CRESA Creme	Hydrocortison	Cheplapharm
2833158	LATICORT 1% Creme	Hydrocortison	Almirall Hermal
3424232	HYDROCORTISON HEUMANN 1% Creme	Hydrocortison	Heumann
3424249	HYDROCORTISON HEUMANN 1% Creme	Hydrocortison	Heumann
3482212	SANATISON MONO 0,33% Creme	Hydrocortison	Robugen
3482229	SANATISON MONO 0,33% Creme	Hydrocortison	Robugen
3482235	SANATISON MONO 0,33% Creme	Hydrocortison	Robugen
3482241	SANATISON MONO 1% Creme	Hydrocortison	Robugen
3482258	SANATISON MONO 1% Creme	Hydrocortison	Robugen
3482264	SANATISON MONO 1% Creme	Hydrocortison	Robugen
4405521	LINOLACORT HYDRO 1.0 Creme	Hydrocortison	Wolff Bielefeld
4405538	LINOLACORT HYDRO 1.0 Creme	Hydrocortison	Wolff Bielefeld

4405544	LINOLACORT HYDRO 1.0 Creme	Hydrocortison	Wolff Bielefeld
4465121	SOVENTOL HYDROCORT 0.5% Creme	Hydrocortison	Medice
4465138	SOVENTOL HYDROCORT 0.5% Creme	Hydrocortison	Medice
4468705	EBENOL 1% Creme	Hydrocortison	Strathmann
4468728	EBENOL 1% Creme	Hydrocortison	Strathmann
4479146	EBENOL 0.25% Creme	Hydrocortison	Strathmann
4479152	EBENOL 0.25% Creme	Hydrocortison	Strathmann
4613509	ALFASON CRELO Emulsion	Hydrocortison	Cheplapharm
4782347	ALFASON Creme	Hydrocortison	Cheplapharm
4782353	ALFASON CRESA Creme	Hydrocortison	Cheplapharm
4829787	ALFASON CRELO Emulsion	Hydrocortison	Cheplapharm
5103302	EBENOL 0.5% Creme	Hydrocortison	Strathmann
5103319	EBENOL 0.5% Creme	Hydrocortison	Strathmann
5994755	LINOLA AKUT 0.5% Creme	Hydrocortison	Wolff Bielefeld
6576818	HYDROCUTAN 0.5% Creme	Hydrocortison	Dermapharm
6577025	HYDROCUTAN 1% Creme	Hydrocortison	Dermapharm
6577031	HYDROCUTAN 1% Creme	Hydrocortison	Dermapharm
6577048	HYDROCUTAN 1% Creme	Hydrocortison	Dermapharm
6836981	EBENOL HCA 0.25% Creme	Hydrocortison	Strathmann
7238495	SYSTRAL HYDROCORT 0.5% Creme	Hydrocortison	Cooper Consumer

7276082	MUNI 0.5% CremeHC	Hydrocortison	Robugen
7276113	MUNI 0.5% CremeHC	Hydrocortison	Robugen
8604393	HYDROGALEN Salbe	Hydrocortison	Galenpharma
8604401	HYDROGALEN Salbe	Hydrocortison	Galenpharma
8604418	HYDROGALEN Salbe	Hydrocortison	Galenpharma
8604424	HYDROGALEN Creme	Hydrocortison	Galenpharma
8604430	HYDROGALEN Creme	Hydrocortison	Galenpharma
8604447	HYDROGALEN Creme	Hydrocortison	Galenpharma
8604453	HYDROGALEN LOTION Emulsion	Hydrocortison	Galenpharma
9087474	HYDROGALEN LOTION Emulsion	Hydrocortison	Galenpharma
9703298	HYDROCORTISON 0.5% Creme	Hydrocortison	Ratiopharm
9703312	HYDROCORTISON 0.5% Creme	Hydrocortison	Ratiopharm
10215256	POSTERICORT Salbe	Hydrocortison	Kade
10215262	POSTERICORT Salbe	Hydrocortison	Kade
10215279	POSTERICORT Salbe	Hydrocortison	Kade
10215285	POSTERICORT Salbe	Hydrocortison	Kade
10714350	SOVENTOL HYDROCORTISONACETAT 0.5% Creme	Hydrocortison	Medice
10714367	SOVENTOL HYDROCORTISONACETAT 0,5% Creme	Hydrocortison	Medice
10714373	SOVENTOL HYDROCORTISONACETAT 0.25% Creme	Hydrocortison	Medice
10714396	SOVENTOL HYDROCORTISONACETAT 0.25% Creme	Hydrocortison	Medice

10714404	SOVENTOL HYDROCORTISONACETAT 0.25% Creme	Hydrocortison	Medice
10786214	CORTILIND HYDROCORTISON Creme	Hydrocortison	Stada
10796968	FENIHYDROCORT 0,5% Creme	Hydrocortison	Haleon
10796974	FENIHYDROCORT 0,5% Creme	Hydrocortison	Haleon
10796980	FENIHYDROCORT 0.25% Creme	Hydrocortison	Haleon
10796997	FENIHYDROCORT 0.25% Creme	Hydrocortison	Haleon
14214489	HYDROCORTISON 1% Creme	Hydrocortison	Gsk Otc
14236841	HYDROCORT 0,25 % Creme	Hydrocortison	1A Pharma
14236864	HYDROCORT 0,5 % Creme	Hydrocortison	1A Pharma
14236893	HYDROCORT 1 % Creme	Hydrocortison	1A Pharma
14236901	HYDROCORT 1 % Creme	Hydrocortison	1A Pharma
14236918	HYDROCORT 1 % Creme	Hydrocortison	1A Pharma
14372260	HYDROCORTISON AL 0,5 % Creme	Hydrocortison	Aliud Pharma
14372283	HYDROCORTISON AL 0,5 % Creme	Hydrocortison	Aliud Pharma
16663783	HYDROGALEN AKUT Creme	Hydrocortison	Galenpharma
16663808	HYDROGALEN AKUT Creme	Hydrocortison	Galenpharma
16808483	HYDROCORTISON STADA Creme	Hydrocortison	Stada
17231577	MUNI HC 0,5% Creme	Hydrocortison	Robugen
18371903	LOCOID CRELO 0,1 % Emulsion	Hydrocortison	Eurim
19067610	LOCOID CRELO 0.1% Emulsion	Hydrocortison	Kohlpharma

259407	ADVANTAN Milch 0,1% Emulsion z.Anwend.a.d.Haut	Methylprednisolonaceponat	Leo Pharma
259413	ADVANTAN Milch 0,1% Emulsion z.Anwend.a.d.Haut	Methylprednisolonaceponat	Leo Pharma
3113905	ADVANTAN 0,1% Creme	Methylprednisolonaceponat	Leo Pharma
3113911	ADVANTAN 0,1% Salbe	Methylprednisolonaceponat	Leo Pharma
3113928	ADVANTAN 0,1% Fettsalbe	Methylprednisolonaceponat	Leo Pharma
3113934	ADVANTAN Milch 0,1% Emulsion z.Anwend.a.d.Haut	Methylprednisolonaceponat	Leo Pharma
4939240	ADVANTAN 0,1% Creme	Methylprednisolonaceponat	Leo Pharma
4939257	ADVANTAN 0,1% Creme	Methylprednisolonaceponat	Leo Pharma
4939263	ADVANTAN 0,1% Creme	Methylprednisolonaceponat	Leo Pharma
4939292	ADVANTAN 0,1% Salbe	Methylprednisolonaceponat	Leo Pharma
4939300	ADVANTAN 0,1% Salbe	Methylprednisolonaceponat	Leo Pharma
4939317	ADVANTAN 0,1% Salbe	Methylprednisolonaceponat	Leo Pharma
4939352	ADVANTAN 0,1% Fettsalbe	Methylprednisolonaceponat	Leo Pharma
4939369	ADVANTAN 0,1% Fettsalbe	Methylprednisolonaceponat	Leo Pharma
13902425	ADVANTAN 0,1% Creme	Methylprednisolonaceponat	Leo Pharma
13902431	ADVANTAN 0,1% Fettsalbe	Methylprednisolonaceponat	Leo Pharma
13902448	ADVANTAN Milch 0,1% Emulsion z.Anwend.a.d.Haut	Methylprednisolonaceponat	Leo Pharma
13902454	ADVANTAN 0,1% Salbe	Methylprednisolonaceponat	Leo Pharma
16597603	METIGALEN Lotion 1 mg/g Emulsion z.Anw.auf d.Haut	Methylprednisolonaceponat	Galenpharma
16597626	METIGALEN Lotion 1 mg/g Emulsion z.Anw.auf d.Haut	Methylprednisolonaceponat	Galenpharma

16597632	METIGALEN Lotion 1 mg/g Emulsion z.Anw.auf d.Haut	Methylprednisolonaceponat	Galenpharma
16597649	METIGALEN Lotion 1 mg/g Emulsion z.Anw.auf d.Haut	Methylprednisolonaceponat	Galenpharma
4004213	ECURAL MINI 1 mg/g Salbe	Mometasonfuroat	Organon Health.
4578291	ECURAL Fettcreme	Mometasonfuroat	Organon Health.
4578316	ECURAL Fettcreme	Mometasonfuroat	Organon Health.
4578345	ECURAL Salbe	Mometasonfuroat	Organon Health.
4578351	ECURAL Salbe	Mometasonfuroat	Organon Health.
5025602	MONOVO 1 mg/g Creme	Mometasonfuroat	Almirall Hermal
5025619	MONOVO 1 mg/g Creme	Mometasonfuroat	Almirall Hermal
5025654	MONOVO 1 mg/g Creme	Mometasonfuroat	Almirall Hermal
6101699	MOMEGALEN 1 mg/g Salbe	Mometasonfuroat	Galenpharma
6101713	MOMEGALEN 1 mg/g Salbe	Mometasonfuroat	Galenpharma
6866918	ECURAL Fettcreme	Mometasonfuroat	Organon Health.
6866924	ECURAL Salbe	Mometasonfuroat	Organon Health.
7517031	ELOCON Fettcreme	Mometasonfuroat	kohlpharma
7517048	ELOCON Fettcreme	Mometasonfuroat	kohlpharma
7517054	ELOCON Fettcreme	Mometasonfuroat	kohlpharma
8820441	MOMETASON Glenmark 1 mg/g Salbe	Mometasonfuroat	Glenmark
8820458	MOMETASON Glenmark 1 mg/g Salbe	Mometasonfuroat	Glenmark
8820464	MOMETASON Glenmark 1 mg/g Salbe	Mometasonfuroat	Glenmark

8820470	MOMETASON Glenmark 1 mg/g Salbe	Mometasonfuroat	Glenmark
9087497	MOMEGALEN 1 mg/g Salbe	Mometasonfuroat	Galenpharma
9087505	MOMEGALEN 1 mg/g Salbe	Mometasonfuroat	Galenpharma
9276152	ELOCOM Salbe	Mometasonfuroat	Emra Med
9276169	ELOCOM Salbe	Mometasonfuroat	Emra Med
9516969	MOMETASONFUROAT Glenmark 1 mg/g Creme	Mometasonfuroat	Glenmark
9516975	MOMETASONFUROAT Glenmark 1 mg/g Creme	Mometasonfuroat	Glenmark
9516981	MOMETASONFUROAT Glenmark 1 mg/g Creme	Mometasonfuroat	Glenmark
9942620	ECURAL MINI 1 mg/g Fettcreme Creme	Mometasonfuroat	Organon Health.
10115968	MOMECUTAN 1 mg/g Fettcreme	Mometasonfuroat	Dermapharm
10115974	MOMECUTAN 1 mg/g Fettcreme	Mometasonfuroat	Dermapharm
10116011	MOMECUTAN 1 mg/g Fettcreme	Mometasonfuroat	Dermapharm
10116028	MOMECUTAN 1 mg/g Fettcreme	Mometasonfuroat	Dermapharm
10116040	MOMECUTAN 1 mg/g Salbe	Mometasonfuroat	Dermapharm
10116057	MOMECUTAN 1 mg/g Salbe	Mometasonfuroat	Dermapharm
10116063	MOMECUTAN 1 mg/g Salbe	Mometasonfuroat	Dermapharm
10744747	MONOVO 1 mg/g Creme	Mometasonfuroat	Almirall Hermal
10760108	MOMETAHEXAL Derm 1 mg/g Creme	Mometasonfuroat	Hexal
10760114	MOMETAHEXAL Derm 1 mg/g Creme	Mometasonfuroat	Hexal
11605947	MOMEGALEN Fett 1 mg/g Creme	Mometasonfuroat	Galenpharma

11605953	MOMEGALEN Fett 1 mg/g Creme	Mometasonfuroat	Galenpharma
11605976	MOMEGALEN Fett 1 mg/g Creme	Mometasonfuroat	Galenpharma
11605982	MOMEGALEN Fett 1 mg/g Creme	Mometasonfuroat	Galenpharma
11605999	MOMEGALEN 1 mg/g Salbe	Mometasonfuroat	Galenpharma
13597933	MOMEGALEN 1 mg/g Creme	Mometasonfuroat	Galenpharma
13597956	MOMEGALEN 1 mg/g Creme	Mometasonfuroat	Galenpharma
13597985	MOMEGALEN 1 mg/g Creme	Mometasonfuroat	Galenpharma
13597991	MOMEGALEN 1 mg/g Creme	Mometasonfuroat	Galenpharma
14054011	MOMECUTAN 1 mg/g Fettcreme	Mometasonfuroat	Dermapharm
15878300	MOMECUTAN 1 mg/g Salbe	Mometasonfuroat	Dermapharm
16229673	MOMEGALEN 1 mg/g Creme	Mometasonfuroat	Galenpharma
16232942	MOMEGALEN 1 mg/g Salbe	Mometasonfuroat	Galenpharma
16232959	MOMEGALEN Fett 1 mg/g Creme	Mometasonfuroat	Galenpharma
18317499	ELOCOM Fettcreme	Mometasonfuroat	kohlpharma
19103748	ELOCOM 1 mg/g Fettcreme	Mometasonfuroat	Eurim
19103760	ELOCOM 1 mg/g Fettcreme	Mometasonfuroat	Eurim
19103777	ELOCOM 1 mg/g Fettcreme	Mometasonfuroat	Eurim
19103783	ELOCOM 1 mg/g Fettcreme	Mometasonfuroat	Eurim
2587966	PREDNITOP Creme	Prednicarbat	Dermapharm
2588003	PREDNITOP Creme	Prednicarbat	Dermapharm

2686852	PREDNITOP Creme	Prednicarbat	Dermapharm
2686869	PREDNITOP Creme	Prednicarbat	Dermapharm
2686875	PREDNITOP Fettsalbe	Prednicarbat	Dermapharm
2686881	PREDNITOP Fettsalbe	Prednicarbat	Dermapharm
2686898	PREDNITOP Fettsalbe	Prednicarbat	Dermapharm
2686906	PREDNITOP Fettsalbe	Prednicarbat	Dermapharm
2686912	PREDNITOP Salbe	Prednicarbat	Dermapharm
2686929	PREDNITOP Salbe	Prednicarbat	Dermapharm
2687142	PREDNITOP Salbe	Prednicarbat	Dermapharm
2688012	PREDNITOP Salbe	Prednicarbat	Dermapharm
3112892	DERMATOP Salbe	Prednicarbat	Fidia Pharma
3112900	DERMATOP Salbe	Prednicarbat	Fidia Pharma
3112917	DERMATOP Salbe	Prednicarbat	Fidia Pharma
3112923	DERMATOP Salbe	Prednicarbat	Fidia Pharma
3112952	DERMATOP Creme	Prednicarbat	Fidia Pharma
3112969	DERMATOP Creme	Prednicarbat	Fidia Pharma
3112975	DERMATOP Creme	Prednicarbat	Fidia Pharma
3112981	DERMATOP Creme	Prednicarbat	Fidia Pharma
3113012	DERMATOP Fettsalbe	Prednicarbat	Fidia Pharma
3113029	DERMATOP Fettsalbe	Prednicarbat	Fidia Pharma

3344473	DERMATOP Fettsalbe	Prednicarbat	Fidia Pharma
4881500	PREDNICARBAT acis Creme	Prednicarbat	Acis
4881517	PREDNICARBAT acis Creme	Prednicarbat	Acis
4881807	PREDNICARBAT acis Creme	Prednicarbat	Acis
4884332	PREDNICARBAT acis Salbe	Prednicarbat	Acis
4884450	PREDNICARBAT acis Salbe	Prednicarbat	Acis
4884467	PREDNICARBAT acis Salbe	Prednicarbat	Acis
4884473	PREDNICARBAT acis Salbe	Prednicarbat	Acis
4884823	PREDNICARBAT acis Fettsalbe	Prednicarbat	Acis
4885171	PREDNICARBAT acis Fettsalbe	Prednicarbat	Acis
4885188	PREDNICARBAT acis Fettsalbe	Prednicarbat	Acis
4885231	PREDNICARBAT acis Fettsalbe	Prednicarbat	Acis
4887520	PREDNICARBAT acis Creme	Prednicarbat	Acis
14053218	PREDNICARBAT acis Salbe	Prednicarbat	Acis
14054040	PREDNITOP Creme	Prednicarbat	Dermapharm
14054063	PREDNITOP Fettsalbe	Prednicarbat	Dermapharm
14054086	PREDNITOP Salbe	Prednicarbat	Dermapharm
15629525	PREDNICARBGALEN Fett 2,5 mg/g Creme	Prednicarbat	Galenpharma
15629554	PREDNICARBGALEN Fett 2,5 mg/g Creme	Prednicarbat	Galenpharma
15629560	PREDNICARBGALEN Fett 2,5 mg/g Creme	Prednicarbat	Galenpharma

15629577	PREDNICARBGALEN 2,5 mg/g Salbe	Prednicarbat	Galenpharma
15629583	PREDNICARBGALEN 2,5 mg/g Salbe	Prednicarbat	Galenpharma
15629608	PREDNICARBGALEN 2,5 mg/g Salbe	Prednicarbat	Galenpharma
15629614	PREDNICARBGALEN 2,5 mg/g Creme	Prednicarbat	Galenpharma
15629620	PREDNICARBGALEN 2,5 mg/g Creme	Prednicarbat	Galenpharma
15629637	PREDNICARBGALEN 2,5 mg/g Creme	Prednicarbat	Galenpharma
15634928	PREDNICARBGALEN Fett 2,5 mg/g Creme	Prednicarbat	Galenpharma
15634934	PREDNICARBGALEN 2,5 mg/g Salbe	Prednicarbat	Galenpharma
15634940	PREDNICARBGALEN 2,5 mg/g Creme	Prednicarbat	Galenpharma

*PZN (Pharmazentralnummer)

Online-Tabelle 5 – Übersicht der diagnostizierenden Fachärzt*innen

			≥ 12 Jahre			≥ 18 Jahre		
Art der Diagnose	Fachrichtungs-code	Fachrichtung	Anzahl der Personen, DADB	Anzahl der Personen, GKV-adjustiert [95 %-KI]	Anteil der inzidenten Personen mit Vitiligo-Diagnose, GKV-adjustiert	Anzahl der Personen, DADB	Anzahl der Personen, GKV-adjustiert [95 %-KI]	Anteil der inzidenten Personen mit Vitiligo-Diagnose, GKV-adjustiert
Gesicherte ambulante Diagnose	1	Allgemeinmedizin, Fachärzt*in	310	7.656 [6.849–8.580]	36,26 %	301	7.441 [6.645–8.354]	37,07 %
	2	Allgemeinmedizin, Hausärzt*in	27	659 [453–967]	3,16 %	26	635 [434–939]	3,20 %
	3	Innere Medizin, Hausärzt*in	164	4.063 [3.486–4.748]	19,18 %	161	3.991 [3.420–4.670]	19,83 %
	4	Anästhesiologie	< 10	-	-	< 10	-	-
	5	Augenheilkunde	< 10	-	-	< 10	-	-
	6	Chirurgie	< 10	-	-	< 10	-	-
	8	Viszeralchirurgie	< 10	-	-	< 10	-	-
	10	Orthopädie	< 10	-	-	< 10	-	-
	15	Frauenheilkunde und Geburtshilfe	39	1.039 [761–1.437]	4,56 %	39	1.039 [761–1.437]	4,80 %
	19	Hals-Nasen-Ohren-Heilkunde	< 10	-	-	< 10	-	-
	21	Haut- und Geschlechtskrankheiten	336	8.466 [7.604–9.441]	39,30 %	325	8.203 [7.352–9.163]	40,02 %
	23	Innere Medizin	< 10	-	-	< 10	-	-
	25	Endokrinologie und Diabetologie	< 10	-	-	< 10	-	-
	26	Gastroenterologie	< 10	-	-	< 10	-	-
	27	Hämatologie und Onkologie	< 10	-	-	< 10	-	-
	28	Kardiologie	< 10	-	-	< 10	-	-
	29	Nephrologie	< 10	-	-	< 10	-	-
	30	Pneumologie	< 10	-	-	< 10	-	-

	31	Rheumatologie	< 10	-	-	< 10	-	-
	34	Kinder- und Jugendmedizin, Hausärzt*in	15	360 [222–602]	1,75 %	n/a	n/a	n/a
	37	Neonatologie, Hausärzt*in	< 10	-	-	n/a	n/a	n/a
	40	Kinder- und Jugendmedizin, Fachärzt*in	< 10	-	-	n/a	n/a	n/a
	46	Kinder- und Jugendmedizin mit Schwerpunkt und Teilnahme an haus- und fachärztlicher Versorgung	< 10	-	-	n/a	n/a	n/a
	48	Laboratoriumsmedizin	< 10	-	-	< 10	-	-
	49	Mikrobiologie	< 10	-	-	< 10	-	-
	51	Nervenheilkunde (Neurologie und Psychiatrie)	< 10	-	-	< 10	-	-
	53	Neurologie	< 10	-	-	< 10	-	-
	54	Nuklearmedizin	< 10	-	-	< 10	-	-
	56	Pathologie	< 10	-	-	n/a	n/a	n/a
	57	Physikalissche und rehabilitative Medizin	< 10	-	-	< 10	-	-
	58	Psychiatrie und Psychotherapie	< 10	-	-	< 10	-	-
	61	Ärztliche Psychotherapie	< 10	-	-	< 10	-	-
	66	Transfusionmedizin	< 10	-	-	< 10	-	-
	67	Urologie	14	342 [205–582]	1,64 %	14	342 [205–582]	1,72 %
		Insgesamt – eindeutige Patient*innen	855	21.362 [19.969–22.871]		812	20.332 [18.972–21.809]	

Online-Tabelle 6 – Übersicht aller Begleiterkrankungen bei Vitiligo-Patient*innen in Deutschland im Jahr 2023

Begleiterkrankungen											
**ICD-10**	**Beschreibung**	**Anzahl der Patient*innen in der Prävalenzkohorte (DADB)**	**Anzahl der Patient*innen in der Prävalenzkohorte (GKV-adjustiert)**	**Anzahl der Patient*innen in der Kontrollkohorte (DADB)**	**Anzahl der Patient*innen in der Kontrollkohorte (GKV-adjustiert)**	**Anteil der Patient*innen in der Prävalenzkohorte (DADB)**	**Anteil der Patient*innen in der Kontrollkohorte (DADB)**	**Anteil der Patient*innen in der Prävalenzkohorte (GKV-adjustiert)**	**Anteil der Patient*innen in der Kontrollkohorte (GKV-adjustiert)**	**OR**	**p-Wert**
E31	Polyglanduläre Dysfunktion	26	621	9	211	0,70 %	0,02 %	0,67 %	0,02 %	27,79	< 0,001
L94	Sonstige lokalisierte Krankheiten des Bindegewebes	31	860	25	613	0,84 %	0,07 %	0,93 %	0,07 %	12,02	< 0,001
E06	Thyreoiditis	751	18.439	1.567	38.896	20,31 %	4,24 %	19,97 %	4,21 %	6,11	< 0,001
L63	Alopecia areata	53	1.275	91	2.271	1,43 %	0,25 %	1,38 %	0,25 %	5,59	< 0,001
E27	Sonstige Krankheiten der Nebenniere	25	611	47	1.224	0,68 %	0,13 %	0,66 %	0,13 %	5,01	< 0,001
E10	Diabetes mellitus, Typ 1	190	4.629	415	10.820	5,14 %	1,12 %	5,01 %	1,17 %	4,54	< 0,001
M34	Systemische Sklerose	16	450	36	960	0,43 %	0,10 %	0,49 %	0,10 %	4,30	< 0,001
E16	Sonstige Störungen der inneren Sekretion des Pankreas	29	697	69	1.868	0,78 %	0,19 %	0,76 %	0,20 %	4,01	< 0,001
M66	Spontanruptur der Synovialis und von Sehnen	11	275	27	672	0,30 %	0,07 %	0,30 %	0,07 %	3,89	< 0,001
D51	Vitamin-B12-Mangelanämie	86	2.254	232	6.536	2,33 %	0,63 %	2,44 %	0,71 %	3,68	< 0,001
D44	Neubildung unsicheren oder unbekannten	29	725	78	2.114	0,78 %	0,21 %	0,78 %	0,23 %	3,60	< 0,001

	Verhaltens der endokrinen Drüsen										
L93	Lupus erythematodes	18	446	51	1.300	0,49 %	0,14 %	0,48 %	0,14 %	3,46	< 0,001
B16	Akute Virushepatitis B	11	272	30	723	0,30 %	0,08 %	0,29 %	0,08 %	3,45	< 0,001
L56	Sonstige akute Hautveränderungen durch Ultraviolettstrahlen	37	985	104	2.616	1,00 %	0,28 %	1,07 %	0,28 %	3,42	< 0,001
M32	Systemischer Lupus erythematodes	11	279	32	798	0,30 %	0,09 %	0,30 %	0,09 %	3,33	< 0,001
D86	Sarkoidose	37	860	112	2.750	1,00 %	0,30 %	0,93 %	0,30 %	3,16	< 0,001
L90	Atrophische Hautkrankheiten	123	3.243	396	10.561	3,33 %	1,07 %	3,51 %	1,14 %	3,11	< 0,001
H06	Affektionen des Tränenapparates und der Orbita bei anderenorts klassifizierten Krankheiten	11	285	36	935	0,30 %	0,10 %	0,31 %	0,10 %	2,94	0,002
N11	Chronische tubulointerstitielle Nephritis	12	339	40	1.100	0,32 %	0,11 %	0,37 %	0,12 %	2,92	0,001
L43	Lichen ruber planus	25	623	85	2.282	0,68 %	0,23 %	0,68 %	0,25 %	2,89	< 0,001
L27	Dermatitis durch oral, enteral oder parenteral aufgenommene Substanzen	21	526	72	1.933	0,57 %	0,19 %	0,57 %	0,21 %	2,84	< 0,001
L81	Sonstige Störungen der Hautpigmentierung	174	4.356	606	15.570	4,71 %	1,64 %	4,72 %	1,69 %	2,83	< 0,001
E23	Unterfunktion und andere Störungen der Hypophyse	16	385	54	1.353	0,43 %	0,15 %	0,42 %	0,15 %	2,80	< 0,001

N72	Entzündliche Krankheit der Cervix uteri	13	315	46	1.134	0,35 %	0,12 %	0,34 %	0,12 %	2,75	0,001
N90	Sonstige nichtentzündliche Krankheiten der Vulva und des Perineums	75	2.004	279	7.544	2,03 %	0,75 %	2,17 %	0,82 %	2,71	< 0,001
L28	Lichen simplex chronicus und Prurigo	41	1.084	157	4.185	1,11 %	0,42 %	1,17 %	0,45 %	2,52	< 0,001
K90	Intestinale Malabsorption	44	1.075	172	4.407	1,19 %	0,47 %	1,16 %	0,48 %	2,50	< 0,001
A63	Sonstige vorwiegend durch Geschlechtsverkehr übertragene Krankheiten, anderenorts nicht klassifiziert	20	460	75	1.746	0,54 %	0,20 %	0,50 %	0,19 %	2,49	< 0,001
L50	Urtikaria	102	2.598	403	9.983	2,76 %	1,09 %	2,81 %	1,08 %	2,46	< 0,001
E80	Störungen des Porphyrin- und Bilirubinstoffwechsels	27	668	102	2.527	0,73 %	0,28 %	0,72 %	0,27 %	2,43	< 0,001
D43	Neubildung unsicheren oder unbekannten Verhaltens des Gehirns und des Zentralnervensystems	10	279	40	1.071	0,27 %	0,11 %	0,30 %	0,12 %	2,39	0,014
M07	Arthritis psoriatica und Arthritiden bei gastrointestinalen Grundkrankheiten	38	919	159	4.004	1,03 %	0,43 %	1,00 %	0,43 %	2,32	< 0,001

E74	Sonstige Störungen des Kohlenhydratstoffwechsels	55	1.370	228	5.762	1,49 %	0,62 %	1,48 %	0,62 %	2,31	< 0,001
F80	Umschriebene Entwicklungsstörungen des Sprechens und der Sprache	20	510	82	2.024	0,54 %	0,22 %	0,55 %	0,22 %	2,29	0,001
E03	Sonstige Hypothyreose	821	21.012	4.127	108.613	22,21 %	11,16 %	22,75 %	11,76 %	2,25	< 0,001
L01	Impetigo	15	378	64	1.593	0,41 %	0,17 %	0,41 %	0,17 %	2,25	0,005
E05	Hyperthyreose [Thyreotoxikose]	230	5.961	1.031	28.070	6,22 %	2,79 %	6,45 %	3,04 %	2,25	< 0,001
E07	Sonstige Krankheiten der Schilddrüse	234	5.952	1.057	28.066	6,33 %	2,86 %	6,44 %	3,04 %	2,25	< 0,001
Z01	Sonstige spezielle Untersuchungen und Abklärungen bei Personen ohne Beschwerden oder angegebene Diagnose	3.074	76.901	24.792	627.960	83,15 %	67,06 %	83,27 %	68,00 %	2,24	< 0,001
B98	Sonstige näher bezeichnete infektiöse Erreger als Ursache von Krankheiten, die in anderen Kapiteln klassifiziert sind	43	1.136	188	4.738	1,16 %	0,51 %	1,23 %	0,51 %	2,23	< 0,001
F95	Ticstörungen	22	529	94	2.402	0,60 %	0,25 %	0,57 %	0,26 %	2,23	< 0,001
N48	Sonstige Krankheiten des Penis	68	1.676	299	7.415	1,84 %	0,81 %	1,81 %	0,80 %	2,22	< 0,001
T73	Schäden durch sonstigen Mangel	11	256	47	1.112	0,30 %	0,13 %	0,28 %	0,12 %	2,21	0,019

L20	Atopisches [endogenes] Ekzem	266	6.582	1.196	29.583	7,20 %	3,24 %	7,13 %	3,20 %	2,20	< 0,001
E53	Mangel an sonstigen Vitaminen des Vitamin-B-Komplexes	185	5.124	840	23.442	5,00 %	2,27 %	5,55 %	2,54 %	2,20	< 0,001
N47	Vorhauthypertrophie, Phimose und Paraphimose	36	887	157	3.984	0,97 %	0,42 %	0,96 %	0,43 %	2,20	< 0,001
D18	Hämangiom und Lymphangiom	178	4.507	802	20.200	4,81 %	2,17 %	4,88 %	2,19 %	2,19	< 0,001
I86	Varizen sonstiger Lokalisationen	22	522	93	2.309	0,60 %	0,25 %	0,57 %	0,25 %	2,19	0,001
L65	Sonstiger Haarausfall ohne Narbenbildung	87	2.235	393	10.029	2,35 %	1,06 %	2,42 %	1,09 %	2,17	< 0,001
N46	Sterilität beim Mann	15	345	63	1.424	0,41 %	0,17 %	0,37 %	0,15 %	2,16	0,009
L84	Hühneraugen und Horn- (Haut-) Schwielen	39	977	177	4.666	1,05 %	0,48 %	1,06 %	0,51 %	2,11	< 0,001
R61	Hyperhidrose	66	1.675	305	7.801	1,79 %	0,82 %	1,81 %	0,84 %	2,11	< 0,001
Q44	Angeborene Fehlbildungen der Gallenblase, der Gallengänge und der Leber	20	517	93	2.624	0,54 %	0,25 %	0,56 %	0,28 %	2,09	0,003
D17	Gutartige Neubildung des Fettgewebes	107	2.640	497	12.244	2,89 %	1,34 %	2,86 %	1,33 %	2,09	< 0,001
J33	Nasenpolyp	62	1.477	289	7.159	1,68 %	0,78 %	1,60 %	0,78 %	2,07	< 0,001
B36	Sonstige oberflächliche Mykosen	85	2.237	397	10.193	2,30 %	1,07 %	2,42 %	1,10 %	2,06	< 0,001
U81	Gramnegative Erreger mit bestimmten	10	308	47	1.355	0,27 %	0,13 %	0,33 %	0,15 %	2,05	0,040

	Antibiotikaresistenzen, die besondere therapeutische oder hygienische Maßnahmen erfordern										
D70	Agranulozytose und Neutropenie	34	939	162	4.156	0,92 %	0,44 %	1,02 %	0,45 %	2,03	< 0,001
B35	Dermatophytose [Tinea]	242	6.373	1.187	30.437	6,55 %	3,21 %	6,90 %	3,30 %	2,03	< 0,001
L08	Sonstige lokale Infektionen der Haut und der Unterhaut	49	1.171	235	6.131	1,33 %	0,64 %	1,27 %	0,66 %	2,02	< 0,001
K58	Reizdarmsyndrom	92	2.320	442	11.279	2,49 %	1,20 %	2,51 %	1,22 %	2,01	< 0,001
L29	Pruritus	141	3.701	692	17.974	3,81 %	1,87 %	4,01 %	1,95 %	2,01	< 0,001
L40	Psoriasis	257	6.428	1.280	32.528	6,95 %	3,46 %	6,96 %	3,52 %	2,00	< 0,001
L91	Hypertrophe Hautkrankheiten	14	355	67	1.681	0,38 %	0,18 %	0,38 %	0,18 %	2,00	0,019
J47	Bronchiektasen	11	274	54	1.493	0,30 %	0,15 %	0,30 %	0,16 %	1,98	0,039
M35	Sonstige Krankheiten mit Systembeteiligung des Bindegewebes	137	3.851	688	19.320	3,71 %	1,86 %	4,17 %	2,09 %	1,98	< 0,001
D31	Gutartige Neubildung des Auges und der Augenanhangsgebilde	33	965	165	4.520	0,89 %	0,45 %	1,04 %	0,49 %	1,96	< 0,001
L25	Nicht näher bezeichnete Kontaktdermatitis	23	571	111	2.824	0,62 %	0,30 %	0,62 %	0,31 %	1,96	0,004
M40	Kyphose und Lordose	136	3.529	674	17.297	3,68 %	1,82 %	3,82 %	1,87 %	1,96	< 0,001
G90	Krankheiten des autonomen Nervensystems	17	432	83	2.101	0,46 %	0,22 %	0,47 %	0,23 %	1,95	0,013

Q43	Sonstige angeborene Fehlbildungen des Darmes	14	391	69	1.804	0,38 %	0,19 %	0,42 %	0,20 %	1,94	0,024
R23	Sonstige Hautveränderungen	56	1.479	279	7.238	1,51 %	0,75 %	1,60 %	0,78 %	1,93	< 0,001
R01	Herzgeräusche und andere Herz-Schallphänomene	19	541	96	2.505	0,51 %	0,26 %	0,59 %	0,27 %	1,92	0,010
K75	Sonstige entzündliche Leberkrankheiten	26	599	129	3.253	0,70 %	0,35 %	0,65 %	0,35 %	1,91	0,003
L23	Allergische Kontaktdermatitis	118	3.059	604	15.422	3,19 %	1,63 %	3,31 %	1,67 %	1,91	< 0,001
L30	Sonstige Dermatitis	505	12.887	2.727	71.653	13,66 %	7,38 %	13,95 %	7,76 %	1,90	< 0,001
I37	Pulmonalklappenkrankheiten	13	345	67	1.965	0,35 %	0,18 %	0,37 %	0,21 %	1,90	0,035
T15	Fremdkörper im äußeren Auge	17	394	85	2.063	0,46 %	0,23 %	0,43 %	0,22 %	1,89	0,017
R79	Sonstige abnorme Befunde der Blutchemie	21	535	107	2.871	0,57 %	0,29 %	0,58 %	0,31 %	1,89	0,008
L21	Seborrhoisches Ekzem	86	2.173	439	11.170	2,33 %	1,19 %	2,35 %	1,21 %	1,89	< 0,001
K14	Krankheiten der Zunge	26	649	131	3.348	0,70 %	0,35 %	0,70 %	0,36 %	1,88	0,003
I05	Rheumatische Mitralklappenkrankheiten	19	526	100	3.026	0,51 %	0,27 %	0,57 %	0,33 %	1,87	0,013
N23	Nicht näher bezeichnete Nierenkolik	15	359	75	1.834	0,41 %	0,20 %	0,39 %	0,20 %	1,87	0,027
Z88	Allergie gegenüber Arzneimitteln, Drogen oder biologisch aktiven	116	3.061	610	16.040	3,14 %	1,65 %	3,31 %	1,74 %	1,86	< 0,001

	Substanzen in der Eigenanamnese										
D89	Sonstige Störungen mit Beteiligung des Immunsystems, anderenorts nicht klassifiziert	13	346	68	1.790	0,35 %	0,18 %	0,37 %	0,19 %	1,84	0,045
S46	Verletzung von Muskeln und Sehnen in Höhe der Schulter und des Oberarmes	22	598	113	2.931	0,60 %	0,31 %	0,65 %	0,32 %	1,84	0,010
R59	Lymphknotenvergrö ßerung	44	1.141	230	5.642	1,19 %	0,62 %	1,24 %	0,61 %	1,83	< 0,001
H54	Blindheit und Sehbeeinträchtigung	71	1.958	379	10.708	1,92 %	1,03 %	2,12 %	1,16 %	1,83	< 0,001
J35	Chronische Krankheiten der Gaumenmandeln und der Rachenmandel	33	810	171	4.192	0,89 %	0,46 %	0,88 %	0,45 %	1,83	0,002
E73	Laktoseintoleranz	86	2.174	458	11.365	2,33 %	1,24 %	2,35 %	1,23 %	1,82	< 0,001
R16	Hepatomegalie und Splenomegalie, anderenorts nicht klassifiziert	52	1.253	274	6.914	1,41 %	0,74 %	1,36 %	0,75 %	1,82	< 0,001
C80	Bösartige Neubildung ohne Angabe der Lokalisation	41	1.160	223	6.365	1,11 %	0,60 %	1,26 %	0,69 %	1,81	< 0,001
R80	Isolierte Proteinurie	64	1.704	343	9.084	1,73 %	0,93 %	1,85 %	0,98 %	1,81	< 0,001
E29	Testikuläre Dysfunktion	23	539	120	2.850	0,62 %	0,32 %	0,58 %	0,31 %	1,80	0,010
D22	Melanozytennävus	693	17.108	4.008	99.348	18,74 %	10,84 %	18,52 %	10,76 %	1,80	< 0,001
D37	Neubildung unsicheren oder	37	967	201	5.514	1,00 %	0,54 %	1,05 %	0,60 %	1,80	0,001

	unbekannten Verhaltens der Mundhöhle und der Verdauungsorgane										
N40	Prostatahyperplasie	469	11.802	3.155	80.808	12,69 %	8,53 %	12,78 %	8,75 %	1,80	< 0,001
E65	Lokalisierte Adipositas	11	270	60	1.444	0,30 %	0,16 %	0,29 %	0,16 %	1,79	0,077
M86	Osteomyelitis	17	450	90	2.269	0,46 %	0,24 %	0,49 %	0,25 %	1,78	0,029
I85	Ösophagusvarizen	11	260	60	1.573	0,30 %	0,16 %	0,28 %	0,17 %	1,78	0,079
R33	Harnverhaltung	32	921	177	5.110	0,87 %	0,48 %	1,00 %	0,55 %	1,77	0,003
F91	Störungen des Sozialverhaltens	13	309	69	1.759	0,35 %	0,19 %	0,33 %	0,19 %	1,77	0,062
Q63	Sonstige angeborene Fehlbildungen der Niere	23	602	125	3.198	0,62 %	0,34 %	0,65 %	0,35 %	1,76	0,013
S76	Verletzung von Muskeln und Sehnen in Höhe der Hüfte und des Oberschenkels	17	400	90	2.185	0,46 %	0,24 %	0,43 %	0,24 %	1,76	0,032
M05	Seropositive chronische Polyarthritis	39	972	214	5.650	1,05 %	0,58 %	1,05 %	0,61 %	1,76	0,001
E55	Vitamin-D-Mangel	312	7.998	1.781	47.425	8,44 %	4,82 %	8,66 %	5,14 %	1,76	< 0,001
K30	Funktionelle Dyspepsie	34	847	185	4.835	0,92 %	0,50 %	0,92 %	0,52 %	1,74	0,003
S69	Sonstige und nicht näher bezeichnete Verletzungen des Handgelenkes und der Hand	10	241	54	1.337	0,27 %	0,15 %	0,26 %	0,14 %	1,74	0,108
M20	Erworbene Deformitäten der Finger und Zehen	249	6.773	1.433	37.972	6,74 %	3,88 %	7,33 %	4,11 %	1,74	< 0,001

M06	Sonstige chronische Polyarthritis	149	3.950	845	22.969	4,03 %	2,29 %	4,28 %	2,49 %	1,74	< 0,001
Q14	Angeborene Fehlbildung des hinteren Augenabschnittes	14	370	77	1.992	0,38 %	0,21 %	0,40 %	0,22 %	1,74	0,058
D40	Neubildung unsicheren oder unbekannten Verhaltens der männlichen Genitalorgane	19	456	104	2.607	0,51 %	0,28 %	0,49 %	0,28 %	1,74	0,028
K04	Krankheiten der Pulpa und des periapikalen Gewebes	11	287	61	1.518	0,30 %	0,16 %	0,31 %	0,16 %	1,73	0,094
R82	Sonstige abnorme Urinbefunde	33	883	184	4.896	0,89 %	0,50 %	0,96 %	0,53 %	1,73	0,004
M41	Skoliose	286	7.519	1.636	42.473	7,74 %	4,43 %	8,14 %	4,60 %	1,72	< 0,001
B00	Infektionen durch Herpesviren [Herpes simplex]	50	1.262	278	6.870	1,35 %	0,75 %	1,37 %	0,74 %	1,72	< 0,001
N50	Sonstige Krankheiten der männlichen Genitalorgane	33	784	180	4.286	0,89 %	0,49 %	0,85 %	0,46 %	1,72	0,004
F40	Phobische Störungen	90	2.225	504	12.218	2,43 %	1,36 %	2,41 %	1,32 %	1,72	< 0,001
J39	Sonstige Krankheiten der oberen Atemwege	24	600	133	3.384	0,65 %	0,36 %	0,65 %	0,37 %	1,71	0,016
D36	Gutartige Neubildung an sonstigen und nicht näher bezeichneten Lokalisationen	59	1.463	333	8.556	1,60 %	0,90 %	1,58 %	0,93 %	1,71	< 0,001

J84	Sonstige interstitielle Lungenkrankheiten	14	352	79	2.129	0,38 %	0,21 %	0,38 %	0,23 %	1,71	0,066
N42	Sonstige Krankheiten der Prostata	34	839	193	4.951	0,92 %	0,52 %	0,91 %	0,54 %	1,70	0,005
L71	Rosazea	167	4.411	964	24.964	4,52 %	2,61 %	4,78 %	2,70 %	1,70	< 0,001
J30	Vasomotorische und allergische Rhinopathie	585	14.271	3.494	84.973	15,82 %	9,45 %	15,45 %	9,20 %	1,69	< 0,001
K13	Sonstige Krankheiten der Lippe und der Mundschleimhaut	21	538	117	3.047	0,57 %	0,32 %	0,58 %	0,33 %	1,69	0,026
I08	Krankheiten mehrerer Herzklappen	20	573	116	3.486	0,54 %	0,31 %	0,62 %	0,38 %	1,69	0,031
B07	Viruswarzen	139	3.471	794	19.499	3,76 %	2,15 %	3,76 %	2,11 %	1,69	< 0,001
F54	Psychologische Faktoren oder Verhaltensfaktoren bei anderenorts klassifizierten Krankheiten	33	786	187	4.683	0,89 %	0,51 %	0,85 %	0,51 %	1,68	0,006
R35	Polyurie, Pollakisurie und Nykturie	60	1.575	347	9.076	1,62 %	0,94 %	1,71 %	0,98 %	1,68	< 0,001
T78	Unerwünschte Nebenwirkungen, anderenorts nicht klassifiziert	399	10.001	2.367	59.387	10,79 %	6,40 %	10,83 %	6,43 %	1,68	< 0,001
D21	Sonstige gutartige Neubildungen des Bindegewebes und anderer Weichteilgewebe	86	2.061	496	12.214	2,33 %	1,34 %	2,23 %	1,32 %	1,67	< 0,001

I83	Varizen der unteren Extremitäten	533	14.614	3.341	91.082	14,42 %	9,04 %	15,82 %	9,86 %	1,67	< 0,001
L64	Alopecia androgenetica	20	509	116	2.979	0,54 %	0,31 %	0,55 %	0,32 %	1,67	0,035
H36	Affektionen der Netzhaut bei anderenorts klassifizierten Krankheiten	80	2.094	472	13.242	2,16 %	1,28 %	2,27 %	1,43 %	1,66	< 0,001
M45	Spondylitis ankylosans	31	719	177	4.397	0,84 %	0,48 %	0,78 %	0,48 %	1,66	0,009
E61	Mangel an sonstigen Spurenelementen	71	1.800	415	10.722	1,92 %	1,12 %	1,95 %	1,16 %	1,66	< 0,001
Z84	Andere Krankheiten oder Zustände in der Familienanamnese	21	493	122	3.034	0,57 %	0,33 %	0,53 %	0,33 %	1,66	0,032
H18	Sonstige Affektionen der Hornhaut	67	1.865	393	10.976	1,81 %	1,06 %	2,02 %	1,19 %	1,66	< 0,001
E34	Sonstige endokrine Störungen	59	1.453	346	8.742	1,60 %	0,94 %	1,57 %	0,95 %	1,66	< 0,001
D39	Neubildung unsicheren oder unbekannten Verhaltens der weiblichen Genitalorgane	19	474	110	2.796	0,51 %	0,30 %	0,51 %	0,30 %	1,66	0,043
N87	Dysplasie der Cervix uteri	37	886	217	5.170	1,00 %	0,59 %	0,96 %	0,56 %	1,65	0,005
D24	Gutartige Neubildung der Brustdrüse [Mamma]	24	561	142	3.473	0,65 %	0,38 %	0,61 %	0,38 %	1,65	0,025
J32	Chronische Sinusitis	232	5.533	1.366	33.161	6,28 %	3,69 %	5,99 %	3,59 %	1,65	< 0,001
T88	Sonstige Komplikationen bei chirurgischen Eingriffen und	298	7.823	1.818	49.109	8,06 %	4,92 %	8,47 %	5,32 %	1,64	< 0,001

	medizinischer Behandlung, anderenorts nicht klassifiziert										
O99	Sonstige Krankheiten der Mutter, die anderenorts klassifizierbar sind, die jedoch Schwangerschaft, Geburt und Wochenbett komplizieren	23	565	139	3.452	0,62 %	0,38 %	0,61 %	0,37 %	1,64	0,035
L60	Krankheiten der Nägel	90	2.336	532	13.970	2,43 %	1,44 %	2,53 %	1,51 %	1,63	< 0,001
D52	Folsäure-Mangelanämie	16	445	96	2.793	0,43 %	0,26 %	0,48 %	0,30 %	1,63	0,072
L82	Seborrhoische Keratose	234	6.215	1.427	37.791	6,33 %	3,86 %	6,73 %	4,09 %	1,63	< 0,001
Q65	Angeborene Deformitäten der Hüfte	38	912	224	5.372	1,03 %	0,61 %	0,99 %	0,58 %	1,63	0,006
Z31	Fertilisationsfördernde Maßnahmen	51	1.226	301	7.204	1,38 %	0,81 %	1,33 %	0,78 %	1,63	0,002
K63	Sonstige Krankheiten des Darmes	182	4.783	1.108	28.757	4,92 %	3,00 %	5,18 %	3,11 %	1,63	< 0,001
N77	Vulvovaginale Ulzeration und Entzündung bei anderenorts klassifizierten Krankheiten	20	528	119	3.007	0,54 %	0,32 %	0,57 %	0,33 %	1,63	0,045
I78	Krankheiten der Kapillaren	32	779	189	4.800	0,87 %	0,51 %	0,84 %	0,52 %	1,62	0,012

N88	Sonstige nichtentzündliche Krankheiten der Cervix uteri	31	775	187	4.741	0,84 %	0,51 %	0,84 %	0,51 %	1,62	0,014
O36	Betreuung der Mutter wegen sonstiger festgestellter oder vermuteter Komplikationen beim Fetus	11	277	65	1.643	0,30 %	0,18 %	0,30 %	0,18 %	1,62	0,148
F61	Kombinierte und andere Persönlichkeitsstörungen	14	313	81	1.908	0,38 %	0,22 %	0,34 %	0,21 %	1,62	0,097
S63	Luxation, Verstauchung und Zerrung von Gelenken und Bändern in Höhe des Handgelenkes und der Hand	36	868	211	5.169	0,97 %	0,57 %	0,94 %	0,56 %	1,62	0,008
M15	Polyarthrose	212	6.082	1.325	38.001	5,73 %	3,58 %	6,59 %	4,11 %	1,62	< 0,001
K57	Divertikelkrankheit des Darmes	378	10.488	2.422	66.577	10,22 %	6,55 %	11,36 %	7,21 %	1,61	< 0,001
E04	Sonstige nichttoxische Struma	645	16.686	4.235	112.128	17,45 %	11,46 %	18,07 %	12,14 %	1,61	< 0,001
D50	Eisenmangelanämie	201	5.051	1.239	32.759	5,44 %	3,35 %	5,47 %	3,55 %	1,61	< 0,001
I38	Endokarditis, Herzklappe nicht näher bezeichnet	13	381	78	2.150	0,35 %	0,21 %	0,41 %	0,23 %	1,61	0,114
F52	Sexuelle Funktionsstörungen, nicht verursacht durch eine	162	3.843	995	24.131	4,38 %	2,69 %	4,16 %	2,61 %	1,61	< 0,001

	organische Störung oder Krankheit										
M85	Sonstige Veränderungen der Knochendichte und -struktur	54	1.489	327	8.964	1,46 %	0,88 %	1,61 %	0,97 %	1,61	0,001
S43	Luxation, Verstauchung und Zerrung von Gelenken und Bändern des Schultergürtels	21	508	124	3.032	0,57 %	0,34 %	0,55 %	0,33 %	1,60	0,048
K62	Sonstige Krankheiten des Anus und des Rektums	71	1.833	429	11.202	1,92 %	1,16 %	1,99 %	1,21 %	1,60	< 0,001
H93	Sonstige Krankheiten des Ohres, anderenorts nicht klassifiziert	280	7.088	1.734	44.507	7,57 %	4,69 %	7,68 %	4,82 %	1,60	< 0,001
L57	Hautveränderungen durch chronische Exposition gegenüber nichtionisierender Strahlung	174	4.912	1.108	31.703	4,71 %	3,00 %	5,32 %	3,43 %	1,59	< 0,001
J31	Chronische Rhinitis, Rhinopharyngitis und Pharyngitis	116	2.958	702	18.116	3,14 %	1,90 %	3,20 %	1,96 %	1,59	< 0,001
L03	Phlegmone	59	1.558	358	8.887	1,60 %	0,97 %	1,69 %	0,96 %	1,58	0,001
D23	Sonstige gutartige Neubildungen der Haut	127	3.172	776	19.327	3,44 %	2,10 %	3,43 %	2,09 %	1,58	< 0,001
F81	Umschriebene Entwicklungsstörung	12	279	70	1.631	0,32 %	0,19 %	0,30 %	0,18 %	1,57	0,157

	en schulischer Fertigkeiten										
N45	Orchitis und Epididymitis	14	312	80	1.880	0,38 %	0,22 %	0,34 %	0,20 %	1,57	0,124
D29	Gutartige Neubildung der männlichen Genitalorgane	48	1.253	299	7.693	1,30 %	0,81 %	1,36 %	0,83 %	1,57	0,005
M81	Osteoporose ohne pathologische Fraktur	244	7.428	1.623	50.750	6,60 %	4,39 %	8,04 %	5,50 %	1,57	< 0,001
Z83	Andere spezifische Krankheiten in der Familienanamnese	27	667	166	4.111	0,73 %	0,45 %	0,72 %	0,45 %	1,57	0,031
D75	Sonstige Krankheiten des Blutes und der blutbildenden Organe	30	712	183	4.664	0,81 %	0,49 %	0,77 %	0,50 %	1,56	0,024
K44	Hernia diaphragmatica	190	5.100	1.198	32.136	5,14 %	3,24 %	5,52 %	3,48 %	1,56	< 0,001
H27	Sonstige Affektionen der Linse	36	1.115	230	7.114	0,97 %	0,62 %	1,21 %	0,77 %	1,56	0,015
R63	Symptome, die die Nahrungs- und Flüssigkeitsaufnahm e betreffen	111	2.888	695	18.602	3,00 %	1,88 %	3,13 %	2,01 %	1,55	< 0,001
Z09	Nachuntersuchung nach Behandlung wegen anderer Krankheitszustände außer bösartigen Neubildungen	58	1.540	364	9.655	1,57 %	0,98 %	1,67 %	1,05 %	1,55	0,002
F98	Andere Verhaltens- und emotionale Störungen mit	19	459	115	2.752	0,51 %	0,31 %	0,50 %	0,30 %	1,55	0,082

	Beginn in der Kindheit und Jugend										
Z12	Spezielle Verfahren zur Untersuchung auf Neubildungen	1.562	38.554	11.868	293.032	42,25 %	32,10 %	41,75 %	31,73 %	1,55	< 0,001
N26	Schrumpfniere, nicht näher bezeichnet	19	568	120	3.434	0,51 %	0,32 %	0,61 %	0,37 %	1,54	0,081
K80	Cholelithiasis	231	6.043	1.485	40.456	6,25 %	4,02 %	6,54 %	4,38 %	1,54	< 0,001
K31	Sonstige Krankheiten des Magens und des Duodenums	70	1.888	439	11.451	1,89 %	1,19 %	2,04 %	1,24 %	1,54	< 0,001
E89	Endokrine und Stoffwechselstörungen nach medizinischen Maßnahmen, anderenorts nicht klassifiziert	117	3.092	750	20.678	3,16 %	2,03 %	3,35 %	2,24 %	1,54	< 0,001
C85	Sonstige und nicht näher bezeichnete Typen des Non-Hodgkin-Lymphoms	15	413	94	2.586	0,41 %	0,25 %	0,45 %	0,28 %	1,54	0,123
B18	Chronische Virushepatitis	16	396	100	2.416	0,43 %	0,27 %	0,43 %	0,26 %	1,53	0,113
D25	Leiomyom des Uterus	135	3.286	874	21.344	3,65 %	2,36 %	3,56 %	2,31 %	1,53	< 0,001
M21	Sonstige erworbene Deformitäten der Extremitäten	362	9.277	2.332	59.482	9,79 %	6,31 %	10,05 %	6,44 %	1,53	< 0,001
K64	Hämorrhoiden und Perianalvenenthrombose	322	8.138	2.074	52.685	8,71 %	5,61 %	8,81 %	5,70 %	1,53	< 0,001
D13	Gutartige Neubildung	19	512	120	3.046	0,51 %	0,32 %	0,55 %	0,33 %	1,53	0,085

	sonstiger und ungenau bezeichneter Teile des Verdauungssystems										
N80	Endometriose	38	892	243	5.714	1,03 %	0,66 %	0,97 %	0,62 %	1,53	0,016
D04	Carcinoma in situ der Haut	84	2.439	545	15.942	2,27 %	1,47 %	2,64 %	1,73 %	1,53	< 0,001
L85	Sonstige Epidermisverdickung	82	2.279	519	13.758	2,22 %	1,40 %	2,47 %	1,49 %	1,53	< 0,001
D69	Purpura und sonstige hämorrhagische Diathesen	60	1.560	379	10.216	1,62 %	1,03 %	1,69 %	1,11 %	1,53	0,003
L98	Sonstige Krankheiten der Haut und der Unterhaut, anderenorts nicht klassifiziert	54	1.483	342	9.311	1,46 %	0,93 %	1,61 %	1,01 %	1,53	0,004
D90	Immunkompromittierung nach Bestrahlung, Chemotherapie und sonstigen immunsuppressiven Maßnahmen	14	390	88	2.278	0,38 %	0,24 %	0,42 %	0,25 %	1,53	0,144
Q66	Angeborene Deformitäten der Füße	511	13.011	3.372	85.810	13,82 %	9,12 %	14,09 %	9,29 %	1,52	< 0,001
H04	Affektionen des Tränenapparates	263	7.111	1.729	47.542	7,11 %	4,68 %	7,70 %	5,15 %	1,52	< 0,001
M22	Krankheiten der Patella	111	2.705	696	16.681	3,00 %	1,88 %	2,93 %	1,81 %	1,52	< 0,001
N31	Neuromuskuläre Dysfunktion der Harnblase,	56	1.490	355	9.842	1,51 %	0,96 %	1,61 %	1,07 %	1,52	0,004

	anderenorts nicht klassifiziert										
R94	Abnorme Ergebnisse von Funktionsprüfungen	103	2.599	653	16.665	2,79 %	1,77 %	2,81 %	1,80 %	1,52	< 0,001
Q61	Zystische Nierenkrankheit	95	2.602	620	17.238	2,57 %	1,68 %	2,82 %	1,87 %	1,51	< 0,001
D80	Immundefekt mit vorherrschendem Antikörpermangel	16	412	101	2.419	0,43 %	0,27 %	0,45 %	0,26 %	1,51	0,126
M46	Sonstige entzündliche Spondylopathien	22	570	140	3.488	0,60 %	0,38 %	0,62 %	0,38 %	1,51	0,074
K29	Gastritis und Duodenitis	481	12.493	3.205	83.448	13,01 %	8,67 %	13,53 %	9,04 %	1,51	< 0,001
T82	Komplikationen durch Prothesen, Implantate oder Transplantate im Herzen und in den Gefäßen	16	435	103	2.921	0,43 %	0,28 %	0,47 %	0,32 %	1,51	0,129
U08	COVID-19 in der Eigenanamnese	229	5.463	1.479	36.551	6,19 %	4,00 %	5,92 %	3,96 %	1,50	< 0,001
R22	Lokalisierte Schwellung, Raumforderung und Knoten der Haut und der Unterhaut	23	568	146	3.727	0,62 %	0,39 %	0,62 %	0,40 %	1,50	0,071
Z25	Notwendigkeit der Impfung [Immunisierung] gegen andere einzelne Viruskrankheiten	1.023	27.580	7.721	212.551	27,67 %	20,88 %	29,86 %	23,02 %	1,50	< 0,001
H02	Sonstige Affektionen des Augenlides	89	2.507	582	16.425	2,41 %	1,57 %	2,71 %	1,78 %	1,50	< 0,001

H44	Affektionen des Augapfels	19	493	122	3.330	0,51 %	0,33 %	0,53 %	0,36 %	1,50	0,103
A46	Erysipel [Wundrose]	41	1.091	264	7.059	1,11 %	0,71 %	1,18 %	0,76 %	1,50	0,017
G56	Mononeuropathien der oberen Extremität	185	4.669	1.214	31.651	5,00 %	3,28 %	5,06 %	3,43 %	1,49	< 0,001
D84	Sonstige Immundefekte	10	240	64	1.637	0,27 %	0,17 %	0,26 %	0,18 %	1,49	0,242
T63	Toxische Wirkung durch Kontakt mit giftigen Tieren	40	987	255	6.418	1,08 %	0,69 %	1,07 %	0,69 %	1,49	0,019
L53	Sonstige erythematöse Krankheiten	13	342	83	2.127	0,35 %	0,22 %	0,37 %	0,23 %	1,49	0,182
R68	Sonstige Allgemeinsymptome	35	929	228	6.537	0,95 %	0,62 %	1,01 %	0,71 %	1,49	0,031
M18	Rhizarthrose [Arthrose des Daumensattelgelenkes]	119	3.295	788	21.385	3,22 %	2,13 %	3,57 %	2,32 %	1,48	< 0,001
J34	Sonstige Krankheiten der Nase und der Nasennebenhöhlen	192	4.678	1.252	30.881	5,19 %	3,39 %	5,07 %	3,34 %	1,48	< 0,001
Y57	Unerwünschte Nebenwirkungen bei therapeutischer Anwendung von Arzneimitteln und Drogen	26	691	169	4.860	0,70 %	0,46 %	0,75 %	0,53 %	1,48	0,066
K82	Sonstige Krankheiten der Gallenblase	32	786	209	5.467	0,87 %	0,57 %	0,85 %	0,59 %	1,48	0,042
I82	Sonstige venöse Embolie und Thrombose	36	959	236	6.465	0,97 %	0,64 %	1,04 %	0,70 %	1,48	0,031

N08	Glomeruläre Krankheiten bei anderenorts klassifizierten Krankheiten	100	2.661	669	19.180	2,70 %	1,81 %	2,88 %	2,08 %	1,47	< 0,001
R19	Sonstige Symptome, die das Verdauungssystem und das Abdomen betreffen	40	984	261	6.645	1,08 %	0,71 %	1,07 %	0,72 %	1,47	0,024
M47	Spondylose	688	18.583	4.865	129.475	18,61 %	13,16 %	20,12 %	14,02 %	1,47	< 0,001
N41	Entzündliche Krankheiten der Prostata	26	666	172	4.285	0,70 %	0,47 %	0,72 %	0,46 %	1,47	0,071
D64	Sonstige Anämien	128	3.640	858	23.858	3,46 %	2,32 %	3,94 %	2,58 %	1,47	< 0,001
E21	Hyperparathyreoidismus und sonstige Krankheiten der Nebenschilddrüse	28	738	185	4.993	0,76 %	0,50 %	0,80 %	0,54 %	1,46	0,061
F42	Zwangsstörung	29	681	184	4.331	0,78 %	0,50 %	0,74 %	0,47 %	1,46	0,059
H73	Sonstige Krankheiten des Trommelfells	37	914	242	6.423	1,00 %	0,65 %	0,99 %	0,70 %	1,46	0,032
Z04	Untersuchung und Beobachtung aus sonstigen Gründen	31	822	202	5.263	0,84 %	0,55 %	0,89 %	0,57 %	1,46	0,051
F45	Somatoforme Störungen	666	16.807	4.652	119.232	18,01 %	12,58 %	18,20 %	12,91 %	1,46	< 0,001
I73	Sonstige periphere Gefäßkrankheiten	93	2.550	624	16.907	2,52 %	1,69 %	2,76 %	1,83 %	1,46	< 0,001
N25	Krankheiten infolge Schädigung der tubulären Nierenfunktion	14	410	93	2.633	0,38 %	0,25 %	0,44 %	0,29 %	1,46	0,192
N60	Gutartige Mammadysplasie	75	1.824	503	12.269	2,03 %	1,36 %	1,98 %	1,33 %	1,46	0,003

	[Brustdrüsendysplasie]										
Z13	Spezielle Verfahren zur Untersuchung auf sonstige Krankheiten oder Störungen	64	1.614	421	10.773	1,73 %	1,14 %	1,75 %	1,17 %	1,45	0,006
R41	Sonstige Symptome, die das Erkennungsvermögen und das Bewusstsein betreffen	61	1.855	414	12.640	1,65 %	1,12 %	2,01 %	1,37 %	1,45	0,008
D12	Gutartige Neubildung des Kolons, des Rektums, des Analkanals und des Anus	103	2.695	690	17.941	2,79 %	1,87 %	2,92 %	1,94 %	1,45	< 0,001
R20	Sensibilitätsstörungen der Haut	116	2.926	772	19.579	3,14 %	2,09 %	3,17 %	2,12 %	1,45	< 0,001
S80	Oberflächliche Verletzung des Unterschenkels	53	1.380	351	9.446	1,43 %	0,95 %	1,49 %	1,02 %	1,45	0,012
H90	Hörverlust durch Schallleitungs- oder Schallempfindungsstörung	288	8.123	2.006	56.485	7,79 %	5,43 %	8,80 %	6,12 %	1,44	< 0,001
M19	Sonstige Arthrose	436	11.884	3.054	81.716	11,79 %	8,26 %	12,87 %	8,85 %	1,44	< 0,001
R77	Sonstige Veränderungen der Plasmaproteine	33	830	222	5.713	0,89 %	0,60 %	0,90 %	0,62 %	1,44	0,054
H91	Sonstiger Hörverlust	247	7.008	1.717	48.983	6,68 %	4,64 %	7,59 %	5,30 %	1,44	< 0,001
S92	Fraktur des Fußes [ausgenommen	32	785	212	5.261	0,87 %	0,57 %	0,85 %	0,57 %	1,44	0,055

	oberes Sprunggelenk]										
I67	Sonstige zerebrovaskuläre Krankheiten	160	4.747	1.110	32.268	4,33 %	3,00 %	5,14 %	3,49 %	1,44	< 0,001
Z82	Bestimmte Behinderungen oder chronische Krankheiten in der Familienanamnese, die zu Schädigung oder Behinderung führen	44	1.081	293	7.204	1,19 %	0,79 %	1,17 %	0,78 %	1,44	0,025
E14	Nicht näher bezeichneter Diabetes mellitus	301	7.947	2.110	58.058	8,14 %	5,71 %	8,61 %	6,29 %	1,43	< 0,001
E01	Jodmangelbedingte Schilddrüsenkrankheiten und verwandte Zustände	85	2.247	575	15.159	2,30 %	1,56 %	2,43 %	1,64 %	1,43	0,002
T08	Fraktur der Wirbelsäule, Höhe nicht näher bezeichnet	24	825	163	5.052	0,65 %	0,44 %	0,89 %	0,55 %	1,43	0,104
I24	Sonstige akute ischämische Herzkrankheit	10	277	67	1.774	0,27 %	0,18 %	0,30 %	0,19 %	1,43	0,292
N95	Klimakterische Störungen	585	15.283	4.494	117.106	15,82 %	12,16 %	16,55 %	12,68 %	1,43	< 0,001
Z97	Vorhandensein anderer medizinischer Geräte oder Hilfsmittel	88	2.364	602	17.319	2,38 %	1,63 %	2,56 %	1,88 %	1,43	0,002
M79	Sonstige Krankheiten des Weichteilgewebes,	517	13.071	3.615	92.090	13,98 %	9,78 %	14,15 %	9,97 %	1,43	< 0,001

	anderenorts nicht klassifiziert									
E56	Sonstige Vitaminmangelzustände	21	585	142	3.725	0,57 %	0,38 %	0,63 %	0,40 %	1,43	0,130
O80	Spontangeburt eines Einlings	11	265	75	1.876	0,30 %	0,20 %	0,29 %	0,20 %	1,42	0,287
Q21	Angeborene Fehlbildungen der Herzsepten	22	676	149	3.816	0,60 %	0,40 %	0,73 %	0,41 %	1,42	0,127
M75	Schulterläsionen	402	10.172	2.813	71.017	10,87 %	7,61 %	11,01 %	7,69 %	1,42	< 0,001
Z72	Probleme mit Bezug auf die Lebensführung	26	594	175	4.300	0,70 %	0,47 %	0,64 %	0,47 %	1,42	0,098
Z24	Notwendigkeit der Impfung [Immunisierung] gegen bestimmte einzelne Viruskrankheiten	240	5.949	1.651	40.796	6,49 %	4,47 %	6,44 %	4,42 %	1,42	< 0,001
N84	Polyp des weiblichen Genitaltraktes	25	613	171	4.247	0,68 %	0,46 %	0,66 %	0,46 %	1,41	0,109
R45	Symptome, die die Stimmung betreffen	100	2.631	683	18.431	2,70 %	1,85 %	2,85 %	2,00 %	1,41	0,001
B97	Viren als Ursache von Krankheiten, die in anderen Kapiteln klassifiziert sind	20	534	137	3.483	0,54 %	0,37 %	0,58 %	0,38 %	1,41	0,151
G43	Migräne	316	7.775	2.221	53.854	8,55 %	6,01 %	8,42 %	5,83 %	1,41	< 0,001
K10	Sonstige Krankheiten der Kiefer	10	261	68	1.717	0,27 %	0,18 %	0,28 %	0,19 %	1,41	0,312
H16	Keratitis	35	910	237	6.209	0,95 %	0,64 %	0,99 %	0,67 %	1,41	0,060
Z91	Risikofaktoren in der Eigenanamnese,	53	1.375	362	9.806	1,43 %	0,98 %	1,49 %	1,06 %	1,41	0,022

	anderenorts nicht klassifiziert										
Z96	Vorhandensein von anderen funktionellen Implantaten	497	14.660	3.779	113.869	13,44 %	10,22 %	15,87 %	12,33 %	1,41	< 0,001
K76	Sonstige Krankheiten der Leber	448	11.516	3.190	82.570	12,12 %	8,63 %	12,47 %	8,94 %	1,40	< 0,001
L70	Akne	98	2.297	667	15.876	2,65 %	1,80 %	2,49 %	1,72 %	1,40	0,002
I13	Hypertensive Herz- und Nierenkrankheit	19	501	132	3.857	0,51 %	0,36 %	0,54 %	0,42 %	1,40	0,175
Z00	Allgemeinuntersuchung und Abklärung bei Personen ohne Beschwerden oder angegebene Diagnose	822	20.417	5.975	148.707	22,23 %	16,16 %	22,11 %	16,10 %	1,40	< 0,001
I79	Krankheiten der Arterien, Arteriolen und Kapillaren bei anderenorts klassifizierten Krankheiten	37	1.001	258	7.271	1,00 %	0,70 %	1,08 %	0,79 %	1,40	0,058
K85	Akute Pankreatitis	16	421	108	2.876	0,43 %	0,29 %	0,46 %	0,31 %	1,40	0,212
G24	Dystonie	13	370	88	2.401	0,35 %	0,24 %	0,40 %	0,26 %	1,40	0,260
S13	Luxation, Verstauchung und Zerrung von Gelenken und Bändern in Halshöhe	23	556	156	3.913	0,62 %	0,42 %	0,60 %	0,42 %	1,39	0,140
H52	Akkommodationsstörungen und Refraktionsfehler	1.060	27.572	8.094	213.961	28,67 %	21,89 %	29,85 %	23,17 %	1,39	< 0,001
H20	Iridozyklitis	13	299	90	2.303	0,35 %	0,24 %	0,32 %	0,25 %	1,39	0,269

R27	Sonstige Koordinationsstörungen	18	533	125	3.429	0,49 %	0,34 %	0,58 %	0,37 %	1,39	0,193
Z80	Bösartige Neubildung in der Familienanamnese	173	4.395	1.222	29.912	4,68 %	3,31 %	4,76 %	3,24 %	1,39	< 0,001
M96	Krankheiten des Muskel-Skelett-Systems nach medizinischen Maßnahmen, anderenorts nicht klassifiziert	22	569	154	3.977	0,60 %	0,42 %	0,62 %	0,43 %	1,39	0,151
H10	Konjunktivitis	235	5.768	1.639	41.229	6,36 %	4,43 %	6,25 %	4,46 %	1,39	< 0,001
G63	Polyneuropathie bei anderenorts klassifizierten Krankheiten	168	4.650	1.207	34.408	4,54 %	3,26 %	5,04 %	3,73 %	1,39	< 0,001
M87	Knochennekrose	11	273	75	1.974	0,30 %	0,20 %	0,30 %	0,21 %	1,39	0,314
G93	Sonstige Krankheiten des Gehirns	64	1.680	444	11.757	1,73 %	1,20 %	1,82 %	1,27 %	1,39	0,016
C73	Bösartige Neubildung der Schilddrüse	10	246	69	1.794	0,27 %	0,19 %	0,27 %	0,19 %	1,38	0,338
J11	Grippe, Viren nicht nachgewiesen	34	817	231	5.555	0,92 %	0,62 %	0,88 %	0,60 %	1,38	0,080
L73	Sonstige Krankheiten der Haarfollikel	47	1.153	320	7.753	1,27 %	0,87 %	1,25 %	0,84 %	1,38	0,040
J45	Asthma bronchiale	441	10.618	3.137	78.569	11,93 %	8,49 %	11,50 %	8,51 %	1,38	< 0,001
H92	Otalgie und Ohrenfluss	71	1.740	489	12.171	1,92 %	1,32 %	1,88 %	1,32 %	1,38	0,012
R00	Störungen des Herzschlages	182	4.771	1.278	33.396	4,92 %	3,46 %	5,17 %	3,62 %	1,38	< 0,001

K60	Fissur und Fistel in der Anal- und Rektalregion	30	757	207	5.023	0,81 %	0,56 %	0,82 %	0,54 %	1,38	0,102
F34	Anhaltende affektive Störungen	86	2.109	599	14.924	2,33 %	1,62 %	2,28 %	1,62 %	1,38	0,006
M99	Biomechanische Funktionsstörungen, anderenorts nicht klassifiziert	534	13.257	3.854	95.714	14,44 %	10,42 %	14,35 %	10,36 %	1,38	< 0,001
M77	Sonstige Enthesopathien	409	9.966	2.930	72.224	11,06 %	7,93 %	10,79 %	7,82 %	1,38	< 0,001
G57	Mononeuropathien der unteren Extremität	54	1.425	377	9.739	1,46 %	1,02 %	1,54 %	1,05 %	1,37	0,031
G25	Sonstige extrapyramidale Krankheiten und Bewegungsstörunge n	111	3.108	790	22.159	3,00 %	2,14 %	3,37 %	2,40 %	1,37	0,002
M71	Sonstige Bursopathien	62	1.602	434	11.152	1,68 %	1,17 %	1,73 %	1,21 %	1,37	0,021
I95	Hypotonie	124	3.337	877	23.396	3,35 %	2,37 %	3,61 %	2,53 %	1,37	0,001
E78	Störungen des Lipoproteinstoffwec hsels und sonstige Lipidämien	1.319	34.806	10.639	283.100	35,68 %	28,78 %	37,69 %	30,65 %	1,37	< 0,001
K83	Sonstige Krankheiten der Gallenwege	12	296	84	2.413	0,32 %	0,23 %	0,32 %	0,26 %	1,37	0,309
Z53	Personen, die Einrichtungen des Gesundheitswesens wegen spezifischer Maßnahmen aufgesucht haben, die aber nicht	10	285	70	1.854	0,27 %	0,19 %	0,31 %	0,20 %	1,37	0,354

	durchgeführt wurden										
N30	Zystitis	143	3.846	1.019	27.294	3,87 %	2,76 %	4,16 %	2,96 %	1,37	< 0,001
N81	Genitalprolaps bei der Frau	125	3.513	906	25.091	3,38 %	2,45 %	3,80 %	2,72 %	1,37	0,002
Z08	Nachuntersuchung nach Behandlung wegen bösartiger Neubildung	91	2.541	651	17.918	2,46 %	1,76 %	2,75 %	1,94 %	1,37	0,007
J00	Akute Rhinopharyngitis [Erkältungsschnupfe n]	176	4.199	1.233	28.965	4,76 %	3,34 %	4,55 %	3,14 %	1,36	< 0,001
K59	Sonstige funktionelle Darmstörungen	158	4.301	1.137	32.599	4,27 %	3,08 %	4,66 %	3,53 %	1,36	< 0,001
R73	Erhöhter Blutglukosewert	103	2.622	730	18.933	2,79 %	1,97 %	2,84 %	2,05 %	1,36	0,004
H01	Sonstige Entzündung des Augenlides	67	1.837	477	12.636	1,81 %	1,29 %	1,99 %	1,37 %	1,36	0,020
K40	Hernia inguinalis	77	1.954	545	13.912	2,08 %	1,47 %	2,12 %	1,51 %	1,36	0,014
M24	Sonstige näher bezeichnete Gelenkschädigungen	63	1.551	440	10.861	1,70 %	1,19 %	1,68 %	1,18 %	1,36	0,025
M10	Gicht	159	4.195	1.152	30.159	4,30 %	3,12 %	4,54 %	3,27 %	1,36	< 0,001
Z35	Überwachung einer Risikoschwangersch aft	22	530	160	3.914	0,60 %	0,43 %	0,57 %	0,42 %	1,35	0,205
M17	Gonarthrose [Arthrose des Kniegelenkes]	557	15.147	4.227	116.715	15,07 %	11,43 %	16,40 %	12,64 %	1,35	< 0,001
F48	Andere neurotische Störungen	209	5.044	1.491	35.860	5,65 %	4,03 %	5,46 %	3,88 %	1,35	< 0,001

D34	Gutartige Neubildung der Schilddrüse	18	506	129	3.532	0,49 %	0,35 %	0,55 %	0,38 %	1,35	0,233
Z26	Notwendigkeit der Impfung [Immunisierung] gegen andere einzelne Infektionskrankheiten	250	6.473	1.805	46.836	6,76 %	4,88 %	7,01 %	5,07 %	1,35	< 0,001
K22	Sonstige Krankheiten des Ösophagus	58	1.530	414	10.885	1,57 %	1,12 %	1,66 %	1,18 %	1,35	0,034
Q82	Sonstige angeborene Fehlbildungen der Haut	26	656	184	4.694	0,70 %	0,50 %	0,71 %	0,51 %	1,35	0,158
O26	Betreuung der Mutter bei sonstigen Zuständen, die vorwiegend mit der Schwangerschaft verbunden sind	20	493	146	3.635	0,54 %	0,39 %	0,53 %	0,39 %	1,35	0,231
R69	Unbekannte und nicht näher bezeichnete Krankheitsursachen	20	525	143	3.669	0,54 %	0,39 %	0,57 %	0,40 %	1,34	0,217
M65	Synovitis und Tenosynovitis	146	3.602	1.044	26.133	3,95 %	2,82 %	3,90 %	2,83 %	1,34	0,001
S86	Verletzung von Muskeln und Sehnen in Höhe des Unterschenkels	14	326	97	2.319	0,38 %	0,26 %	0,35 %	0,25 %	1,34	0,303
M95	Sonstige erworbene Deformitäten des Muskel-Skelett-	38	927	270	6.800	1,03 %	0,73 %	1,00 %	0,74 %	1,34	0,091

	Systems und des Bindegewebes										
I87	Sonstige Venenkrankheiten	241	6.779	1.778	48.808	6,52 %	4,81 %	7,34 %	5,28 %	1,34	< 0,001
H26	Sonstige Kataraktformen	337	9.889	2.577	75.309	9,12 %	6,97 %	10,71 %	8,15 %	1,34	< 0,001
Z49	Dialysebehandlung	11	338	80	2.177	0,30 %	0,22 %	0,37 %	0,24 %	1,34	0,365
M72	Fibromatosen	114	2.857	823	20.564	3,08 %	2,23 %	3,09 %	2,23 %	1,34	0,004
R12	Sodbrennen	57	1.436	408	10.366	1,54 %	1,10 %	1,56 %	1,12 %	1,33	0,043
G58	Sonstige Mononeuropathien	46	1.110	328	8.137	1,24 %	0,89 %	1,20 %	0,88 %	1,33	0,071
K42	Hernia umbilicalis	43	1.030	309	7.612	1,16 %	0,84 %	1,11 %	0,82 %	1,33	0,082
N18	Chronische Nierenkrankheit	293	8.434	2.258	68.379	7,93 %	6,11 %	9,13 %	7,40 %	1,33	< 0,001
J04	Akute Laryngitis und Tracheitis	72	1.791	516	12.681	1,95 %	1,40 %	1,94 %	1,37 %	1,33	0,025
Z32	Untersuchung und Test zur Feststellung einer Schwangerschaft	17	410	124	3.047	0,46 %	0,34 %	0,44 %	0,33 %	1,33	0,282
M42	Osteochondrose der Wirbelsäule	345	8.965	2.567	67.635	9,33 %	6,94 %	9,71 %	7,32 %	1,33	< 0,001
G62	Sonstige Polyneuropathien	183	5.115	1.363	38.824	4,95 %	3,69 %	5,54 %	4,20 %	1,33	< 0,001
C43	Bösartiges Melanom der Haut	34	911	248	6.545	0,92 %	0,67 %	0,99 %	0,71 %	1,33	0,126
H43	Affektionen des Glaskörpers	157	4.444	1.165	32.385	4,25 %	3,15 %	4,81 %	3,51 %	1,32	0,001
I49	Sonstige kardiale Arrhythmien	258	7.058	1.937	53.950	6,98 %	5,24 %	7,64 %	5,84 %	1,32	< 0,001
K86	Sonstige Krankheiten des Pankreas	53	1.408	385	10.496	1,43 %	1,04 %	1,52 %	1,14 %	1,32	0,060
Z71	Personen, die das Gesundheitswesen zum Zwecke anderer	323	8.015	2.381	59.871	8,74 %	6,44 %	8,68 %	6,48 %	1,32	< 0,001

	Beratung oder ärztlicher Konsultation in Anspruch nehmen, anderenorts nicht klassifiziert										
R07	Hals- und Brustschmerzen	214	5.254	1.560	38.819	5,79 %	4,22 %	5,69 %	4,20 %	1,32	< 0,001
J20	Akute Bronchitis	183	4.554	1.328	33.016	4,95 %	3,59 %	4,93 %	3,58 %	1,32	< 0,001
I45	Sonstige kardiale Erregungsleitungsst örungen	69	1.883	509	13.786	1,87 %	1,38 %	2,04 %	1,49 %	1,32	0,036
C18	Bösartige Neubildung des Kolons	32	866	238	6.840	0,87 %	0,64 %	0,94 %	0,74 %	1,32	0,149
N86	Erosion und Ektropium der Cervix uteri	70	1.690	519	12.370	1,89 %	1,40 %	1,83 %	1,34 %	1,31	0,037
H57	Sonstige Affektionen des Auges und der Augenanhangsgebild e	47	1.213	343	9.186	1,27 %	0,93 %	1,31 %	0,99 %	1,31	0,081
S05	Verletzung des Auges und der Orbita	13	329	95	2.407	0,35 %	0,26 %	0,36 %	0,26 %	1,31	0,361
M50	Zervikale Bandscheibenschäd en	156	3.791	1.151	28.621	4,22 %	3,11 %	4,11 %	3,10 %	1,31	0,002
Z63	Andere Kontaktanlässe mit Bezug auf den engeren Familienkreis	29	725	210	5.185	0,78 %	0,57 %	0,78 %	0,56 %	1,31	0,177
C20	Bösartige Neubildung des Rektums	12	328	89	2.433	0,32 %	0,24 %	0,35 %	0,26 %	1,31	0,382

B37	Kandidose	81	2.108	597	15.487	2,19 %	1,61 %	2,28 %	1,68 %	1,31	0,026
K51	Colitis ulcerosa	34	844	249	6.219	0,92 %	0,67 %	0,91 %	0,67 %	1,31	0,144
K91	Krankheiten des Verdauungssystems nach medizinischen Maßnahmen, anderenorts nicht klassifiziert	21	536	155	4.244	0,57 %	0,42 %	0,58 %	0,46 %	1,31	0,252
J02	Akute Pharyngitis	135	3.244	979	23.224	3,65 %	2,65 %	3,51 %	2,51 %	1,31	0,005
M23	Binnenschädigung des Kniegelenkes [internal derangement]	250	6.143	1.839	45.122	6,76 %	4,97 %	6,65 %	4,89 %	1,31	< 0,001
N76	Sonstige entzündliche Krankheit der Vagina und Vulva	157	3.890	1.181	29.572	4,25 %	3,19 %	4,21 %	3,20 %	1,31	0,003
N28	Sonstige Krankheiten der Niere und des Ureters, anderenorts nicht klassifiziert	187	5.083	1.412	38.522	5,06 %	3,82 %	5,50 %	4,17 %	1,30	0,001
L72	Follikuläre Zysten der Haut und der Unterhaut	71	1.752	519	13.009	1,92 %	1,40 %	1,90 %	1,41 %	1,30	0,038
Z30	Kontrazeptive Maßnahmen	570	13.247	4.865	113.315	15,42 %	13,16 %	14,34 %	12,27 %	1,30	< 0,001
R10	Bauch- und Beckenschmerzen	538	13.354	4.092	101.838	14,55 %	11,07 %	14,46 %	11,03 %	1,30	< 0,001
M51	Sonstige Bandscheibenschäd en	561	14.243	4.279	107.912	15,17 %	11,57 %	15,42 %	11,68 %	1,30	< 0,001
G55	Kompression von Nervenwurzeln und Nervenplexus bei	165	4.204	1.224	30.993	4,46 %	3,31 %	4,55 %	3,36 %	1,30	0,002

	anderenorts klassifizierten Krankheiten										
E28	Ovarielle Dysfunktion	51	1.259	384	9.659	1,38 %	1,04 %	1,36 %	1,05 %	1,30	0,084
D72	Sonstige Krankheiten der Leukozyten	27	728	199	5.126	0,73 %	0,54 %	0,79 %	0,56 %	1,30	0,207
M25	Sonstige Gelenkkrankheiten, anderenorts nicht klassifiziert	536	13.454	4.076	102.680	14,50 %	11,03 %	14,57 %	11,12 %	1,30	< 0,001
N92	Zu starke, zu häufige oder unregelmäßige Menstruation	167	3.830	1.291	29.810	4,52 %	3,49 %	4,15 %	3,23 %	1,30	0,004
B95	Streptokokken und Staphylokokken als Ursache von Krankheiten, die in anderen Kapiteln klassifiziert sind	50	1.369	373	10.463	1,35 %	1,01 %	1,48 %	1,13 %	1,30	0,089
Z73	Probleme mit Bezug auf Schwierigkeiten bei der Lebensbewältigung	90	2.097	664	16.004	2,43 %	1,80 %	2,27 %	1,73 %	1,29	0,024
H81	Störungen der Vestibularfunktion	107	2.854	803	21.866	2,89 %	2,17 %	3,09 %	2,37 %	1,29	0,014
A69	Sonstige Spirochäteninfektion en	33	847	244	6.243	0,89 %	0,66 %	0,92 %	0,68 %	1,29	0,171
S40	Oberflächliche Verletzung der Schulter und des Oberarmes	26	662	192	5.172	0,70 %	0,52 %	0,72 %	0,56 %	1,29	0,229
M62	Sonstige Muskelkrankheiten	316	7.974	2.386	60.730	8,55 %	6,45 %	8,63 %	6,58 %	1,29	< 0,001

M48	Sonstige Spondylopathien	233	6.500	1.784	49.934	6,30 %	4,83 %	7,04 %	5,41 %	1,29	< 0,001
H61	Sonstige Krankheiten des äußeren Ohres	444	11.910	3.421	93.059	12,01 %	9,25 %	12,90 %	10,08 %	1,29	< 0,001
M54	Rückenschmerzen	1.459	37.018	11.898	301.291	39,46 %	32,18 %	40,08 %	32,62 %	1,29	< 0,001
E79	Störungen des Purin- und Pyrimidinstoffwechsels	319	8.364	2.476	66.903	8,63 %	6,70 %	9,06 %	7,24 %	1,29	< 0,001
I77	Sonstige Krankheiten der Arterien und Arteriolen	20	543	150	4.129	0,54 %	0,41 %	0,59 %	0,45 %	1,28	0,294
F41	Andere Angststörungen	367	9.199	2.798	70.542	9,93 %	7,57 %	9,96 %	7,64 %	1,28	< 0,001
F32	Depressive Episode	697	17.662	5.428	138.778	18,85 %	14,68 %	19,12 %	15,03 %	1,28	< 0,001
G50	Krankheiten des N. trigeminus [V. Hirnnerv]	21	549	158	4.323	0,57 %	0,43 %	0,59 %	0,47 %	1,28	0,284
F60	Spezifische Persönlichkeitsstörungen	56	1.374	414	10.148	1,51 %	1,12 %	1,49 %	1,10 %	1,28	0,084
I34	Nichtrheumatische Mitralklappenkrankheiten	201	5.695	1.560	44.974	5,44 %	4,22 %	6,17 %	4,87 %	1,28	0,002
Z47	Andere orthopädische Nachbehandlung	18	444	134	3.420	0,49 %	0,36 %	0,48 %	0,37 %	1,28	0,325
H00	Hordeolum und Chalazion	56	1.439	417	10.400	1,51 %	1,13 %	1,56 %	1,13 %	1,28	0,085
K81	Cholezystitis	13	367	98	2.612	0,35 %	0,27 %	0,40 %	0,28 %	1,28	0,405
Z23	Notwendigkeit der Impfung [Immunisierung] gegen einzelne	122	3.225	929	24.944	3,30 %	2,51 %	3,49 %	2,70 %	1,28	0,013

	bakterielle Krankheiten										
R53	Unwohlsein und Ermüdung	269	6.679	2.038	51.677	7,28 %	5,51 %	7,23 %	5,60 %	1,28	< 0,001
R30	Schmerzen beim Wasserlassen	52	1.298	392	9.920	1,41 %	1,06 %	1,41 %	1,07 %	1,27	0,103
N89	Sonstige nichtentzündliche Krankheiten der Vagina	624	15.384	5.115	124.661	16,88 %	13,84 %	16,66 %	13,50 %	1,27	< 0,001
I51	Komplikationen einer Herzkrankheit und ungenau beschriebene Herzkrankheit	110	3.004	846	23.757	2,98 %	2,29 %	3,25 %	2,57 %	1,27	0,022
Z27	Notwendigkeit der Impfung [Immunisierung] gegen Kombinationen von Infektionskrankheite n	265	6.546	2.008	49.671	7,17 %	5,43 %	7,09 %	5,38 %	1,27	< 0,001
M89	Sonstige Knochenkrankheiten	27	690	204	5.150	0,73 %	0,55 %	0,75 %	0,56 %	1,27	0,251
U07	Krankheiten mit unklarer Ätiologie, belegte und nicht belegte Schlüsselnummern U07.-	411	9.965	3.149	75.851	11,12 %	8,52 %	10,79 %	8,21 %	1,27	< 0,001
R04	Blutung aus den Atemwegen	43	1.170	324	8.593	1,16 %	0,88 %	1,27 %	0,93 %	1,27	0,149
Z03	Ärztliche Beobachtung und Beurteilung von Verdachtsfällen,	85	2.177	646	17.222	2,30 %	1,75 %	2,36 %	1,86 %	1,26	0,045

	Verdacht ausgeschlossen										
H59	Affektionen des Auges und der Augenanhangsgebilde nach medizinischen Maßnahmen, anderenorts nicht klassifiziert	25	691	193	5.585	0,68 %	0,52 %	0,75 %	0,60 %	1,26	0,273
O09	Schwangerschaftsdauer	41	1.011	320	7.959	1,11 %	0,87 %	1,09 %	0,86 %	1,26	0,193
Z51	Sonstige medizinische Behandlung	174	4.458	1.334	35.803	4,71 %	3,61 %	4,83 %	3,88 %	1,26	0,005
M43	Sonstige Deformitäten der Wirbelsäule und des Rückens	138	3.682	1.059	28.300	3,73 %	2,86 %	3,99 %	3,06 %	1,26	0,012
M13	Sonstige Arthritis	80	2.030	606	15.543	2,16 %	1,64 %	2,20 %	1,68 %	1,26	0,053
I15	Sekundäre Hypertonie	24	617	184	5.118	0,65 %	0,50 %	0,67 %	0,55 %	1,26	0,286
R31	Nicht näher bezeichnete Hämaturie	118	3.068	908	23.899	3,19 %	2,46 %	3,32 %	2,59 %	1,26	0,020
G45	Zerebrale transitorische Ischämie und verwandte Syndrome	41	1.210	317	9.171	1,11 %	0,86 %	1,31 %	0,99 %	1,26	0,168
K56	Paralytischer Ileus und intestinale Obstruktion ohne Hernie	29	815	222	6.191	0,78 %	0,60 %	0,88 %	0,67 %	1,26	0,243
D48	Neubildung unsicheren oder	95	2.510	725	18.678	2,57 %	1,96 %	2,72 %	2,02 %	1,26	0,037

	unbekannten Verhaltens an sonstigen und nicht näher bezeichneten Lokalisationen										
U11	Notwendigkeit der Impfung gegen COVID-19	389	10.758	3.126	87.680	10,52 %	8,46 %	11,65 %	9,49 %	1,26	< 0,001
K66	Sonstige Krankheiten des Peritoneums	30	784	231	6.110	0,81 %	0,62 %	0,85 %	0,66 %	1,26	0,240
M94	Sonstige Knorpelkrankheiten	67	1.591	503	12.132	1,81 %	1,36 %	1,72 %	1,31 %	1,26	0,082
R74	Abnorme Serumenzymwerte	60	1.574	453	11.251	1,62 %	1,23 %	1,70 %	1,22 %	1,26	0,099
M16	Koxarthrose [Arthrose des Hüftgelenkes]	298	8.349	2.355	66.919	8,06 %	6,37 %	9,04 %	7,25 %	1,26	< 0,001
G99	Sonstige Krankheiten des Nervensystems bei anderenorts klassifizierten Krankheiten	13	358	99	2.587	0,35 %	0,27 %	0,39 %	0,28 %	1,26	0,442
N93	Sonstige abnorme Uterus- oder Vaginalblutung	35	806	269	6.375	0,95 %	0,73 %	0,87 %	0,69 %	1,26	0,212
N32	Sonstige Krankheiten der Harnblase	53	1.392	410	11.280	1,43 %	1,11 %	1,51 %	1,22 %	1,26	0,123
H53	Sehstörungen	282	7.473	2.198	58.595	7,63 %	5,95 %	8,09 %	6,34 %	1,26	< 0,001
C91	Lymphatische Leukämie	11	357	85	2.403	0,30 %	0,23 %	0,39 %	0,26 %	1,25	0,481
R43	Störungen des Geruchs- und Geschmackssinnes	18	469	137	3.447	0,49 %	0,37 %	0,51 %	0,37 %	1,25	0,371

M70	Krankheiten des Weichteilgewebes im Zusammenhang mit Beanspruchung, Überbeanspruchung und Druck	74	1.880	569	14.486	2,00 %	1,54 %	2,04 %	1,57 %	1,25	0,075
R25	Abnorme unwillkürliche Bewegungen	62	1.617	478	13.146	1,68 %	1,29 %	1,75 %	1,42 %	1,25	0,103
M76	Enthesopathien der unteren Extremität mit Ausnahme des Fußes	97	2.422	741	18.343	2,62 %	2,00 %	2,62 %	1,99 %	1,25	0,042
M80	Osteoporose mit pathologischer Fraktur	47	1.422	369	12.120	1,27 %	1,00 %	1,54 %	1,31 %	1,25	0,162
H19	Affektionen der Sklera und der Hornhaut bei anderenorts klassifizierten Krankheiten	24	720	186	5.136	0,65 %	0,50 %	0,78 %	0,56 %	1,25	0,314
F50	Essstörungen	26	632	200	4.893	0,70 %	0,54 %	0,68 %	0,53 %	1,25	0,295
J06	Akute Infektionen an mehreren oder nicht näher bezeichneten Lokalisationen der oberen Atemwege	1.085	25.696	8.817	207.915	29,35 %	23,85 %	27,82 %	22,51 %	1,24	< 0,001
N43	Hydrozele und Spermatozele	32	790	246	6.190	0,87 %	0,67 %	0,86 %	0,67 %	1,24	0,255
N39	Sonstige Krankheiten des Harnsystems	388	10.679	3.105	86.270	10,49 %	8,40 %	11,56 %	9,34 %	1,24	< 0,001
Z48	Andere Nachbehandlung	113	2.917	877	23.253	3,06 %	2,37 %	3,16 %	2,52 %	1,24	0,033

	nach chirurgischem Eingriff										
G44	Sonstige Kopfschmerzsyndrome	91	2.326	700	17.086	2,46 %	1,89 %	2,52 %	1,85 %	1,24	0,055
I47	Paroxysmale Tachykardie	64	1.688	498	13.367	1,73 %	1,35 %	1,83 %	1,45 %	1,24	0,108
Z90	Verlust von Organen, anderenorts nicht klassifiziert	224	6.161	1.785	48.885	6,06 %	4,83 %	6,67 %	5,29 %	1,24	0,004
K43	Hernia ventralis	25	664	194	5.223	0,68 %	0,52 %	0,72 %	0,57 %	1,24	0,314
Z98	Sonstige Zustände nach chirurgischem Eingriff	291	7.583	2.283	59.411	7,87 %	6,18 %	8,21 %	6,43 %	1,24	0,001
M67	Sonstige Krankheiten der Synovialis und der Sehnen	62	1.487	475	11.736	1,68 %	1,28 %	1,61 %	1,27 %	1,24	0,116
N62	Hypertrophie der Mamma [Brustdrüse]	26	661	201	5.094	0,70 %	0,54 %	0,72 %	0,55 %	1,24	0,310
Z37	Resultat der Entbindung	25	615	198	4.940	0,68 %	0,54 %	0,67 %	0,53 %	1,24	0,345
H25	Cataracta senilis	289	7.948	2.346	67.122	7,82 %	6,35 %	8,61 %	7,27 %	1,24	0,002
N64	Sonstige Krankheiten der Mamma [Brustdrüse]	132	3.312	1.044	25.967	3,57 %	2,82 %	3,59 %	2,81 %	1,23	0,030
I89	Sonstige nichtinfektiöse Krankheiten der Lymphgefäße und Lymphknoten	150	4.002	1.189	32.059	4,06 %	3,22 %	4,33 %	3,47 %	1,23	0,020
A49	Bakterielle Infektion nicht näher	35	935	273	7.169	0,95 %	0,74 %	1,01 %	0,78 %	1,23	0,252

	bezeichneter Lokalisation										
R05	Husten	232	5.672	1.809	45.339	6,28 %	4,89 %	6,14 %	4,91 %	1,23	0,004
J98	Sonstige Krankheiten der Atemwege	148	3.592	1.150	28.319	4,00 %	3,11 %	3,89 %	3,07 %	1,23	0,022
U99	Belegte und nicht belegte Schlüsselnummern U99.-!	305	8.205	2.426	65.429	8,25 %	6,56 %	8,88 %	7,08 %	1,23	0,001
B02	Zoster [Herpes zoster]	54	1.404	426	11.280	1,46 %	1,15 %	1,52 %	1,22 %	1,22	0,164
H65	Nichteitrige Otitis media	37	893	286	7.017	1,00 %	0,77 %	0,97 %	0,76 %	1,22	0,248
G54	Krankheiten von Nervenwurzeln und Nervenplexus	34	887	264	6.731	0,92 %	0,71 %	0,96 %	0,73 %	1,22	0,268
F51	Nichtorganische Schlafstörungen	85	2.154	668	17.260	2,30 %	1,81 %	2,33 %	1,87 %	1,22	0,090
D32	Gutartige Neubildung der Meningen	17	514	136	3.790	0,46 %	0,37 %	0,56 %	0,41 %	1,22	0,445
Z76	Personen, die das Gesundheitswesen aus sonstigen Gründen in Anspruch nehmen	112	2.784	882	22.614	3,03 %	2,39 %	3,02 %	2,45 %	1,22	0,057
R51	Kopfschmerz	247	5.951	1.949	47.615	6,68 %	5,27 %	6,44 %	5,16 %	1,22	0,005
I35	Nichtrheumatische Aortenklappenkrankheiten	139	4.317	1.132	33.903	3,76 %	3,06 %	4,67 %	3,67 %	1,22	0,040
Z11	Spezielle Verfahren zur Untersuchung auf infektiöse und parasitäre Krankheiten	299	8.108	2.406	65.469	8,09 %	6,51 %	8,78 %	7,09 %	1,21	0,003

R06	Störungen der Atmung	247	6.345	1.972	51.693	6,68 %	5,33 %	6,87 %	5,60 %	1,21	0,006
N85	Sonstige nichtentzündliche Krankheiten des Uterus, ausgenommen der Zervix	50	1.296	403	9.934	1,35 %	1,09 %	1,40 %	1,08 %	1,21	0,209
G47	Schlafstörungen	449	11.481	3.624	92.661	12,14 %	9,80 %	12,43 %	10,03 %	1,21	< 0,001
I70	Atherosklerose	261	7.520	2.141	60.943	7,06 %	5,79 %	8,14 %	6,60 %	1,21	0,008
K21	Gastroösophageale Refluxkrankheit	485	12.728	3.941	104.081	13,12 %	10,66 %	13,78 %	11,27 %	1,21	< 0,001
Z33	Schwangerschaftsfeststellung als Nebenbefund	11	263	87	2.173	0,30 %	0,24 %	0,28 %	0,24 %	1,21	0,564
T14	Verletzung an einer nicht näher bezeichneten Körperregion	292	7.485	2.335	59.863	7,90 %	6,32 %	8,10 %	6,48 %	1,20	0,004
Z34	Überwachung einer normalen Schwangerschaft	32	785	259	6.452	0,87 %	0,70 %	0,85 %	0,70 %	1,20	0,355
H69	Sonstige Krankheiten der Tuba auditiva	43	1.045	338	8.401	1,16 %	0,91 %	1,13 %	0,91 %	1,20	0,260
I80	Thrombose, Phlebitis und Thrombophlebitis	64	1.683	513	13.543	1,73 %	1,39 %	1,82 %	1,47 %	1,20	0,169
F43	Reaktionen auf schwere Belastungen und Anpassungsstörungen	459	11.272	3.723	91.125	12,42 %	10,07 %	12,21 %	9,87 %	1,20	< 0,001
H35	Sonstige Affektionen der Netzhaut	277	8.055	2.311	68.532	7,49 %	6,25 %	8,72 %	7,42 %	1,20	0,010

M93	Sonstige Osteochondropathien	68	1.818	545	14.136	1,84 %	1,47 %	1,97 %	1,53 %	1,20	0,169
K07	Dentofaziale Anomalien [einschließlich fehlerhafter Okklusion]	29	706	233	5.731	0,78 %	0,63 %	0,76 %	0,62 %	1,19	0,370
Z60	Kontaktanlässe mit Bezug auf die soziale Umgebung	21	504	168	4.841	0,57 %	0,45 %	0,55 %	0,52 %	1,19	0,450
C50	Bösartige Neubildung der Brustdrüse [Mamma]	77	2.210	633	18.280	2,08 %	1,71 %	2,39 %	1,98 %	1,19	0,161
I31	Sonstige Krankheiten des Perikards	10	241	81	2.154	0,27 %	0,22 %	0,26 %	0,23 %	1,19	0,606
F33	Rezidivierende depressive Störung	290	7.066	2.350	58.148	7,84 %	6,36 %	7,65 %	6,30 %	1,19	0,009
F62	Andauernde Persönlichkeitsänderungen, nicht Folge einer Schädigung oder Krankheit des Gehirns	11	272	89	2.259	0,30 %	0,24 %	0,29 %	0,24 %	1,18	0,597
Z39	Postpartale Betreuung und Untersuchung der Mutter	18	444	147	3.632	0,49 %	0,40 %	0,48 %	0,39 %	1,18	0,512
R11	Übelkeit und Erbrechen	140	3.593	1.132	29.426	3,79 %	3,06 %	3,89 %	3,19 %	1,18	0,065
H60	Otitis externa	151	3.933	1.228	32.012	4,08 %	3,32 %	4,26 %	3,47 %	1,18	0,058
R42	Schwindel und Taumel	312	8.784	2.594	73.714	8,44 %	7,02 %	9,51 %	7,98 %	1,18	0,009

C44	Sonstige bösartige Neubildungen der Haut	126	3.660	1.047	30.436	3,41 %	2,83 %	3,96 %	3,30 %	1,18	0,091
D47	Sonstige Neubildungen unsicheren oder unbekannten Verhaltens des lymphatischen, blutbildenden und verwandten Gewebes	23	668	189	5.353	0,62 %	0,51 %	0,72 %	0,58 %	1,18	0,457
H50	Sonstiger Strabismus	109	2.854	888	23.562	2,95 %	2,40 %	3,09 %	2,55 %	1,18	0,111
I99	Sonstige und nicht näher bezeichnete Krankheiten des Kreislaufsystems	55	1.451	448	11.533	1,49 %	1,21 %	1,57 %	1,25 %	1,18	0,256
Z46	Versorgen mit und Anpassen von anderen medizinischen Geräten oder Hilfsmitteln	164	4.892	1.365	39.059	4,44 %	3,69 %	5,30 %	4,23 %	1,18	0,060
N20	Nieren- und Ureterstein	101	2.555	824	21.243	2,73 %	2,23 %	2,77 %	2,30 %	1,17	0,137
S02	Fraktur des Schädels und der Gesichtsschädelknochen	10	235	81	2.169	0,27 %	0,22 %	0,25 %	0,23 %	1,17	0,635
Z85	Bösartige Neubildung in der Eigenanamnese	61	1.704	508	14.828	1,65 %	1,37 %	1,85 %	1,61 %	1,17	0,261
J01	Akute Sinusitis	93	2.187	756	17.997	2,52 %	2,04 %	2,37 %	1,95 %	1,17	0,167
N35	Harnröhrenstriktur	16	382	131	3.646	0,43 %	0,35 %	0,41 %	0,39 %	1,17	0,560
K70	Alkoholische Leberkrankheit	24	574	196	4.810	0,65 %	0,53 %	0,62 %	0,52 %	1,17	0,478

I36	Nichtrheumatische Trikuspidalklappenkrankheiten	30	882	250	7.682	0,81 %	0,68 %	0,96 %	0,83 %	1,16	0,435
Z92	Medizinische Behandlung in der Eigenanamnese	375	10.592	3.236	93.452	10,14 %	8,75 %	11,47 %	10,12 %	1,16	0,016
M53	Sonstige Krankheiten der Wirbelsäule und des Rückens, anderenorts nicht klassifiziert	390	9.962	3.268	82.906	10,55 %	8,84 %	10,79 %	8,98 %	1,16	0,009
Z87	Andere Krankheiten oder Zustände in der Eigenanamnese	25	653	207	5.477	0,68 %	0,56 %	0,71 %	0,59 %	1,16	0,481
I71	Aortenaneurysma und -dissektion	61	1.750	510	14.022	1,65 %	1,38 %	1,89 %	1,52 %	1,16	0,281
S50	Oberflächliche Verletzung des Unterarmes	17	416	139	3.896	0,46 %	0,38 %	0,45 %	0,42 %	1,16	0,565
C61	Bösartige Neubildung der Prostata	64	1.712	544	14.904	1,73 %	1,47 %	1,85 %	1,61 %	1,16	0,296
H11	Sonstige Affektionen der Konjunktiva	57	1.558	476	12.607	1,54 %	1,29 %	1,69 %	1,37 %	1,16	0,303
Z38	Lebendgeborene nach dem Geburtsort	20	492	168	4.201	0,54 %	0,45 %	0,53 %	0,45 %	1,15	0,563
G53	Krankheiten der Hirnnerven bei anderenorts klassifizierten Krankheiten	12	313	101	2.731	0,32 %	0,27 %	0,34 %	0,30 %	1,15	0,642
N91	Ausgebliebene, zu schwache oder zu seltene Menstruation	59	1.382	499	11.692	1,60 %	1,35 %	1,50 %	1,27 %	1,15	0,323

R92	Abnorme Befunde bei der bildgebenden Diagnostik der Mamma [Brustdrüse]	11	313	92	2.422	0,30 %	0,25 %	0,34 %	0,26 %	1,15	0,662
U09	Post-COVID-19-Zustand	43	1.055	355	8.543	1,16 %	0,96 %	1,14 %	0,93 %	1,15	0,391
R65	Systemisches inflammatorisches Response-Syndrom [SIRS]	16	458	134	3.828	0,43 %	0,36 %	0,50 %	0,41 %	1,15	0,603
F99	Psychische Störung ohne nähere Angabe	15	368	126	3.239	0,41 %	0,34 %	0,40 %	0,35 %	1,15	0,616
J03	Akute Tonsillitis	109	2.586	894	21.194	2,95 %	2,42 %	2,80 %	2,29 %	1,15	0,189
H40	Glaukom	225	6.357	1.924	54.458	6,09 %	5,20 %	6,88 %	5,90 %	1,15	0,066
K92	Sonstige Krankheiten des Verdauungssystems	48	1.264	401	10.452	1,30 %	1,08 %	1,37 %	1,13 %	1,15	0,375
I65	Verschluss und Stenose präzerebraler Arterien ohne resultierenden Hirninfarkt	156	4.414	1.330	37.332	4,22 %	3,60 %	4,78 %	4,04 %	1,15	0,126
B49	Nicht näher bezeichnete Mykose	22	512	181	4.730	0,60 %	0,49 %	0,55 %	0,51 %	1,14	0,553
E11	Diabetes mellitus, Typ 2	566	14.909	4.933	135.265	15,31 %	13,34 %	16,14 %	14,65 %	1,14	0,010
K12	Stomatitis und verwandte Krankheiten	12	309	101	2.557	0,32 %	0,27 %	0,33 %	0,28 %	1,14	0,665
R50	Fieber sonstiger und unbekannter Ursache	56	1.391	466	11.429	1,51 %	1,26 %	1,51 %	1,24 %	1,14	0,355
E66	Adipositas	628	15.540	5.391	135.812	16,99 %	14,58 %	16,83 %	14,71 %	1,14	0,005

I44	Atrioventrikulärer Block und Linksschenkelblock	94	2.680	805	23.857	2,54 %	2,18 %	2,90 %	2,58 %	1,14	0,247
E83	Störungen des Mineralstoffwechsels	38	992	322	8.754	1,03 %	0,87 %	1,07 %	0,95 %	1,14	0,452
H34	Netzhautgefäßverschluss	17	531	145	4.211	0,46 %	0,39 %	0,57 %	0,46 %	1,14	0,621
N83	Nichtentzündliche Krankheiten des Ovars, der Tuba uterina und des Lig. latum uteri	60	1.429	512	12.364	1,62 %	1,38 %	1,55 %	1,34 %	1,14	0,362
E88	Sonstige Stoffwechselstörungen	117	2.974	991	25.762	3,16 %	2,68 %	3,22 %	2,79 %	1,13	0,210
S42	Fraktur im Bereich der Schulter und des Oberarmes	27	707	227	6.446	0,73 %	0,61 %	0,77 %	0,70 %	1,13	0,543
Q74	Sonstige angeborene Fehlbildungen der Extremität(en)	15	385	125	2.947	0,41 %	0,34 %	0,42 %	0,32 %	1,13	0,655
R52	Schmerz, anderenorts nicht klassifiziert	429	11.556	3.716	101.931	11,60 %	10,05 %	12,51 %	11,04 %	1,13	0,029
R91	Abnorme Befunde bei der bildgebenden Diagnostik der Lunge	20	497	170	4.595	0,54 %	0,46 %	0,54 %	0,50 %	1,13	0,612
B34	Viruskrankheit nicht näher bezeichneter Lokalisation	164	3.839	1.366	32.151	4,44 %	3,69 %	4,16 %	3,48 %	1,13	0,157
R13	Dysphagie	58	1.519	493	13.499	1,57 %	1,33 %	1,65 %	1,46 %	1,12	0,401

E71	Störungen des Stoffwechsels verzweigter Aminosäuren und des Fettsäurestoffwechsels	11	253	93	2.575	0,30 %	0,25 %	0,27 %	0,28 %	1,12	0,716
H47	Sonstige Affektionen des N. opticus [II. Hirnnerv] und der Sehbahn	105	2.932	903	25.107	2,84 %	2,44 %	3,18 %	2,72 %	1,12	0,269
F06	Andere psychische Störungen aufgrund einer Schädigung oder Funktionsstörung des Gehirns oder einer körperlichen Krankheit	54	1.583	467	13.872	1,46 %	1,26 %	1,71 %	1,50 %	1,12	0,432
S82	Fraktur des Unterschenkels, einschließlich des oberen Sprunggelenkes	31	856	264	6.967	0,84 %	0,71 %	0,93 %	0,75 %	1,12	0,550
C77	Sekundäre und nicht näher bezeichnete bösartige Neubildung der Lymphknoten	22	601	190	4.997	0,60 %	0,51 %	0,65 %	0,54 %	1,12	0,618
S90	Oberflächliche Verletzung der Knöchelregion und des Fußes	26	627	220	5.465	0,70 %	0,60 %	0,68 %	0,59 %	1,12	0,596
R55	Synkope und Kollaps	48	1.403	415	11.642	1,30 %	1,12 %	1,52 %	1,26 %	1,11	0,500
G31	Sonstige degenerative Krankheiten des	16	518	139	4.302	0,43 %	0,38 %	0,56 %	0,47 %	1,11	0,696

	Nervensystems, anderenorts nicht klassifiziert										
Z45	Anpassung und Handhabung eines implantierten medizinischen Gerätes	30	804	263	7.519	0,81 %	0,71 %	0,87 %	0,81 %	1,11	0,596
T79	Bestimmte Frühkomplikationen eines Traumas, anderenorts nicht klassifiziert	51	1.276	442	11.736	1,38 %	1,20 %	1,38 %	1,27 %	1,11	0,501
B99	Sonstige und nicht näher bezeichnete Infektionskrankheiten	100	2.387	858	20.991	2,70 %	2,32 %	2,58 %	2,27 %	1,10	0,364
I42	Kardiomyopathie	44	1.179	382	10.455	1,19 %	1,03 %	1,28 %	1,13 %	1,10	0,546
E46	Nicht näher bezeichnete Energie- und Eiweißmangelernährung	11	339	96	3.011	0,30 %	0,26 %	0,37 %	0,33 %	1,10	0,763
S20	Oberflächliche Verletzung des Thorax	43	1.052	370	9.648	1,16 %	1,00 %	1,14 %	1,04 %	1,10	0,556
Z56	Kontaktanlässe mit Bezug auf das Berufsleben	26	597	225	5.242	0,70 %	0,61 %	0,65 %	0,57 %	1,10	0,653
L02	Hautabszess, Furunkel und Karbunkel	50	1.195	431	10.629	1,35 %	1,17 %	1,29 %	1,15 %	1,09	0,549
R89	Abnorme Befunde in Untersuchungsmaterialien aus anderen Körperorganen, -	12	324	105	2.577	0,32 %	0,28 %	0,35 %	0,28 %	1,09	0,769

	systemen und -geweben										
Z43	Versorgung künstlicher Körperöffnungen	16	425	140	3.899	0,43 %	0,38 %	0,46 %	0,42 %	1,09	0,738
F90	Hyperkinetische Störungen	28	665	236	5.590	0,76 %	0,64 %	0,72 %	0,61 %	1,09	0,666
I07	Rheumatische Trikuspidalklappenkrankheiten	66	1.894	591	17.517	1,79 %	1,60 %	2,05 %	1,90 %	1,09	0,518
S00	Oberflächliche Verletzung des Kopfes	41	1.163	359	10.098	1,11 %	0,97 %	1,26 %	1,09 %	1,09	0,613
S62	Fraktur im Bereich des Handgelenkes und der Hand	20	481	173	4.429	0,54 %	0,47 %	0,52 %	0,48 %	1,09	0,729
N63	Nicht näher bezeichnete Knoten in der Mamma [Brustdrüse]	10	252	88	2.125	0,27 %	0,24 %	0,27 %	0,23 %	1,08	0,812
R29	Sonstige Symptome, die das Nervensystem und das Muskel-Skelett-System betreffen	159	4.849	1.440	45.720	4,30 %	3,90 %	5,25 %	4,95 %	1,08	0,380
R49	Störungen der Stimme	45	1.253	399	10.356	1,22 %	1,08 %	1,36 %	1,12 %	1,08	0,621
R21	Hautausschlag und sonstige unspezifische Hauteruptionen	29	739	256	6.639	0,78 %	0,69 %	0,80 %	0,72 %	1,08	0,692
S81	Offene Wunde des Unterschenkels	12	316	106	2.914	0,32 %	0,29 %	0,34 %	0,32 %	1,08	0,800
D68	Sonstige Koagulopathien	71	1.909	633	16.893	1,92 %	1,71 %	2,07 %	1,83 %	1,08	0,544
R14	Flatulenz und verwandte Zustände	52	1.370	462	11.964	1,41 %	1,25 %	1,48 %	1,30 %	1,08	0,601

I11	Hypertensive Herzkrankheit	196	5.606	1.771	50.729	5,30 %	4,79 %	6,07 %	5,49 %	1,08	0,348
R39	Sonstige Symptome, die das Harnsystem betreffen	81	2.116	725	19.315	2,19 %	1,96 %	2,29 %	2,09 %	1,08	0,543
Z29	Notwendigkeit von anderen prophylaktischen Maßnahmen	58	1.606	522	14.524	1,57 %	1,41 %	1,74 %	1,57 %	1,07	0,623
I10	Essentielle (primäre) Hypertonie	1.656	43.760	15.457	412.605	44,79 %	41,81 %	47,38 %	44,68 %	1,07	0,100
S06	Intrakranielle Verletzung	23	610	205	5.947	0,62 %	0,55 %	0,66 %	0,64 %	1,07	0,765
D41	Neubildung unsicheren oder unbekannten Verhaltens der Harnorgane	15	443	136	3.794	0,41 %	0,37 %	0,48 %	0,41 %	1,07	0,815
J42	Nicht näher bezeichnete chronische Bronchitis	42	1.104	378	9.803	1,14 %	1,02 %	1,20 %	1,06 %	1,06	0,707
R26	Störungen des Ganges und der Mobilität	230	7.316	2.141	68.655	6,22 %	5,79 %	7,92 %	7,43 %	1,06	0,449
R87	Abnorme Befunde in Untersuchungsmaterialien aus den weiblichen Genitalorganen	27	650	245	5.856	0,73 %	0,66 %	0,70 %	0,63 %	1,06	0,772
T84	Komplikationen durch orthopädische Endoprothesen, Implantate oder Transplantate	22	637	201	5.810	0,60 %	0,54 %	0,69 %	0,63 %	1,06	0,797
R60	Ödem, anderenorts nicht klassifiziert	116	3.353	1.070	30.774	3,14 %	2,89 %	3,63 %	3,33 %	1,05	0,614

Z86	Bestimmte andere Krankheiten in der Eigenanamnese	75	1.941	688	18.671	2,03 %	1,86 %	2,10 %	2,02 %	1,04	0,726
Z95	Vorhandensein von kardialen oder vaskulären Implantaten oder Transplantaten	200	5.514	1.867	52.893	5,41 %	5,05 %	5,97 %	5,73 %	1,04	0,593
R54	Senilität	130	4.293	1.234	42.119	3,52 %	3,34 %	4,65 %	4,56 %	1,04	0,696
U51	Kognitive Funktionseinschränk ung	57	1.778	532	16.245	1,54 %	1,44 %	1,93 %	1,76 %	1,04	0,776
S22	Fraktur der Rippe(n), des Sternums und der Brustwirbelsäule	30	826	278	8.033	0,81 %	0,75 %	0,89 %	0,87 %	1,04	0,851
H33	Netzhautablösung und Netzhautriss	25	631	232	6.211	0,68 %	0,63 %	0,68 %	0,67 %	1,04	0,870
Z20	Kontakt mit und Exposition gegenüber übertragbaren Krankheiten	23	572	209	5.070	0,62 %	0,57 %	0,62 %	0,55 %	1,03	0,883
I21	Akuter Myokardinfarkt	39	1.050	363	9.943	1,05 %	0,98 %	1,14 %	1,08 %	1,03	0,851
I50	Herzinsuffizienz	234	6.767	2.220	66.635	6,33 %	6,00 %	7,33 %	7,22 %	1,03	0,710
S83	Luxation, Verstauchung und Zerrung des Kniegelenkes und von Bändern des Kniegelenkes	63	1.479	573	13.742	1,70 %	1,55 %	1,60 %	1,49 %	1,02	0,854
H31	Sonstige Affektionen der Aderhaut	15	405	141	4.112	0,41 %	0,38 %	0,44 %	0,45 %	1,02	0,934
K52	Sonstige nichtinfektiöse	96	2.337	885	22.168	2,60 %	2,39 %	2,53 %	2,40 %	1,02	0,849

	Gastroenteritis und Kolitis										
I25	Chronische ischämische Herzkrankheit	350	9.801	3.336	94.401	9,47 %	9,02 %	10,61 %	10,22 %	1,02	0,750
N19	Nicht näher bezeichnete Niereninsuffizienz	73	2.118	695	21.116	1,97 %	1,88 %	2,29 %	2,29 %	1,02	0,874
C64	Bösartige Neubildung der Niere, ausgenommen Nierenbecken	11	298	103	2.913	0,30 %	0,28 %	0,32 %	0,32 %	1,02	0,956
R18	Aszites	11	283	105	2.795	0,30 %	0,28 %	0,31 %	0,30 %	1,02	0,962
S30	Oberflächliche Verletzung des Abdomens, der Lumbosakralgegend und des Beckens	22	581	209	5.891	0,60 %	0,57 %	0,63 %	0,64 %	1,01	0,955
S52	Fraktur des Unterarmes	27	734	256	7.181	0,73 %	0,69 %	0,79 %	0,78 %	1,01	0,952
I48	Vorhofflimmern und Vorhofflattern	208	6.263	2.014	61.551	5,63 %	5,45 %	6,78 %	6,66 %	1,01	0,936
H66	Eitrige und nicht näher bezeichnete Otitis media	48	1.145	450	11.116	1,30 %	1,22 %	1,24 %	1,20 %	1,00	0,986
E13	Sonstiger näher bezeichneter Diabetes mellitus	17	432	164	4.469	0,46 %	0,44 %	0,47 %	0,48 %	1,00	0,999
F13	Psychische und Verhaltensstörungen durch Sedativa oder Hypnotika	11	285	105	2.964	0,30 %	0,28 %	0,31 %	0,32 %	1,00	0,999
H68	Entzündung und Verschluss der Tuba auditiva	23	604	218	5.346	0,62 %	0,59 %	0,65 %	0,58 %	1,00	0,999

U50	Motorische Funktionseinschränkung	147	4.391	1.427	43.588	3,98 %	3,86 %	4,75 %	4,72 %	1,00	0,987
S60	Oberflächliche Verletzung des Handgelenkes und der Hand	35	874	331	8.441	0,95 %	0,90 %	0,95 %	0,91 %	1,00	0,979
I26	Lungenembolie	23	646	222	6.052	0,62 %	0,60 %	0,70 %	0,66 %	0,99	0,975
E87	Sonstige Störungen des Wasser- und Elektrolythaushaltes sowie des Säure-Basen-Gleichgewichts	118	3.429	1.146	33.899	3,19 %	3,10 %	3,71 %	3,67 %	0,99	0,939
B96	Sonstige näher bezeichnete Bakterien als Ursache von Krankheiten, die in anderen Kapiteln klassifiziert sind	58	1.721	566	16.749	1,57 %	1,53 %	1,86 %	1,81 %	0,99	0,936
T16	Fremdkörper im Ohr	23	609	223	5.863	0,62 %	0,60 %	0,66 %	0,63 %	0,99	0,959
I88	Unspezifische Lymphadenitis	15	373	142	3.513	0,41 %	0,38 %	0,40 %	0,38 %	0,99	0,965
N94	Schmerz und andere Zustände im Zusammenhang mit den weiblichen Genitalorganen und dem Menstruationszyklus	128	3.079	1.246	29.397	3,46 %	3,37 %	3,33 %	3,18 %	0,98	0,859
N99	Krankheiten des Urogenitalsystems nach medizinischen Maßnahmen,	13	334	129	3.511	0,35 %	0,35 %	0,36 %	0,38 %	0,98	0,948

	anderenorts nicht klassifiziert										
J40	Bronchitis, nicht als akut oder chronisch bezeichnet	123	3.103	1.195	29.861	3,33 %	3,23 %	3,36 %	3,23 %	0,98	0,831
R09	Sonstige Symptome, die das Kreislaufsystem und das Atmungssystem betreffen	10	254	99	2.757	0,27 %	0,27 %	0,28 %	0,30 %	0,98	0,950
Z99	Abhängigkeit (langzeitig) von unterstützenden Apparaten, medizinischen Geräten oder Hilfsmitteln, anderenorts nicht klassifiziert	33	922	327	8.819	0,89 %	0,88 %	1,00 %	0,95 %	0,98	0,893
T81	Komplikationen bei Eingriffen, anderenorts nicht klassifiziert	51	1.376	504	13.494	1,38 %	1,36 %	1,49 %	1,46 %	0,98	0,866
H17	Hornhautnarben und -trübungen	23	610	226	6.252	0,62 %	0,61 %	0,66 %	0,68 %	0,98	0,909
K26	Ulcus duodeni	14	384	139	3.769	0,38 %	0,38 %	0,42 %	0,41 %	0,97	0,914
J41	Einfache und schleimig-eitrige chronische Bronchitis	25	644	244	6.170	0,68 %	0,66 %	0,70 %	0,67 %	0,97	0,878
R93	Abnorme Befunde bei der bildgebenden Diagnostik sonstiger Körperstrukturen	42	1.080	413	10.423	1,14 %	1,12 %	1,17 %	1,13 %	0,97	0,836

K50	Crohn-Krankheit [Enteritis regionalis] [Morbus Crohn]	20	515	197	4.775	0,54 %	0,53 %	0,56 %	0,52 %	0,96	0,877
D35	Gutartige Neubildung sonstiger und nicht näher bezeichneter endokriner Drüsen	17	406	170	4.499	0,46 %	0,46 %	0,44 %	0,49 %	0,96	0,882
K74	Fibrose und Zirrhose der Leber	20	475	199	5.143	0,54 %	0,54 %	0,51 %	0,56 %	0,96	0,852
N13	Obstruktive Uropathie und Refluxuropathie	28	741	280	7.495	0,76 %	0,76 %	0,80 %	0,81 %	0,95	0,817
J43	Emphysem	46	1.271	461	12.439	1,24 %	1,25 %	1,38 %	1,35 %	0,95	0,765
T83	Komplikationen durch Prothesen, Implantate oder Transplantate im Urogenitaltrakt	13	344	132	3.754	0,35 %	0,36 %	0,37 %	0,41 %	0,95	0,866
J18	Pneumonie, Erreger nicht näher bezeichnet	48	1.325	486	13.500	1,30 %	1,31 %	1,43 %	1,46 %	0,95	0,739
L89	Dekubitalgeschwür und Druckzone	32	939	327	10.349	0,87 %	0,88 %	1,02 %	1,12 %	0,95	0,770
I64	Schlaganfall, nicht als Blutung oder Infarkt bezeichnet	25	675	256	7.524	0,68 %	0,69 %	0,73 %	0,81 %	0,95	0,792
E86	Volumenmangel	55	1.761	561	17.552	1,49 %	1,52 %	1,91 %	1,90 %	0,94	0,689
I69	Folgen einer zerebrovaskulären Krankheit	87	2.478	891	25.394	2,35 %	2,41 %	2,68 %	2,75 %	0,94	0,608
G40	Epilepsie	52	1.428	521	13.465	1,41 %	1,41 %	1,55 %	1,46 %	0,94	0,681
S01	Offene Wunde des Kopfes	28	821	283	8.169	0,76 %	0,77 %	0,89 %	0,88 %	0,94	0,754
J22	Akute Infektion der unteren Atemwege,	21	578	211	5.406	0,57 %	0,57 %	0,63 %	0,59 %	0,94	0,782

	nicht näher bezeichnet										
K11	Krankheiten der Speicheldrüsen	12	305	123	3.203	0,32 %	0,33 %	0,33 %	0,35 %	0,94	0,832
I97	Kreislaufkomplikationen nach medizinischen Maßnahmen, anderenorts nicht klassifiziert	14	359	144	3.956	0,38 %	0,39 %	0,39 %	0,43 %	0,94	0,815
A09	Sonstige und nicht näher bezeichnete Gastroenteritis und Kolitis infektiösen und nicht näher bezeichneten Ursprungs	244	5.882	2.416	58.282	6,60 %	6,54 %	6,37 %	6,31 %	0,94	0,342
S93	Luxation, Verstauchung und Zerrung der Gelenke und Bänder in Höhe des oberen Sprunggelenkes und des Fußes	58	1.391	583	14.112	1,57 %	1,58 %	1,51 %	1,53 %	0,93	0,612
A08	Virusbedingte und sonstige näher bezeichnete Darminfektionen	23	602	231	5.662	0,62 %	0,62 %	0,65 %	0,61 %	0,93	0,743
G81	Hemiparese und Hemiplegie	40	1.133	417	11.864	1,08 %	1,13 %	1,23 %	1,28 %	0,92	0,616
R32	Nicht näher bezeichnete Harninkontinenz	138	4.342	1.445	46.077	3,73 %	3,91 %	4,70 %	4,99 %	0,92	0,375
T01	Offene Wunden mit Beteiligung mehrerer Körperregionen	17	478	177	4.710	0,46 %	0,48 %	0,52 %	0,51 %	0,91	0,707

I12	Hypertensive Nierenkrankheit	20	596	217	6.428	0,54 %	0,59 %	0,65 %	0,70 %	0,90	0,654
J91	Pleuraerguss bei anderenorts klassifizierten Krankheiten	24	716	258	8.117	0,65 %	0,70 %	0,77 %	0,88 %	0,90	0,625
D63	Anämie bei chronischen, anderenorts klassifizierten Krankheiten	16	417	173	4.994	0,43 %	0,47 %	0,45 %	0,54 %	0,90	0,674
C79	Sekundäre bösartige Neubildung an sonstigen und nicht näher bezeichneten Lokalisationen	19	535	206	5.712	0,51 %	0,56 %	0,58 %	0,62 %	0,89	0,635
S32	Fraktur der Lendenwirbelsäule und des Beckens	24	750	261	8.173	0,65 %	0,71 %	0,81 %	0,88 %	0,89	0,583
F10	Psychische und Verhaltensstörungen durch Alkohol	70	1.665	738	17.448	1,89 %	2,00 %	1,80 %	1,89 %	0,89	0,342
S61	Offene Wunde des Handgelenkes und der Hand	22	549	237	5.815	0,60 %	0,64 %	0,59 %	0,63 %	0,88	0,571
J44	Sonstige chronische obstruktive Lungenkrankheit	197	5.315	2.132	56.619	5,33 %	5,77 %	5,76 %	6,13 %	0,87	0,084
J15	Pneumonie durch Bakterien, anderenorts nicht klassifiziert	12	383	132	3.903	0,32 %	0,36 %	0,41 %	0,42 %	0,87	0,656
Z02	Untersuchung und Konsultation aus administrativen Gründen	15	375	160	4.074	0,41 %	0,43 %	0,41 %	0,44 %	0,87	0,607

F31	Bipolare affektive Störung	14	341	151	3.698	0,38 %	0,41 %	0,37 %	0,40 %	0,87	0,618
R15	Stuhlinkontinenz	36	1.058	401	12.610	0,97 %	1,08 %	1,15 %	1,37 %	0,86	0,410
J96	Respiratorische Insuffizienz, anderenorts nicht klassifiziert	65	1.865	723	20.943	1,76 %	1,96 %	2,02 %	2,27 %	0,86	0,259
L97	Ulcus cruris, anderenorts nicht klassifiziert	15	422	171	5.046	0,41 %	0,46 %	0,46 %	0,55 %	0,85	0,535
F17	Psychische und Verhaltensstörungen durch Tabak	270	6.439	2.979	71.172	7,30 %	8,06 %	6,97 %	7,71 %	0,84	0,011
C78	Sekundäre bösartige Neubildung der Atmungs- und Verdauungsorgane	17	476	197	5.461	0,46 %	0,53 %	0,52 %	0,59 %	0,84	0,485
Z74	Probleme mit Bezug auf Pflegebedürftigkeit	87	2.777	997	31.547	2,35 %	2,70 %	3,01 %	3,42 %	0,83	0,122
Z22	Keimträger von Infektionskrankheiten	28	712	322	8.481	0,76 %	0,87 %	0,77 %	0,92 %	0,83	0,351
F01	Vaskuläre Demenz	19	689	223	7.748	0,51 %	0,60 %	0,75 %	0,84 %	0,83	0,437
S51	Offene Wunde des Unterarmes	10	286	116	3.324	0,27 %	0,31 %	0,31 %	0,36 %	0,83	0,562
R47	Sprech- und Sprachstörungen, anderenorts nicht klassifiziert	31	887	361	10.254	0,84 %	0,98 %	0,96 %	1,11 %	0,82	0,307
I27	Sonstige pulmonale Herzkrankheiten	26	759	306	9.458	0,70 %	0,83 %	0,82 %	1,02 %	0,82	0,346
G30	Alzheimer-Krankheit	22	744	261	9.367	0,60 %	0,71 %	0,81 %	1,01 %	0,82	0,390
G82	Paraparese und Paraplegie,	13	341	151	3.965	0,35 %	0,41 %	0,37 %	0,43 %	0,82	0,488

	Tetraparese und Tetraplegie										
G35	Multiple Sklerose [Encephalomyelitis disseminata]	18	422	208	4.941	0,49 %	0,56 %	0,46 %	0,54 %	0,82	0,412
S72	Fraktur des Femurs	14	428	166	5.316	0,38 %	0,45 %	0,46 %	0,58 %	0,82	0,467
R40	Somnolenz, Sopor und Koma	10	243	118	3.618	0,27 %	0,32 %	0,26 %	0,39 %	0,81	0,535
I20	Angina pectoris	48	1.275	567	15.390	1,30 %	1,53 %	1,38 %	1,67 %	0,81	0,173
K08	Sonstige Krankheiten der Zähne und des Zahnhalteapparates	10	256	118	2.858	0,27 %	0,32 %	0,28 %	0,31 %	0,81	0,515
F00	Demenz bei Alzheimer-Krankheit	22	760	269	9.677	0,60 %	0,73 %	0,82 %	1,05 %	0,80	0,315
U69	Sonstige sekundäre Schlüsselnummern für besondere Zwecke	34	955	411	11.836	0,92 %	1,11 %	1,03 %	1,28 %	0,79	0,193
J38	Krankheiten der Stimmlippen und des Kehlkopfes, anderenorts nicht klassifiziert	13	330	157	4.063	0,35 %	0,42 %	0,36 %	0,44 %	0,79	0,406
Z50	Rehabilitationsmaßnahmen	14	348	171	4.797	0,38 %	0,46 %	0,38 %	0,52 %	0,78	0,374
J37	Chronische Laryngitis und Laryngotracheitis	17	462	215	5.541	0,46 %	0,58 %	0,50 %	0,60 %	0,75	0,261
N17	Akutes Nierenversagen	26	725	332	9.950	0,70 %	0,90 %	0,78 %	1,08 %	0,75	0,167
D62	Akute Blutungsanämie	19	543	245	7.139	0,51 %	0,66 %	0,59 %	0,77 %	0,75	0,232
I63	Hirninfarkt	35	996	453	12.893	0,95 %	1,23 %	1,08 %	1,40 %	0,75	0,096
T89	Sonstige näher bezeichnete	20	535	259	7.205	0,54 %	0,70 %	0,58 %	0,78 %	0,74	0,199

	Komplikationen eines Traumas										
C67	Bösartige Neubildung der Harnblase	14	414	187	5.299	0,38 %	0,51 %	0,45 %	0,57 %	0,72	0,245
G83	Sonstige Lähmungssyndrome	15	374	198	5.326	0,41 %	0,54 %	0,41 %	0,58 %	0,72	0,216
K25	Ulcus ventriculi	13	329	177	4.812	0,35 %	0,48 %	0,36 %	0,52 %	0,70	0,224
G20	Primäres Parkinson-Syndrom	16	482	222	6.567	0,43 %	0,60 %	0,52 %	0,71 %	0,70	0,171
S70	Oberflächliche Verletzung der Hüfte und des Oberschenkels	10	260	139	4.165	0,27 %	0,38 %	0,28 %	0,45 %	0,69	0,251
C34	Bösartige Neubildung der Bronchien und der Lunge	10	286	143	3.929	0,27 %	0,39 %	0,31 %	0,43 %	0,67	0,226
Z93	Vorhandensein einer künstlichen Körperöffnung	12	330	174	4.845	0,32 %	0,47 %	0,36 %	0,52 %	0,66	0,169
F07	Persönlichkeits- und Verhaltensstörung aufgrund einer Krankheit, Schädigung oder Funktionsstörung des Gehirns	10	300	145	4.290	0,27 %	0,39 %	0,32 %	0,46 %	0,66	0,203
G51	Krankheiten des N. facialis [VII. Hirnnerv]	11	325	164	4.392	0,30 %	0,44 %	0,35 %	0,48 %	0,64	0,146
F03	Nicht näher bezeichnete Demenz	31	1.059	573	20.302	0,84 %	1,55 %	1,15 %	2,20 %	0,50	< 0,001

Online-Tabelle 7 – Übersicht der Odds Ratios für Begleiterkrankungen nach ICD-10 bei Vitiligo-Patient*innen in Deutschland (2023)

Odds Ratios					
**ICD-10**	**Beschreibung**	**OR**	**Untere Grenze des KI**	**Obere Grenze des KI**	**p-Wert**
E31	Polyglanduläre Dysfunktion	27,79	13,02	59,32	< 0,001
L94	Sonstige lokalisierte Krankheiten des Bindegewebes	12,02	7,10	20,37	< 0,001
E06	Thyreoiditis	6,11	5,52	6,75	< 0,001
L63	Alopecia areata	5,59	3,97	7,86	< 0,001
E27	Sonstige Krankheiten der Nebenniere	5,01	3,09	8,15	< 0,001
E10	Diabetes mellitus, Typ 1	4,54	3,81	5,42	< 0,001
M34	Systemische Sklerose	4,30	2,38	7,75	< 0,001
E16	Sonstige Störungen der inneren Sekretion des Pankreas	4,01	2,60	6,20	< 0,001
M66	Spontanruptur der Synovialis und von Sehnen	3,89	1,93	7,85	< 0,001
D51	Vitamin-B12-Mangelanämie	3,68	2,86	4,73	< 0,001
D44	Neubildung unsicheren oder unbekannten Verhaltens der endokrinen Drüsen	3,60	2,35	5,53	< 0,001
L93	Lupus erythematodes	3,46	2,01	5,95	< 0,001
B16	Akute Virushepatitis B	3,45	1,73	6,88	< 0,001
L56	Sonstige akute Hautveränderungen durch Ultraviolettstrahlen	3,42	2,34	4,98	< 0,001
M32	Systemischer Lupus erythematodes	3,33	1,68	6,61	< 0,001
D86	Sarkoidose	3,16	2,17	4,59	< 0,001
L90	Atrophische Hautkrankheiten	3,11	2,53	3,83	< 0,001
H06	Affektionen des Tränenapparates und der Orbita bei anderenorts klassifizierten Krankheiten	2,94	1,50	5,79	0,002
N11	Chronische tubulointerstitielle Nephritis	2,92	1,53	5,57	0,001
L43	Lichen ruber planus	2,89	1,84	4,52	< 0,001
L27	Dermatitis durch oral, enteral oder parenteral aufgenommene Substanzen	2,84	1,74	4,64	< 0,001
L81	Sonstige Störungen der Hautpigmentierung	2,83	2,38	3,36	< 0,001
E23	Unterfunktion und andere Störungen der Hypophyse	2,80	1,60	4,91	< 0,001
N72	Entzündliche Krankheit der Cervix uteri	2,75	1,49	5,09	0,001
N90	Sonstige nichtentzündliche Krankheiten der Vulva und des Perineums	2,71	2,08	3,52	< 0,001

L28	Lichen simplex chronicus und Prurigo	2,52	1,78	3,55	< 0,001
K90	Intestinale Malabsorption	2,50	1,79	3,50	< 0,001
A63	Sonstige vorwiegend durch Geschlechtsverkehr übertragene Krankheiten, anderenorts nicht klassifiziert	2,49	1,51	4,09	< 0,001
L50	Urtikaria	2,46	1,98	3,07	< 0,001
E80	Störungen des Porphyrin- und Bilirubinstoffwechsels	2,43	1,58	3,73	< 0,001
D43	Neubildung unsicheren oder unbekannten Verhaltens des Gehirns und des Zentralnervensystems	2,39	1,19	4,78	0,014
M07	Arthritis psoriatica und Arthritiden bei gastrointestinalen Grundkrankheiten	2,32	1,62	3,31	< 0,001
E74	Sonstige Störungen des Kohlenhydratstoffwechsels	2,31	1,71	3,11	< 0,001
F80	Umschriebene Entwicklungsstörungen des Sprechens und der Sprache	2,29	1,39	3,77	0,001
E03	Sonstige Hypothyreose	2,25	2,06	2,46	< 0,001
L01	Impetigo	2,25	1,28	3,96	0,005
E05	Hyperthyreose [Thyreotoxikose]	2,25	1,94	2,61	< 0,001
E07	Sonstige Krankheiten der Schilddrüse	2,25	1,94	2,61	< 0,001
Z01	Sonstige spezielle Untersuchungen und Abklärungen bei Personen ohne Beschwerden oder angegebene Diagnose	2,24	2,04	2,46	< 0,001
B98	Sonstige näher bezeichnete infektiöse Erreger als Ursache von Krankheiten, die in anderen Kapiteln klassifiziert sind	2,23	1,60	3,13	< 0,001
F95	Ticstörungen	2,23	1,40	3,55	< 0,001
N48	Sonstige Krankheiten des Penis	2,22	1,69	2,90	< 0,001
T73	Schäden durch sonstigen Mangel	2,21	1,14	4,28	0,019
L20	Atopisches [endogenes] Ekzem	2,20	1,92	2,53	< 0,001
E53	Mangel an sonstigen Vitaminen des Vitamin-B-Komplexes	2,20	1,87	2,60	< 0,001
N47	Vorhauthypertrophie, Phimose und Paraphimose	2,20	1,53	3,18	< 0,001
D18	Hämangiom und Lymphangiom	2,19	1,85	2,58	< 0,001
I86	Varizen sonstiger Lokalisationen	2,19	1,37	3,49	0,001
L65	Sonstiger Haarausfall ohne Narbenbildung	2,17	1,71	2,74	< 0,001

N46	Sterilität beim Mann	2,16	1,22	3,83	0,009
L84	Hühneraugen und Horn- (Haut-) Schwielen	2,11	1,49	2,99	< 0,001
R61	Hyperhidrose	2,11	1,61	2,76	< 0,001
Q44	Angeborene Fehlbildungen der Gallenblase, der Gallengänge und der Leber	2,09	1,29	3,40	0,003
D17	Gutartige Neubildung des Fettgewebes	2,09	1,69	2,58	< 0,001
J33	Nasenpolyp	2,07	1,56	2,73	< 0,001
B36	Sonstige oberflächliche Mykosen	2,06	1,62	2,61	< 0,001
U81	Gramnegative Erreger mit bestimmten Antibiotikaresistenzen, die besondere therapeutische oder hygienische Maßnahmen erfordern	2,05	1,03	4,05	0,040
D70	Agranulozytose und Neutropenie	2,03	1,40	2,95	< 0,001
B35	Dermatophytose [Tinea]	2,03	1,76	2,34	< 0,001
L08	Sonstige lokale Infektionen der Haut und der Unterhaut	2,02	1,48	2,76	< 0,001
K58	Reizdarmsyndrom	2,01	1,60	2,52	< 0,001
L29	Pruritus	2,01	1,67	2,42	< 0,001
L40	Psoriasis	2,00	1,74	2,31	< 0,001
L91	Hypertrophe Hautkrankheiten	2,00	1,12	3,55	0,019
J47	Bronchiektasen	1,98	1,03	3,81	0,039
M35	Sonstige Krankheiten mit Systembeteiligung des Bindegewebes	1,98	1,64	2,39	< 0,001
D31	Gutartige Neubildung des Auges und der Augenanhangsgebilde	1,96	1,35	2,87	< 0,001
L25	Nicht näher bezeichnete Kontaktdermatitis	1,96	1,25	3,07	0,004
M40	Kyphose und Lordose	1,96	1,62	2,36	< 0,001
G90	Krankheiten des autonomen Nervensystems	1,95	1,15	3,29	0,013
Q43	Sonstige angeborene Fehlbildungen des Darmes	1,94	1,09	3,46	0,024
R23	Sonstige Hautveränderungen	1,93	1,44	2,58	< 0,001
R01	Herzgeräusche und andere Herz-Schallphänomene	1,92	1,17	3,14	0,010
K75	Sonstige entzündliche Leberkrankheiten	1,91	1,25	2,92	0,003
L23	Allergische Kontaktdermatitis	1,91	1,56	2,33	< 0,001
L30	Sonstige Dermatitis	1,90	1,72	2,11	< 0,001

I37	Pulmonalklappenkrankheiten	1,90	1,05	3,44	0,035
T15	Fremdkörper im äußeren Auge	1,89	1,12	3,19	0,017
R79	Sonstige abnorme Befunde der Blutchemie	1,89	1,18	3,03	0,008
L21	Seborrhoisches Ekzem	1,89	1,49	2,38	< 0,001
K14	Krankheiten der Zunge	1,88	1,23	2,86	0,003
I05	Rheumatische Mitralklappenkrankheiten	1,87	1,14	3,07	0,013
N23	Nicht näher bezeichnete Nierenkolik	1,87	1,07	3,26	0,027
Z88	Allergie gegenüber Arzneimitteln, Drogen oder biologisch aktiven Substanzen in der Eigenanamnese	1,86	1,52	2,28	< 0,001
D89	Sonstige Störungen mit Beteiligung des Immunsystems, anderenorts nicht klassifiziert	1,84	1,01	3,35	0,045
S46	Verletzung von Muskeln und Sehnen in Höhe der Schulter und des Oberarmes	1,84	1,16	2,91	0,010
R59	Lymphknotenvergrößerung	1,83	1,33	2,54	< 0,001
H54	Blindheit und Sehbeeinträchtigung	1,83	1,41	2,37	< 0,001
J35	Chronische Krankheiten der Gaumenmandeln und der Rachenmandel	1,83	1,26	2,65	0,002
E73	Laktoseintoleranz	1,82	1,44	2,30	< 0,001
R16	Hepatomegalie und Splenomegalie, anderenorts nicht klassifiziert	1,82	1,35	2,45	< 0,001
C80	Bösartige Neubildung ohne Angabe der Lokalisation	1,81	1,29	2,54	< 0,001
R80	Isolierte Proteinurie	1,81	1,38	2,37	< 0,001
E29	Testikuläre Dysfunktion	1,80	1,15	2,83	0,010
D22	Melanozytennävus	1,80	1,65	1,97	< 0,001
D37	Neubildung unsicheren oder unbekannten Verhaltens der Mundhöhle und der Verdauungsorgane	1,80	1,26	2,57	0,001
N40	Prostatahyperplasie	1,80	1,58	2,05	< 0,001
E65	Lokalisierte Adipositas	1,79	0,94	3,43	0,077
M86	Osteomyelitis	1,78	1,06	3,00	0,029
I85	Ösophagusvarizen	1,78	0,94	3,39	0,079
R33	Harnverhaltung	1,77	1,21	2,60	0,003
F91	Störungen des Sozialverhaltens	1,77	0,97	3,22	0,062
Q63	Sonstige angeborene Fehlbildungen der Niere	1,76	1,13	2,75	0,013

S76	Verletzung von Muskeln und Sehnen in Höhe der Hüfte und des Oberschenkels	1,76	1,05	2,96	0,032
M05	Seropositive chronische Polyarthritis	1,76	1,25	2,48	0,001
E55	Vitamin-D-Mangel	1,76	1,55	1,99	< 0,001
K30	Funktionelle Dyspepsie	1,74	1,21	2,51	0,003
S69	Sonstige und nicht näher bezeichnete Verletzungen des Handgelenkes und der Hand	1,74	0,89	3,42	0,108
M20	Erworbene Deformitäten der Finger und Zehen	1,74	1,51	2,00	< 0,001
M06	Sonstige chronische Polyarthritis	1,74	1,45	2,08	< 0,001
Q14	Angeborene Fehlbildung des hinteren Augenabschnittes	1,74	0,98	3,07	0,058
D40	Neubildung unsicheren oder unbekannten Verhaltens der männlichen Genitalorgane	1,74	1,06	2,84	0,028
K04	Krankheiten der Pulpa und des periapikalen Gewebes	1,73	0,91	3,30	0,094
R82	Sonstige abnorme Urinbefunde	1,73	1,19	2,51	0,004
M41	Skoliose	1,72	1,51	1,97	< 0,001
B00	Infektionen durch Herpesviren [Herpes simplex]	1,72	1,27	2,33	< 0,001
N50	Sonstige Krankheiten der männlichen Genitalorgane	1,72	1,18	2,50	0,004
F40	Phobische Störungen	1,72	1,37	2,16	< 0,001
J39	Sonstige Krankheiten der oberen Atemwege	1,71	1,11	2,65	0,016
D36	Gutartige Neubildung an sonstigen und nicht näher bezeichneten Lokalisationen	1,71	1,29	2,26	< 0,001
J84	Sonstige interstitielle Lungenkrankheiten	1,71	0,97	3,01	0,066
N42	Sonstige Krankheiten der Prostata	1,70	1,18	2,47	0,005
L71	Rosazea	1,70	1,44	2,01	< 0,001
J30	Vasomotorische und allergische Rhinopathie	1,69	1,54	1,86	< 0,001
K13	Sonstige Krankheiten der Lippe und der Mundschleimhaut	1,69	1,06	2,70	0,026
I08	Krankheiten mehrerer Herzklappen	1,69	1,05	2,72	0,031
B07	Viruswarzen	1,69	1,40	2,03	< 0,001
F54	Psychologische Faktoren oder Verhaltensfaktoren bei anderenorts klassifizierten Krankheiten	1,68	1,16	2,44	0,006
R35	Polyurie, Pollakisurie und Nykturie	1,68	1,27	2,21	< 0,001

T78	Unerwünschte Nebenwirkungen, anderenorts nicht klassifiziert	1,68	1,50	1,88	< 0,001
D21	Sonstige gutartige Neubildungen des Bindegewebes und anderer Weichteilgewebe	1,67	1,33	2,11	< 0,001
I83	Varizen der unteren Extremitäten	1,67	1,51	1,85	< 0,001
L64	Alopecia androgenetica	1,67	1,04	2,69	0,035
H36	Affektionen der Netzhaut bei anderenorts klassifizierten Krankheiten	1,66	1,31	2,12	< 0,001
M45	Spondylitis ankylosans	1,66	1,13	2,44	0,009
E61	Mangel an sonstigen Spurenelementen	1,66	1,29	2,15	< 0,001
Z84	Andere Krankheiten oder Zustände in der Familienanamnese	1,66	1,04	2,65	0,032
H18	Sonstige Affektionen der Hornhaut	1,66	1,28	2,16	< 0,001
E34	Sonstige endokrine Störungen	1,66	1,25	2,20	< 0,001
D39	Neubildung unsicheren oder unbekannten Verhaltens der weiblichen Genitalorgane	1,66	1,02	2,70	0,043
N87	Dysplasie der Cervix uteri	1,65	1,16	2,34	0,005
D24	Gutartige Neubildung der Brustdrüse [Mamma]	1,65	1,06	2,55	0,025
J32	Chronische Sinusitis	1,65	1,43	1,90	< 0,001
T88	Sonstige Komplikationen bei chirurgischen Eingriffen und medizinischer Behandlung, anderenorts nicht klassifiziert	1,64	1,44	1,87	< 0,001
O99	Sonstige Krankheiten der Mutter, die anderenorts klassifizierbar sind, die jedoch Schwangerschaft, Geburt und Wochenbett komplizieren	1,64	1,03	2,60	0,035
L60	Krankheiten der Nägel	1,63	1,30	2,05	< 0,001
D52	Folsäure-Mangelanämie	1,63	0,96	2,79	0,072
L82	Seborrhoische Keratose	1,63	1,41	1,89	< 0,001
Q65	Angeborene Deformitäten der Hüfte	1,63	1,15	2,30	0,006
Z31	Fertilisationsfördernde Maßnahmen	1,63	1,20	2,21	0,002
K63	Sonstige Krankheiten des Darmes	1,63	1,38	1,91	< 0,001
N77	Vulvovaginale Ulzeration und Entzündung bei anderenorts klassifizierten Krankheiten	1,63	1,01	2,62	0,045
I78	Krankheiten der Kapillaren	1,62	1,11	2,36	0,012

N88	Sonstige nichtentzündliche Krankheiten der Cervix uteri	1,62	1,10	2,38	0,014
O36	Betreuung der Mutter wegen sonstiger festgestellter oder vermuteter Komplikationen beim Fetus	1,62	0,84	3,11	0,148
F61	Kombinierte und andere Persönlichkeitsstörungen	1,62	0,92	2,86	0,097
S63	Luxation, Verstauchung und Zerrung von Gelenken und Bändern in Höhe des Handgelenkes und der Hand	1,62	1,13	2,31	0,008
M15	Polyarthrose	1,62	1,39	1,88	< 0,001
K57	Divertikelkrankheit des Darmes	1,61	1,43	1,81	< 0,001
E04	Sonstige nichttoxische Struma	1,61	1,46	1,77	< 0,001
D50	Eisenmangelanämie	1,61	1,38	1,88	< 0,001
I38	Endokarditis, Herzklappe nicht näher bezeichnet	1,61	0,89	2,90	0,114
F52	Sexuelle Funktionsstörungen, nicht verursacht durch eine organische Störung oder Krankheit	1,61	1,35	1,91	< 0,001
M85	Sonstige Veränderungen der Knochendichte und -struktur	1,61	1,20	2,15	0,001
S43	Luxation, Verstauchung und Zerrung von Gelenken und Bändern des Schultergürtels	1,60	1,00	2,54	0,048
K62	Sonstige Krankheiten des Anus und des Rektums	1,60	1,24	2,06	< 0,001
H93	Sonstige Krankheiten des Ohres, anderenorts nicht klassifiziert	1,60	1,40	1,82	< 0,001
L57	Hautveränderungen durch chronische Exposition gegenüber nichtionisierender Strahlung	1,59	1,34	1,88	< 0,001
J31	Chronische Rhinitis, Rhinopharyngitis und Pharyngitis	1,59	1,30	1,94	< 0,001
L03	Phlegmone	1,58	1,20	2,09	0,001
D23	Sonstige gutartige Neubildungen der Haut	1,58	1,30	1,91	< 0,001
F81	Umschriebene Entwicklungsstörungen schulischer Fertigkeiten	1,57	0,84	2,95	0,157
N45	Orchitis und Epididymitis	1,57	0,88	2,79	0,124
D29	Gutartige Neubildung der männlichen Genitalorgane	1,57	1,15	2,15	0,005
M81	Osteoporose ohne pathologische Fraktur	1,57	1,35	1,82	< 0,001
Z83	Andere spezifische Krankheiten in der Familienanamnese	1,57	1,04	2,36	0,031

D75	Sonstige Krankheiten des Blutes und der blutbildenden Organe	1,56	1,06	2,31	0,024
K44	Hernia diaphragmatica	1,56	1,33	1,83	< 0,001
H27	Sonstige Affektionen der Linse	1,56	1,09	2,22	0,015
R63	Symptome, die die Nahrungs- und Flüssigkeitsaufnahme betreffen	1,55	1,26	1,90	< 0,001
Z09	Nachuntersuchung nach Behandlung wegen anderer Krankheitszustände außer bösartigen Neubildungen	1,55	1,17	2,05	0,002
F98	Andere Verhaltens- und emotionale Störungen mit Beginn in der Kindheit und Jugend	1,55	0,95	2,53	0,082
Z12	Spezielle Verfahren zur Untersuchung auf Neubildungen	1,55	1,43	1,67	< 0,001
N26	Schrumpfniere, nicht näher bezeichnet	1,54	0,95	2,51	0,081
K80	Cholelithiasis	1,54	1,34	1,79	< 0,001
K31	Sonstige Krankheiten des Magens und des Duodenums	1,54	1,19	1,99	< 0,001
E89	Endokrine und Stoffwechselstörungen nach medizinischen Maßnahmen, anderenorts nicht klassifiziert	1,54	1,26	1,88	< 0,001
C85	Sonstige und nicht näher bezeichnete Typen des Non-Hodgkin-Lymphoms	1,54	0,89	2,65	0,123
B18	Chronische Virushepatitis	1,53	0,90	2,60	0,113
D25	Leiomyom des Uterus	1,53	1,27	1,85	< 0,001
M21	Sonstige erworbene Deformitäten der Extremitäten	1,53	1,36	1,72	< 0,001
K64	Hämorrhoiden und Perianalvenenthrombose	1,53	1,35	1,73	< 0,001
D13	Gutartige Neubildung sonstiger und ungenau bezeichneter Teile des Verdauungssystems	1,53	0,94	2,49	0,085
N80	Endometriose	1,53	1,08	2,17	0,016
D04	Carcinoma in situ der Haut	1,53	1,21	1,94	< 0,001
L85	Sonstige Epidermisverdickung	1,53	1,21	1,94	< 0,001
D69	Purpura und sonstige hämorrhagische Diathesen	1,53	1,16	2,01	0,003
L98	Sonstige Krankheiten der Haut und der Unterhaut, anderenorts nicht klassifiziert	1,53	1,14	2,04	0,004

D90	Immunkompromittierung nach Bestrahlung, Chemotherapie und sonstigen immunsuppressiven Maßnahmen	1,53	0,87	2,69	0,144
Q66	Angeborene Deformitäten der Füße	1,52	1,38	1,69	< 0,001
H04	Affektionen des Tränenapparates	1,52	1,33	1,75	< 0,001
M22	Krankheiten der Patella	1,52	1,24	1,87	< 0,001
N31	Neuromuskuläre Dysfunktion der Harnblase, anderenorts nicht klassifiziert	1,52	1,14	2,02	0,004
R94	Abnorme Ergebnisse von Funktionsprüfungen	1,52	1,23	1,88	< 0,001
Q61	Zystische Nierenkrankheit	1,51	1,21	1,89	< 0,001
D80	Immundefekt mit vorherrschendem Antikörpermangel	1,51	0,89	2,57	0,126
M46	Sonstige entzündliche Spondylopathien	1,51	0,96	2,37	0,074
K29	Gastritis und Duodenitis	1,51	1,36	1,67	< 0,001
T82	Komplikationen durch Prothesen, Implantate oder Transplantate im Herzen und in den Gefäßen	1,51	0,89	2,56	0,129
U08	COVID-19 in der Eigenanamnese	1,50	1,30	1,74	< 0,001
R22	Lokalisierte Schwellung, Raumforderung und Knoten der Haut und der Unterhaut	1,50	0,97	2,34	0,071
Z25	Notwendigkeit der Impfung [Immunisierung] gegen andere einzelne Viruskrankheiten	1,50	1,38	1,63	< 0,001
H02	Sonstige Affektionen des Augenlides	1,50	1,19	1,88	< 0,001
H44	Affektionen des Augapfels	1,50	0,92	2,43	0,103
A46	Erysipel [Wundrose]	1,50	1,07	2,08	0,017
G56	Mononeuropathien der oberen Extremität	1,49	1,27	1,75	< 0,001
D84	Sonstige Immundefekte	1,49	0,76	2,91	0,242
T63	Toxische Wirkung durch Kontakt mit giftigen Tieren	1,49	1,07	2,08	0,019
L53	Sonstige erythematöse Krankheiten	1,49	0,83	2,67	0,182
R68	Sonstige Allgemeinsymptome	1,49	1,04	2,13	0,031
M18	Rhizarthrose [Arthrose des Daumensattelgelenkes]	1,48	1,22	1,80	< 0,001
J34	Sonstige Krankheiten der Nase und der Nasennebenhöhlen	1,48	1,26	1,73	< 0,001
Y57	Unerwünschte Nebenwirkungen bei therapeutischer Anwendung von Arzneimitteln und Drogen	1,48	0,98	2,24	0,066
K82	Sonstige Krankheiten der Gallenblase	1,48	1,01	2,15	0,042

I82	Sonstige venöse Embolie und Thrombose	1,48	1,04	2,10	0,031
N08	Glomeruläre Krankheiten bei anderenorts klassifizierten Krankheiten	1,47	1,19	1,83	< 0,001
R19	Sonstige Symptome, die das Verdauungssystem und das Abdomen betreffen	1,47	1,05	2,05	0,024
M47	Spondylose	1,47	1,34	1,61	< 0,001
N41	Entzündliche Krankheiten der Prostata	1,47	0,97	2,23	0,071
D64	Sonstige Anämien	1,47	1,21	1,77	< 0,001
E21	Hyperparathyreoidismus und sonstige Krankheiten der Nebenschilddrüse	1,46	0,98	2,18	0,061
F42	Zwangsstörung	1,46	0,99	2,17	0,059
H73	Sonstige Krankheiten des Trommelfells	1,46	1,03	2,07	0,032
Z04	Untersuchung und Beobachtung aus sonstigen Gründen	1,46	1,00	2,13	0,051
F45	Somatoforme Störungen	1,46	1,33	1,60	< 0,001
I73	Sonstige periphere Gefäßkrankheiten	1,46	1,17	1,82	< 0,001
N25	Krankheiten infolge Schädigung der tubulären Nierenfunktion	1,46	0,83	2,56	0,192
N60	Gutartige Mammadysplasie [Brustdrüsendysplasie]	1,46	1,14	1,87	0,003
Z13	Spezielle Verfahren zur Untersuchung auf sonstige Krankheiten oder Störungen	1,45	1,12	1,90	0,006
R41	Sonstige Symptome, die das Erkennungsvermögen und das Bewusstsein betreffen	1,45	1,10	1,91	0,008
D12	Gutartige Neubildung des Kolons, des Rektums, des Analkanals und des Anus	1,45	1,18	1,79	< 0,001
R20	Sensibilitätsstörungen der Haut	1,45	1,19	1,77	< 0,001
S80	Oberflächliche Verletzung des Unterschenkels	1,45	1,08	1,94	0,012
H90	Hörverlust durch Schallleitungs- oder Schallempfindungsstörung	1,44	1,27	1,65	< 0,001
M19	Sonstige Arthrose	1,44	1,29	1,61	< 0,001
R77	Sonstige Veränderungen der Plasmaproteine	1,44	0,99	2,09	0,054
H91	Sonstiger Hörverlust	1,44	1,25	1,66	< 0,001
S92	Fraktur des Fußes [ausgenommen oberes Sprunggelenk]	1,44	0,99	2,10	0,055

I67	Sonstige zerebrovaskuläre Krankheiten	1,44	1,21	1,71	< 0,001
Z82	Bestimmte Behinderungen oder chronische Krankheiten in der Familienanamnese, die zu Schädigung oder Behinderung führen	1,44	1,05	1,98	0,025
E14	Nicht näher bezeichneter Diabetes mellitus	1,43	1,26	1,63	< 0,001
E01	Jodmangelbedingte Schilddrüsenkrankheiten und verwandte Zustände	1,43	1,14	1,81	0,002
T08	Fraktur der Wirbelsäule, Höhe nicht näher bezeichnet	1,43	0,93	2,20	0,104
I24	Sonstige akute ischämische Herzkrankheit	1,43	0,74	2,78	0,292
N95	Klimakterische Störungen	1,43	1,28	1,60	< 0,001
Z97	Vorhandensein anderer medizinischer Geräte oder Hilfsmittel	1,43	1,14	1,80	0,002
M79	Sonstige Krankheiten des Weichteilgewebes, anderenorts nicht klassifiziert	1,43	1,29	1,58	< 0,001
E56	Sonstige Vitaminmangelzustände	1,43	0,90	2,27	0,130
O80	Spontangeburt eines Einlings	1,42	0,74	2,72	0,287
Q21	Angeborene Fehlbildungen der Herzsepten	1,42	0,91	2,22	0,127
M75	Schulterläsionen	1,42	1,27	1,59	< 0,001
Z72	Probleme mit Bezug auf die Lebensführung	1,42	0,94	2,14	0,098
Z24	Notwendigkeit der Impfung [Immunisierung] gegen bestimmte einzelne Viruskrankheiten	1,42	1,23	1,63	< 0,001
N84	Polyp des weiblichen Genitaltraktes	1,41	0,93	2,16	0,109
R45	Symptome, die die Stimmung betreffen	1,41	1,14	1,75	0,001
B97	Viren als Ursache von Krankheiten, die in anderen Kapiteln klassifiziert sind	1,41	0,88	2,26	0,151
G43	Migräne	1,41	1,24	1,60	< 0,001
K10	Sonstige Krankheiten der Kiefer	1,41	0,73	2,74	0,312
H16	Keratitis	1,41	0,99	2,01	0,060
Z91	Risikofaktoren in der Eigenanamnese, anderenorts nicht klassifiziert	1,41	1,05	1,88	0,022
Z96	Vorhandensein von anderen funktionellen Implantaten	1,41	1,26	1,57	< 0,001
K76	Sonstige Krankheiten der Leber	1,40	1,26	1,56	< 0,001
L70	Akne	1,40	1,13	1,74	0,002
I13	Hypertensive Herz- und Nierenkrankheit	1,40	0,86	2,28	0,175

Z00	Allgemeinuntersuchung und Abklärung bei Personen ohne Beschwerden oder angegebene Diagnose	1,40	1,29	1,52	< 0,001
I79	Krankheiten der Arterien, Arteriolen und Kapillaren bei anderenorts klassifizierten Krankheiten	1,40	0,99	1,98	0,058
K85	Akute Pankreatitis	1,40	0,83	2,37	0,212
G24	Dystonie	1,40	0,78	2,50	0,260
S13	Luxation, Verstauchung und Zerrung von Gelenken und Bändern in Halshöhe	1,39	0,90	2,16	0,140
H52	Akkommodationsstörungen und Refraktionsfehler	1,39	1,29	1,51	< 0,001
H20	Iridozyklitis	1,39	0,77	2,50	0,269
R27	Sonstige Koordinationsstörungen	1,39	0,85	2,28	0,193
Z80	Bösartige Neubildung in der Familienanamnese	1,39	1,18	1,64	< 0,001
M96	Krankheiten des Muskel-Skelett-Systems nach medizinischen Maßnahmen, anderenorts nicht klassifiziert	1,39	0,89	2,17	0,151
H10	Konjunktivitis	1,39	1,21	1,60	< 0,001
G63	Polyneuropathie bei anderenorts klassifizierten Krankheiten	1,39	1,17	1,64	< 0,001
M87	Knochennekrose	1,39	0,73	2,61	0,314
G93	Sonstige Krankheiten des Gehirns	1,39	1,06	1,80	0,016
C73	Bösartige Neubildung der Schilddrüse	1,38	0,71	2,69	0,338
J11	Grippe, Viren nicht nachgewiesen	1,38	0,96	1,99	0,080
L73	Sonstige Krankheiten der Haarfollikel	1,38	1,01	1,88	0,040
J45	Asthma bronchiale	1,38	1,24	1,53	< 0,001
H92	Otalgie und Ohrenfluss	1,38	1,07	1,77	0,012
R00	Störungen des Herzschlages	1,38	1,18	1,62	< 0,001
K60	Fissur und Fistel in der Anal- und Rektalregion	1,38	0,94	2,03	0,102
F34	Anhaltende affektive Störungen	1,38	1,10	1,73	0,006
M99	Biomechanische Funktionsstörungen, anderenorts nicht klassifiziert	1,38	1,25	1,52	< 0,001
M77	Sonstige Enthesopathien	1,38	1,23	1,54	< 0,001
G57	Mononeuropathien der unteren Extremität	1,37	1,03	1,83	0,031
G25	Sonstige extrapyramidale Krankheiten und Bewegungsstörungen	1,37	1,12	1,68	0,002

M71	Sonstige Bursopathien	1,37	1,05	1,79	0,021
I95	Hypotonie	1,37	1,13	1,66	0,001
E78	Störungen des Lipoproteinstoffwechsels und sonstige Lipidämien	1,37	1,27	1,48	< 0,001
K83	Sonstige Krankheiten der Gallenwege	1,37	0,75	2,51	0,309
Z53	Personen, die Einrichtungen des Gesundheitswesens wegen spezifischer Maßnahmen aufgesucht haben, die aber nicht durchgeführt wurden	1,37	0,70	2,66	0,354
N30	Zystitis	1,37	1,14	1,63	< 0,001
N81	Genitalprolaps bei der Frau	1,37	1,12	1,66	0,002
Z08	Nachuntersuchung nach Behandlung wegen bösartiger Neubildung	1,37	1,09	1,71	0,007
J00	Akute Rhinopharyngitis [Erkältungsschnupfen]	1,36	1,16	1,61	< 0,001
K59	Sonstige funktionelle Darmstörungen	1,36	1,15	1,62	< 0,001
R73	Erhöhter Blutglukosewert	1,36	1,11	1,68	0,004
H01	Sonstige Entzündung des Augenlides	1,36	1,05	1,76	0,020
K40	Hernia inguinalis	1,36	1,06	1,73	0,014
M24	Sonstige näher bezeichnete Gelenkschädigungen	1,36	1,04	1,77	0,025
M10	Gicht	1,36	1,14	1,61	< 0,001
Z35	Überwachung einer Risikoschwangerschaft	1,35	0,85	2,16	0,205
M17	Gonarthrose [Arthrose des Kniegelenkes]	1,35	1,22	1,49	< 0,001
F48	Andere neurotische Störungen	1,35	1,16	1,57	< 0,001
D34	Gutartige Neubildung der Schilddrüse	1,35	0,82	2,22	0,233
Z26	Notwendigkeit der Impfung [Immunisierung] gegen andere einzelne Infektionskrankheiten	1,35	1,18	1,55	< 0,001
K22	Sonstige Krankheiten des Ösophagus	1,35	1,02	1,78	0,034
Q82	Sonstige angeborene Fehlbildungen der Haut	1,35	0,89	2,03	0,158
O26	Betreuung der Mutter bei sonstigen Zuständen, die vorwiegend mit der Schwangerschaft verbunden sind	1,35	0,83	2,19	0,231
R69	Unbekannte und nicht näher bezeichnete Krankheitsursachen	1,34	0,84	2,15	0,217
M65	Synovitis und Tenosynovitis	1,34	1,13	1,60	0,001
S86	Verletzung von Muskeln und Sehnen in Höhe des Unterschenkels	1,34	0,77	2,35	0,303

M95	Sonstige erworbene Deformitäten des Muskel-Skelett-Systems und des Bindegewebes	1,34	0,95	1,89	0,091
I87	Sonstige Venenkrankheiten	1,34	1,16	1,55	< 0,001
H26	Sonstige Kataraktformen	1,34	1,18	1,52	< 0,001
Z49	Dialysebehandlung	1,34	0,71	2,53	0,365
M72	Fibromatosen	1,34	1,10	1,64	0,004
R12	Sodbrennen	1,33	1,01	1,76	0,043
G58	Sonstige Mononeuropathien	1,33	0,98	1,82	0,071
K42	Hernia umbilicalis	1,33	0,96	1,84	0,082
N18	Chronische Nierenkrankheit	1,33	1,16	1,52	< 0,001
J04	Akute Laryngitis und Tracheitis	1,33	1,04	1,71	0,025
Z32	Untersuchung und Test zur Feststellung einer Schwangerschaft	1,33	0,79	2,23	0,282
M42	Osteochondrose der Wirbelsäule	1,33	1,18	1,49	< 0,001
G62	Sonstige Polyneuropathien	1,33	1,13	1,56	< 0,001
C43	Bösartiges Melanom der Haut	1,33	0,92	1,90	0,126
H43	Affektionen des Glaskörpers	1,32	1,11	1,57	0,001
I49	Sonstige kardiale Arrhythmien	1,32	1,15	1,52	< 0,001
K86	Sonstige Krankheiten des Pankreas	1,32	0,99	1,76	0,060
Z71	Personen, die das Gesundheitswesen zum Zwecke anderer Beratung oder ärztlicher Konsultation in Anspruch nehmen, anderenorts nicht klassifiziert	1,32	1,17	1,49	< 0,001
R07	Hals- und Brustschmerzen	1,32	1,14	1,53	< 0,001
J20	Akute Bronchitis	1,32	1,13	1,55	< 0,001
I45	Sonstige kardiale Erregungsleitungsstörungen	1,32	1,02	1,70	0,036
C18	Bösartige Neubildung des Kolons	1,32	0,91	1,91	0,149
N86	Erosion und Ektropium der Cervix uteri	1,31	1,02	1,70	0,037
H57	Sonstige Affektionen des Auges und der Augenanhangsgebilde	1,31	0,97	1,79	0,081
S05	Verletzung des Auges und der Orbita	1,31	0,73	2,35	0,361
M50	Zervikale Bandscheibenschäden	1,31	1,10	1,55	0,002
Z63	Andere Kontaktanlässe mit Bezug auf den engeren Familienkreis	1,31	0,89	1,93	0,177

C20	Bösartige Neubildung des Rektums	1,31	0,72	2,39	0,382
B37	Kandidose	1,31	1,03	1,66	0,026
K51	Colitis ulcerosa	1,31	0,91	1,87	0,144
K91	Krankheiten des Verdauungssystems nach medizinischen Maßnahmen, anderenorts nicht klassifiziert	1,31	0,83	2,07	0,252
J02	Akute Pharyngitis	1,31	1,08	1,57	0,005
M23	Binnenschädigung des Kniegelenkes [internal derangement]	1,31	1,14	1,50	< 0,001
N76	Sonstige entzündliche Krankheit der Vagina und Vulva	1,31	1,10	1,55	0,003
N28	Sonstige Krankheiten der Niere und des Ureters, anderenorts nicht klassifiziert	1,30	1,11	1,53	0,001
L72	Follikuläre Zysten der Haut und der Unterhaut	1,30	1,01	1,68	0,038
Z30	Kontrazeptive Maßnahmen	1,30	1,14	1,49	< 0,001
R10	Bauch- und Beckenschmerzen	1,30	1,18	1,44	< 0,001
M51	Sonstige Bandscheibenschäden	1,30	1,18	1,44	< 0,001
G55	Kompression von Nervenwurzeln und Nervenplexus bei anderenorts klassifizierten Krankheiten	1,30	1,10	1,54	0,002
E28	Ovarielle Dysfunktion	1,30	0,97	1,75	0,084
D72	Sonstige Krankheiten der Leukozyten	1,30	0,87	1,95	0,207
M25	Sonstige Gelenkkrankheiten, anderenorts nicht klassifiziert	1,30	1,18	1,43	< 0,001
N92	Zu starke, zu häufige oder unregelmäßige Menstruation	1,30	1,09	1,55	0,004
B95	Streptokokken und Staphylokokken als Ursache von Krankheiten, die in anderen Kapiteln klassifiziert sind	1,30	0,96	1,75	0,089
Z73	Probleme mit Bezug auf Schwierigkeiten bei der Lebensbewältigung	1,29	1,04	1,62	0,024
H81	Störungen der Vestibularfunktion	1,29	1,05	1,59	0,014
A69	Sonstige Spirochäteninfektionen	1,29	0,90	1,86	0,171
S40	Oberflächliche Verletzung der Schulter und des Oberarmes	1,29	0,85	1,94	0,229
M62	Sonstige Muskelkrankheiten	1,29	1,14	1,45	< 0,001
M48	Sonstige Spondylopathien	1,29	1,11	1,49	< 0,001

H61	Sonstige Krankheiten des äußeren Ohres	1,29	1,16	1,43	< 0,001
M54	Rückenschmerzen	1,29	1,20	1,38	< 0,001
E79	Störungen des Purin- und Pyrimidinstoffwechsels	1,29	1,13	1,46	< 0,001
I77	Sonstige Krankheiten der Arterien und Arteriolen	1,28	0,80	2,05	0,294
F41	Andere Angststörungen	1,28	1,14	1,44	< 0,001
F32	Depressive Episode	1,28	1,17	1,40	< 0,001
G50	Krankheiten des N. trigeminus [V. Hirnnerv]	1,28	0,81	2,02	0,284
F60	Spezifische Persönlichkeitsstörungen	1,28	0,97	1,70	0,084
I34	Nichtrheumatische Mitralklappenkrankheiten	1,28	1,10	1,50	0,002
Z47	Andere orthopädische Nachbehandlung	1,28	0,78	2,10	0,325
H00	Hordeolum und Chalazion	1,28	0,97	1,70	0,085
K81	Cholezystitis	1,28	0,72	2,28	0,405
Z23	Notwendigkeit der Impfung [Immunisierung] gegen einzelne bakterielle Krankheiten	1,28	1,05	1,55	0,013
R53	Unwohlsein und Ermüdung	1,28	1,12	1,46	< 0,001
R30	Schmerzen beim Wasserlassen	1,27	0,95	1,71	0,103
N89	Sonstige nichtentzündliche Krankheiten der Vagina	1,27	1,15	1,41	< 0,001
I51	Komplikationen einer Herzkrankheit und ungenau beschriebene Herzkrankheit	1,27	1,04	1,56	0,022
Z27	Notwendigkeit der Impfung [Immunisierung] gegen Kombinationen von Infektionskrankheiten	1,27	1,11	1,45	< 0,001
M89	Sonstige Knochenkrankheiten	1,27	0,85	1,90	0,251
U07	Krankheiten mit unklarer Ätiologie, belegte und nicht belegte Schlüsselnummern U07.-	1,27	1,13	1,41	< 0,001
R04	Blutung aus den Atemwegen	1,27	0,92	1,74	0,149
Z03	Ärztliche Beobachtung und Beurteilung von Verdachtsfällen, Verdacht ausgeschlossen	1,26	1,01	1,59	0,045
H59	Affektionen des Auges und der Augenanhangsgebilde nach medizinischen Maßnahmen, anderenorts nicht klassifiziert	1,26	0,83	1,92	0,273
O09	Schwangerschaftsdauer	1,26	0,89	1,80	0,193
Z51	Sonstige medizinische Behandlung	1,26	1,07	1,49	0,005
M43	Sonstige Deformitäten der Wirbelsäule und des Rückens	1,26	1,05	1,51	0,012

M13	Sonstige Arthritis	1,26	1,00	1,60	0,053
I15	Sekundäre Hypertonie	1,26	0,82	1,93	0,286
R31	Nicht näher bezeichnete Hämaturie	1,26	1,04	1,54	0,020
G45	Zerebrale transitorische Ischämie und verwandte Syndrome	1,26	0,91	1,75	0,168
K56	Paralytischer Ileus und intestinale Obstruktion ohne Hernie	1,26	0,85	1,86	0,243
D48	Neubildung unsicheren oder unbekannten Verhaltens an sonstigen und nicht näher bezeichneten Lokalisationen	1,26	1,01	1,57	0,037
U11	Notwendigkeit der Impfung gegen COVID-19	1,26	1,12	1,42	< 0,001
K66	Sonstige Krankheiten des Peritoneums	1,26	0,86	1,84	0,240
M94	Sonstige Knorpelkrankheiten	1,26	0,97	1,63	0,082
R74	Abnorme Serumenzymwerte	1,26	0,96	1,65	0,099
M16	Koxarthrose [Arthrose des Hüftgelenkes]	1,26	1,10	1,43	< 0,001
G99	Sonstige Krankheiten des Nervensystems bei anderenorts klassifizierten Krankheiten	1,26	0,70	2,25	0,442
N93	Sonstige abnorme Uterus- oder Vaginalblutung	1,26	0,88	1,80	0,212
N32	Sonstige Krankheiten der Harnblase	1,26	0,94	1,68	0,123
H53	Sehstörungen	1,26	1,10	1,43	< 0,001
C91	Lymphatische Leukämie	1,25	0,67	2,35	0,481
R43	Störungen des Geruchs- und Geschmackssinnes	1,25	0,77	2,05	0,371
M70	Krankheiten des Weichteilgewebes im Zusammenhang mit Beanspruchung, Überbeanspruchung und Druck	1,25	0,98	1,60	0,075
R25	Abnorme unwillkürliche Bewegungen	1,25	0,96	1,63	0,103
M76	Enthesopathien der unteren Extremität mit Ausnahme des Fußes	1,25	1,01	1,55	0,042
M80	Osteoporose mit pathologischer Fraktur	1,25	0,92	1,70	0,162
H19	Affektionen der Sklera und der Hornhaut bei anderenorts klassifizierten Krankheiten	1,25	0,81	1,91	0,314
F50	Essstörungen	1,25	0,83	1,88	0,295
J06	Akute Infektionen an mehreren oder nicht näher bezeichneten Lokalisationen der oberen Atemwege	1,24	1,15	1,35	< 0,001
N43	Hydrozele und Spermatozele	1,24	0,85	1,81	0,255

N39	Sonstige Krankheiten des Harnsystems	1,24	1,11	1,39	< 0,001
Z48	Andere Nachbehandlung nach chirurgischem Eingriff	1,24	1,02	1,52	0,033
G44	Sonstige Kopfschmerzsyndrome	1,24	1,00	1,55	0,055
I47	Paroxysmale Tachykardie	1,24	0,95	1,61	0,108
Z90	Verlust von Organen, anderenorts nicht klassifiziert	1,24	1,07	1,44	0,004
K43	Hernia ventralis	1,24	0,82	1,88	0,314
Z98	Sonstige Zustände nach chirurgischem Eingriff	1,24	1,09	1,41	0,001
M67	Sonstige Krankheiten der Synovialis und der Sehnen	1,24	0,95	1,62	0,116
N62	Hypertrophie der Mamma [Brustdrüse]	1,24	0,82	1,86	0,310
Z37	Resultat der Entbindung	1,24	0,80	1,92	0,345
H25	Cataracta senilis	1,24	1,08	1,41	0,002
N64	Sonstige Krankheiten der Mamma [Brustdrüse]	1,23	1,02	1,49	0,030
I89	Sonstige nichtinfektiöse Krankheiten der Lymphgefäße und Lymphknoten	1,23	1,03	1,47	0,020
A49	Bakterielle Infektion nicht näher bezeichneter Lokalisation	1,23	0,86	1,75	0,252
R05	Husten	1,23	1,07	1,42	0,004
J98	Sonstige Krankheiten der Atemwege	1,23	1,03	1,46	0,022
U99	Belegte und nicht belegte Schlüsselnummern U99.-!	1,23	1,08	1,39	0,001
B02	Zoster [Herpes zoster]	1,22	0,92	1,63	0,164
H65	Nichteitrige Otitis media	1,22	0,87	1,73	0,248
G54	Krankheiten von Nervenwurzeln und Nervenplexus	1,22	0,86	1,75	0,268
F51	Nichtorganische Schlafstörungen	1,22	0,97	1,53	0,090
D32	Gutartige Neubildung der Meningen	1,22	0,73	2,02	0,445
Z76	Personen, die das Gesundheitswesen aus sonstigen Gründen in Anspruch nehmen	1,22	0,99	1,49	0,057
R51	Kopfschmerz	1,22	1,06	1,40	0,005
I35	Nichtrheumatische Aortenklappenkrankheiten	1,22	1,01	1,47	0,040
Z11	Spezielle Verfahren zur Untersuchung auf infektiöse und parasitäre Krankheiten	1,21	1,07	1,38	0,003
R06	Störungen der Atmung	1,21	1,06	1,39	0,006
N85	Sonstige nichtentzündliche Krankheiten des Uterus, ausgenommen der Zervix	1,21	0,90	1,63	0,209

G47	Schlafstörungen	1,21	1,09	1,35	< 0,001
I70	Atherosklerose	1,21	1,05	1,39	0,008
K21	Gastroösophageale Refluxkrankheit	1,21	1,09	1,34	< 0,001
Z33	Schwangerschaftsfeststellung als Nebenbefund	1,21	0,64	2,29	0,564
T14	Verletzung an einer nicht näher bezeichneten Körperregion	1,20	1,06	1,37	0,004
Z34	Überwachung einer normalen Schwangerschaft	1,20	0,81	1,77	0,355
H69	Sonstige Krankheiten der Tuba auditiva	1,20	0,87	1,66	0,260
I80	Thrombose, Phlebitis und Thrombophlebitis	1,20	0,92	1,56	0,169
F43	Reaktionen auf schwere Belastungen und Anpassungsstörungen	1,20	1,08	1,33	< 0,001
H35	Sonstige Affektionen der Netzhaut	1,20	1,04	1,37	0,010
M93	Sonstige Osteochondropathien	1,20	0,93	1,54	0,169
K07	Dentofaziale Anomalien [einschließlich fehlerhafter Okklusion]	1,19	0,81	1,76	0,370
Z60	Kontaktanlässe mit Bezug auf die soziale Umgebung	1,19	0,76	1,88	0,450
C50	Bösartige Neubildung der Brustdrüse [Mamma]	1,19	0,93	1,52	0,161
I31	Sonstige Krankheiten des Perikards	1,19	0,62	2,30	0,606
F33	Rezidivierende depressive Störung	1,19	1,04	1,35	0,009
F62	Andauernde Persönlichkeitsänderungen, nicht Folge einer Schädigung oder Krankheit des Gehirns	1,18	0,63	2,22	0,597
Z39	Postpartale Betreuung und Untersuchung der Mutter	1,18	0,71	1,97	0,512
R11	Übelkeit und Erbrechen	1,18	0,99	1,42	0,065
H60	Otitis externa	1,18	0,99	1,41	0,058
R42	Schwindel und Taumel	1,18	1,04	1,34	0,009
C44	Sonstige bösartige Neubildungen der Haut	1,18	0,97	1,43	0,091
D47	Sonstige Neubildungen unsicheren oder unbekannten Verhaltens des lymphatischen, blutbildenden und verwandten Gewebes	1,18	0,76	1,82	0,457
H50	Sonstiger Strabismus	1,18	0,96	1,44	0,111
I99	Sonstige und nicht näher bezeichnete Krankheiten des Kreislaufsystems	1,18	0,89	1,56	0,256
Z46	Versorgen mit und Anpassen von anderen medizinischen Geräten oder Hilfsmitteln	1,18	0,99	1,39	0,060

N20	Nieren- und Ureterstein	1,17	0,95	1,45	0,137
S02	Fraktur des Schädels und der Gesichtsschädelknochen	1,17	0,61	2,26	0,635
Z85	Bösartige Neubildung in der Eigenanamnese	1,17	0,89	1,53	0,261
J01	Akute Sinusitis	1,17	0,94	1,45	0,167
N35	Harnröhrenstriktur	1,17	0,69	1,96	0,560
K70	Alkoholische Leberkrankheit	1,17	0,76	1,78	0,478
I36	Nichtrheumatische Trikuspidalklappenkrankheiten	1,16	0,79	1,71	0,435
Z92	Medizinische Behandlung in der Eigenanamnese	1,16	1,03	1,31	0,016
M53	Sonstige Krankheiten der Wirbelsäule und des Rückens, anderenorts nicht klassifiziert	1,16	1,04	1,30	0,009
Z87	Andere Krankheiten oder Zustände in der Eigenanamnese	1,16	0,77	1,76	0,481
I71	Aortenaneurysma und -dissektion	1,16	0,88	1,52	0,281
S50	Oberflächliche Verletzung des Unterarmes	1,16	0,70	1,92	0,565
C61	Bösartige Neubildung der Prostata	1,16	0,88	1,52	0,296
H11	Sonstige Affektionen der Konjunktiva	1,16	0,88	1,53	0,303
Z38	Lebendgeborene nach dem Geburtsort	1,15	0,71	1,88	0,563
G53	Krankheiten der Hirnnerven bei anderenorts klassifizierten Krankheiten	1,15	0,63	2,10	0,642
N91	Ausgebliebene, zu schwache oder zu seltene Menstruation	1,15	0,87	1,53	0,323
R92	Abnorme Befunde bei der bildgebenden Diagnostik der Mamma [Brustdrüse]	1,15	0,61	2,15	0,662
U09	Post-COVID-19-Zustand	1,15	0,84	1,58	0,391
R65	Systemisches inflammatorisches Response-Syndrom [SIRS]	1,15	0,68	1,93	0,603
F99	Psychische Störung ohne nähere Angabe	1,15	0,67	1,97	0,616
J03	Akute Tonsillitis	1,15	0,93	1,41	0,189
H40	Glaukom	1,15	0,99	1,33	0,066
K92	Sonstige Krankheiten des Verdauungssystems	1,15	0,85	1,55	0,375
I65	Verschluss und Stenose präzerebraler Arterien ohne resultierenden Hirninfarkt	1,15	0,96	1,36	0,126
B49	Nicht näher bezeichnete Mykose	1,14	0,73	1,79	0,553

E11	Diabetes mellitus, Typ 2	1,14	1,03	1,26	0,010
K12	Stomatitis und verwandte Krankheiten	1,14	0,63	2,08	0,665
R50	Fieber sonstiger und unbekannter Ursache	1,14	0,86	1,51	0,355
E66	Adipositas	1,14	1,04	1,25	0,005
I44	Atrioventrikulärer Block und Linksschenkelblock	1,14	0,91	1,42	0,247
E83	Störungen des Mineralstoffwechsels	1,14	0,81	1,60	0,452
H34	Netzhautgefäßverschluss	1,14	0,69	1,88	0,621
N83	Nichtentzündliche Krankheiten des Ovars, der Tuba uterina und des Lig. latum uteri	1,14	0,86	1,49	0,362
E88	Sonstige Stoffwechselstörungen	1,13	0,93	1,38	0,210
S42	Fraktur im Bereich der Schulter und des Oberarmes	1,13	0,76	1,69	0,543
Q74	Sonstige angeborene Fehlbildungen der Extremität(en)	1,13	0,66	1,93	0,655
R52	Schmerz, anderenorts nicht klassifiziert	1,13	1,01	1,26	0,029
R91	Abnorme Befunde bei der bildgebenden Diagnostik der Lunge	1,13	0,71	1,80	0,612
B34	Viruskrankheit nicht näher bezeichneter Lokalisation	1,13	0,95	1,33	0,157
R13	Dysphagie	1,12	0,85	1,48	0,401
E71	Störungen des Stoffwechsels verzweigter Aminosäuren und des Fettsäurestoffwechsels	1,12	0,60	2,10	0,716
H47	Sonstige Affektionen des N. opticus [II. Hirnnerv] und der Sehbahn	1,12	0,91	1,38	0,269
F06	Andere psychische Störungen aufgrund einer Schädigung oder Funktionsstörung des Gehirns oder einer körperlichen Krankheit	1,12	0,84	1,49	0,432
S82	Fraktur des Unterschenkels, einschließlich des oberen Sprunggelenkes	1,12	0,77	1,63	0,550
C77	Sekundäre und nicht näher bezeichnete bösartige Neubildung der Lymphknoten	1,12	0,72	1,74	0,618
S90	Oberflächliche Verletzung der Knöchelregion und des Fußes	1,12	0,74	1,68	0,596
R55	Synkope und Kollaps	1,11	0,82	1,50	0,500
G31	Sonstige degenerative Krankheiten des Nervensystems, anderenorts nicht klassifiziert	1,11	0,66	1,87	0,696

Z45	Anpassung und Handhabung eines implantierten medizinischen Gerätes	1,11	0,76	1,63	0,596
T79	Bestimmte Frühkomplikationen eines Traumas, anderenorts nicht klassifiziert	1,11	0,83	1,48	0,501
B99	Sonstige und nicht näher bezeichnete Infektionskrankheiten	1,10	0,89	1,36	0,364
I42	Kardiomyopathie	1,10	0,80	1,51	0,546
E46	Nicht näher bezeichnete Energie- und Eiweißmangelernährung	1,10	0,59	2,06	0,763
S20	Oberflächliche Verletzung des Thorax	1,10	0,80	1,51	0,556
Z56	Kontaktanlässe mit Bezug auf das Berufsleben	1,10	0,73	1,65	0,653
L02	Hautabszess, Furunkel und Karbunkel	1,09	0,82	1,47	0,549
R89	Abnorme Befunde in Untersuchungsmaterialien aus anderen Körperorganen, -systemen und -geweben	1,09	0,60	1,99	0,769
Z43	Versorgung künstlicher Körperöffnungen	1,09	0,65	1,84	0,738
F90	Hyperkinetische Störungen	1,09	0,73	1,63	0,666
I07	Rheumatische Trikuspidalklappenkrankheiten	1,09	0,84	1,42	0,518
S00	Oberflächliche Verletzung des Kopfes	1,09	0,79	1,51	0,613
S62	Fraktur im Bereich des Handgelenkes und der Hand	1,09	0,68	1,73	0,729
N63	Nicht näher bezeichnete Knoten in der Mamma [Brustdrüse]	1,08	0,56	2,08	0,812
R29	Sonstige Symptome, die das Nervensystem und das Muskel-Skelett-System betreffen	1,08	0,91	1,29	0,380
R49	Störungen der Stimme	1,08	0,79	1,47	0,621
R21	Hautausschlag und sonstige unspezifische Hauteruptionen	1,08	0,74	1,59	0,692
S81	Offene Wunde des Unterschenkels	1,08	0,59	1,97	0,800
D68	Sonstige Koagulopathien	1,08	0,84	1,38	0,544
R14	Flatulenz und verwandte Zustände	1,08	0,81	1,44	0,601
I11	Hypertensive Herzkrankheit	1,08	0,92	1,26	0,348
R39	Sonstige Symptome, die das Harnsystem betreffen	1,08	0,85	1,36	0,543
Z29	Notwendigkeit von anderen prophylaktischen Maßnahmen	1,07	0,81	1,41	0,623
I10	Essentielle (primäre) Hypertonie	1,07	0,99	1,16	0,100

S06	Intrakranielle Verletzung	1,07	0,69	1,65	0,765
D41	Neubildung unsicheren oder unbekannten Verhaltens der Harnorgane	1,07	0,62	1,82	0,815
J42	Nicht näher bezeichnete chronische Bronchitis	1,06	0,77	1,47	0,707
R26	Störungen des Ganges und der Mobilität	1,06	0,91	1,24	0,449
R87	Abnorme Befunde in Untersuchungsmaterialien aus den weiblichen Genitalorganen	1,06	0,71	1,59	0,772
T84	Komplikationen durch orthopädische Endoprothesen, Implantate oder Transplantate	1,06	0,68	1,65	0,797
R60	Ödem, anderenorts nicht klassifiziert	1,05	0,86	1,28	0,614
Z86	Bestimmte andere Krankheiten in der Eigenanamnese	1,04	0,82	1,33	0,726
Z95	Vorhandensein von kardialen oder vaskulären Implantaten oder Transplantaten	1,04	0,89	1,22	0,593
R54	Senilität	1,04	0,85	1,28	0,696
U51	Kognitive Funktionseinschränkung	1,04	0,79	1,38	0,776
S22	Fraktur der Rippe(n), des Sternums und der Brustwirbelsäule	1,04	0,71	1,52	0,851
H33	Netzhautablösung und Netzhautriss	1,04	0,68	1,57	0,870
Z20	Kontakt mit und Exposition gegenüber übertragbaren Krankheiten	1,03	0,67	1,59	0,883
I21	Akuter Myokardinfarkt	1,03	0,74	1,44	0,851
I50	Herzinsuffizienz	1,03	0,89	1,19	0,710
S83	Luxation, Verstauchung und Zerrung des Kniegelenkes und von Bändern des Kniegelenkes	1,02	0,79	1,33	0,854
H31	Sonstige Affektionen der Aderhaut	1,02	0,60	1,74	0,934
K52	Sonstige nichtinfektiöse Gastroenteritis und Kolitis	1,02	0,82	1,26	0,849
I25	Chronische ischämische Herzkrankheit	1,02	0,90	1,16	0,750
N19	Nicht näher bezeichnete Niereninsuffizienz	1,02	0,80	1,31	0,874
C64	Bösartige Neubildung der Niere, ausgenommen Nierenbecken	1,02	0,55	1,90	0,956
R18	Aszites	1,02	0,55	1,89	0,962
S30	Oberflächliche Verletzung des Abdomens, der Lumbosakralgegend und des Beckens	1,01	0,65	1,58	0,955
S52	Fraktur des Unterarmes	1,01	0,68	1,51	0,952

I48	Vorhofflimmern und Vorhofflattern	1,01	0,86	1,18	0,936
H66	Eitrige und nicht näher bezeichnete Otitis media	1,00	0,74	1,35	0,986
E13	Sonstiger näher bezeichneter Diabetes mellitus	1,00	0,61	1,65	0,999
F13	Psychische und Verhaltensstörungen durch Sedativa oder Hypnotika	1,00	0,54	1,86	0,999
H68	Entzündung und Verschluss der Tuba auditiva	1,00	0,65	1,54	0,999
U50	Motorische Funktionseinschränkung	1,00	0,83	1,20	0,987
S60	Oberflächliche Verletzung des Handgelenkes und der Hand	1,00	0,70	1,41	0,979
I26	Lungenembolie	0,99	0,64	1,53	0,975
E87	Sonstige Störungen des Wasser- und Elektrolythaushaltes sowie des Säure-Basen-Gleichgewichts	0,99	0,82	1,21	0,939
B96	Sonstige näher bezeichnete Bakterien als Ursache von Krankheiten, die in anderen Kapiteln klassifiziert sind	0,99	0,75	1,30	0,936
T16	Fremdkörper im Ohr	0,99	0,64	1,52	0,959
I88	Unspezifische Lymphadenitis	0,99	0,58	1,68	0,965
N94	Schmerz und andere Zustände im Zusammenhang mit den weiblichen Genitalorganen und dem Menstruationszyklus	0,98	0,81	1,20	0,859
N99	Krankheiten des Urogenitalsystems nach medizinischen Maßnahmen, anderenorts nicht klassifiziert	0,98	0,55	1,74	0,948
J40	Bronchitis, nicht als akut oder chronisch bezeichnet	0,98	0,81	1,18	0,831
R09	Sonstige Symptome, die das Kreislaufsystem und das Atmungssystem betreffen	0,98	0,51	1,88	0,950
Z99	Abhängigkeit (langzeitig) von unterstützenden Apparaten, medizinischen Geräten oder Hilfsmitteln, anderenorts nicht klassifiziert	0,98	0,68	1,40	0,893
T81	Komplikationen bei Eingriffen, anderenorts nicht klassifiziert	0,98	0,73	1,30	0,866
H17	Hornhautnarben und -trübungen	0,98	0,63	1,50	0,909
K26	Ulcus duodeni	0,97	0,56	1,69	0,914
J41	Einfache und schleimig-eitrige chronische Bronchitis	0,97	0,64	1,46	0,878

R93	Abnorme Befunde bei der bildgebenden Diagnostik sonstiger Körperstrukturen	0,97	0,70	1,33	0,836
K50	Crohn-Krankheit [Enteritis regionalis] [Morbus Crohn]	0,96	0,61	1,53	0,877
D35	Gutartige Neubildung sonstiger und nicht näher bezeichneter endokriner Drüsen	0,96	0,58	1,59	0,882
K74	Fibrose und Zirrhose der Leber	0,96	0,60	1,52	0,852
N13	Obstruktive Uropathie und Refluxuropathie	0,95	0,65	1,41	0,817
J43	Emphysem	0,95	0,70	1,30	0,765
T83	Komplikationen durch Prothesen, Implantate oder Transplantate im Urogenitaltrakt	0,95	0,54	1,69	0,866
J18	Pneumonie, Erreger nicht näher bezeichnet	0,95	0,70	1,28	0,739
L89	Dekubitalgeschwür und Druckzone	0,95	0,65	1,37	0,770
I64	Schlaganfall, nicht als Blutung oder Infarkt bezeichnet	0,95	0,62	1,43	0,792
E86	Volumenmangel	0,94	0,71	1,25	0,689
I69	Folgen einer zerebrovaskulären Krankheit	0,94	0,75	1,18	0,608
G40	Epilepsie	0,94	0,71	1,26	0,681
S01	Offene Wunde des Kopfes	0,94	0,64	1,39	0,754
J22	Akute Infektion der unteren Atemwege, nicht näher bezeichnet	0,94	0,60	1,47	0,782
K11	Krankheiten der Speicheldrüsen	0,94	0,52	1,70	0,832
I97	Kreislaufkomplikationen nach medizinischen Maßnahmen, anderenorts nicht klassifiziert	0,94	0,54	1,62	0,815
A09	Sonstige und nicht näher bezeichnete Gastroenteritis und Kolitis infektiösen und nicht näher bezeichneten Ursprungs	0,94	0,81	1,07	0,342
S93	Luxation, Verstauchung und Zerrung der Gelenke und Bänder in Höhe des oberen Sprunggelenkes und des Fußes	0,93	0,71	1,22	0,612
A08	Virusbedingte und sonstige näher bezeichnete Darminfektionen	0,93	0,60	1,43	0,743
G81	Hemiparese und Hemiplegie	0,92	0,66	1,28	0,616
R32	Nicht näher bezeichnete Harninkontinenz	0,92	0,76	1,11	0,375
T01	Offene Wunden mit Beteiligung mehrerer Körperregionen	0,91	0,55	1,50	0,707

I12	Hypertensive Nierenkrankheit	0,90	0,57	1,43	0,654
J91	Pleuraerguss bei anderenorts klassifizierten Krankheiten	0,90	0,59	1,38	0,625
D63	Anämie bei chronischen, anderenorts klassifizierten Krankheiten	0,90	0,53	1,50	0,674
C79	Sekundäre bösartige Neubildung an sonstigen und nicht näher bezeichneten Lokalisationen	0,89	0,56	1,43	0,635
S32	Fraktur der Lendenwirbelsäule und des Beckens	0,89	0,58	1,36	0,583
F10	Psychische und Verhaltensstörungen durch Alkohol	0,89	0,69	1,14	0,342
S61	Offene Wunde des Handgelenkes und der Hand	0,88	0,57	1,37	0,571
J44	Sonstige chronische obstruktive Lungenkrankheit	0,87	0,75	1,02	0,084
J15	Pneumonie durch Bakterien, anderenorts nicht klassifiziert	0,87	0,48	1,58	0,656
Z02	Untersuchung und Konsultation aus administrativen Gründen	0,87	0,51	1,48	0,607
F31	Bipolare affektive Störung	0,87	0,50	1,51	0,618
R15	Stuhlinkontinenz	0,86	0,61	1,22	0,410
J96	Respiratorische Insuffizienz, anderenorts nicht klassifiziert	0,86	0,66	1,12	0,259
L97	Ulcus cruris, anderenorts nicht klassifiziert	0,85	0,50	1,44	0,535
F17	Psychische und Verhaltensstörungen durch Tabak	0,84	0,74	0,96	0,011
C78	Sekundäre bösartige Neubildung der Atmungs- und Verdauungsorgane	0,84	0,51	1,38	0,485
Z74	Probleme mit Bezug auf Pflegebedürftigkeit	0,83	0,66	1,05	0,122
Z22	Keimträger von Infektionskrankheiten	0,83	0,56	1,23	0,351
F01	Vaskuläre Demenz	0,83	0,52	1,33	0,437
S51	Offene Wunde des Unterarmes	0,83	0,43	1,58	0,562
R47	Sprech- und Sprachstörungen, anderenorts nicht klassifiziert	0,82	0,57	1,19	0,307
I27	Sonstige pulmonale Herzkrankheiten	0,82	0,55	1,23	0,346
G30	Alzheimer-Krankheit	0,82	0,53	1,28	0,390
G82	Paraparese und Paraplegie, Tetraparese und Tetraplegie	0,82	0,46	1,44	0,488
G35	Multiple Sklerose [Encephalomyelitis disseminata]	0,82	0,50	1,33	0,412

S72	Fraktur des Femurs	0,82	0,47	1,41	0,467
R40	Somnolenz, Sopor und Koma	0,81	0,43	1,56	0,535
I20	Angina pectoris	0,81	0,60	1,09	0,173
K08	Sonstige Krankheiten der Zähne und des Zahnhalteapparates	0,81	0,42	1,54	0,515
F00	Demenz bei Alzheimer-Krankheit	0,80	0,51	1,24	0,315
U69	Sonstige sekundäre Schlüsselnummern für besondere Zwecke	0,79	0,55	1,13	0,193
J38	Krankheiten der Stimmlippen und des Kehlkopfes, anderenorts nicht klassifiziert	0,79	0,45	1,39	0,406
Z50	Rehabilitationsmaßnahmen	0,78	0,45	1,35	0,374
J37	Chronische Laryngitis und Laryngotracheitis	0,75	0,46	1,24	0,261
N17	Akutes Nierenversagen	0,75	0,50	1,13	0,167
D62	Akute Blutungsanämie	0,75	0,47	1,20	0,232
I63	Hirninfarkt	0,75	0,53	1,05	0,096
T89	Sonstige näher bezeichnete Komplikationen eines Traumas	0,74	0,47	1,17	0,199
C67	Bösartige Neubildung der Harnblase	0,72	0,42	1,25	0,245
G83	Sonstige Lähmungssyndrome	0,72	0,42	1,21	0,216
K25	Ulcus ventriculi	0,70	0,40	1,24	0,224
G20	Primäres Parkinson-Syndrom	0,70	0,42	1,17	0,171
S70	Oberflächliche Verletzung der Hüfte und des Oberschenkels	0,69	0,36	1,31	0,251
C34	Bösartige Neubildung der Bronchien und der Lunge	0,67	0,35	1,28	0,226
Z93	Vorhandensein einer künstlichen Körperöffnung	0,66	0,37	1,19	0,169
F07	Persönlichkeits- und Verhaltensstörung aufgrund einer Krankheit, Schädigung oder Funktionsstörung des Gehirns	0,66	0,35	1,25	0,203
G51	Krankheiten des N. facialis [VII. Hirnnerv]	0,64	0,34	1,17	0,146
F03	Nicht näher bezeichnete Demenz	0,50	0,35	0,73	< 0,001

Online-Tabelle 8 – Behandlungsprävalenz von Personen mit Vitiligo in allen analysierten Subgruppen

	≥ 12 Jahre			≥ 18 Jahre		
**Populationsgruppe**	**Anzahl der Personen, DADB**	**Anteil der Personen in der Prävalenzkohorte, %**	**Anteil der prävalenten Personen in der Therapiekohorte, %**	**Anzahl der Personen, DADB**	**Anteil der Personen in der Prävalenzkohorte, %**	**Anteil der prävalenten Personen in der Therapiekohorte, %**
**Prävalenzkohorte**						
**Alle Personen mit Vitiligo**	3.697	100,0	-	3.604	100,0	-
Behandelt durch beliebige Ärzt*innen	816	22,1	-	796	22,1	-
– topische Therapeutika[1]	622	16,8	-	602	16,7	-
– systemische Glukokortikosteroide[1]	241	6,5	-	241	6,7	-
– UV-Therapie[1]	28	0,8	-	28	0,8	-
– systemische Glukokortikosteroide[1] oder UV-Therapie[1]	268	7,2	-	268	7,4	-
Behandelt durch spezialisierte Fachärzt*innen	569	15,4	-	554	15,4	-
– topische Therapeutika[1]	484	13,1	-	469	13,0	-
– systemische Glukokortikosteroide[1]	96	2,6	-	96	2,7	-
– UV-Therapie[1]	28	0,8	-	28	0,8	-
– systemische Glukokortikosteroide[1] oder UV-Therapie[1]	124	3,4	-	124	3,4	-
**Therapiekohorte**						
**Personen mit Vitiligo ohne Ausschlussdiagnosen**	1.310	35,4	100,0	1.259	34,9	100,0
Behandelt durch beliebige Ärzt*innen	157	-	12,0	148	-	11,8
– topische Therapeutika[1]	121	-	9,2	112	-	8,9
– systemische Glukokortikosteroide[1]	43	-	3,3	43	-	3,4
– UV-Therapie[1]	< 10	-	-	< 10	-	-
– systemische Glukokortikosteroide[1] oder UV-Therapie[1]	46	-	3,5	46	-	3,7
Behandelt durch spezialisierte Fachärzt*innen	107	-	8,2	100	-	7,9

– topische Therapeutika[1]	94	-	7,2	87	-	6,9
– systemische Glukokortikosteroide[1]	15	-	1,1	15	-	1,2
– UV-Therapie[1]	< 10	-	-	< 10	-	-
– systemische Glukokortikosteroide[1] oder UV-Therapie[1]	18	-	1,4	18	-	1,4

[1] Personen, die Behandlungen aus mehr als einer Therapieform erhielten, wurden mehrfach erfasst. **DADB**: Deutsche Analysedatenbank für Evaluation und Versorgungsforschung

Online-Tabelle 9 – Übersicht der eingesetzten Therapien bei Personen mit Vitiligo in allen analysierten Subgruppen

Alle behandelten Personen mit Vitiligo					
		Behandelt durch beliebige Ärzt*innen		Behandelt durch spezialisierte Fachärzt*innen	
Therapieform	Wirkstoffkategorie	Anzahl der Personen, DADB	Anteil der behandelten Personen, %	Anzahl der Personen, DADB	Anteil der behandelten Personen, %
**≥ 12 Jahre**					
Topisch Medikamentös[1]	Ruxolitinib und/oder Glukokortikosteroide und/oder Calcineurininhibitoren	622	76,2	484	85,1
	Glukokortikosteroide und/oder Calcineurininhibitoren	561	68,8	431	75,7
	Nur Glukokortikosteroide	399	48,9	298	52,4
	Nur Calcineurininhibitoren	85	10,4	77	13,5
	Nur Ruxolitinib	61	7,5	53	9,3
Systemisch Medikamentös[1]	Systemische Glukokortikosteroide	241	29,5	96	16,9
UV-Therapie[1]	UV-Therapie gesamt	28	3,4	28	4,9
	UV- und topische Therapeutika	14	1,7	14	2,5
	UV-Therapie und systemische Glukokortikosteroide	< 10	-	< 10	-
**≥ 18 Jahre**					
Topisch Medikamentös[1]	Ruxolitinib und/oder Glukokortikosteroide und/oder Calcineurininhibitoren	602	75,6	469	84,7
	Glukokortikosteroide und/oder Calcineurininhibitoren	544	68,3	419	75,6
	Nur Glukokortikosteroide	394	49,5	293	52,9
	Calcineurininhibitoren	79	9,9	72	13,0
	Nur Ruxolitinib	58	7,3	50	9,0
Systemisch Medikamentös[1]	Systemische Glukokortikosteroide	241	30,3	96	17,3

UV-Therapie[1]	UV-Therapie gesamt	28	3,5	28	5,1
	UV- und topische Therapie	14	1,8	14	2,5
	UV-Therapie und systemische Glukokortikosteroide	< 10	-	< 10	-
**Behandelte Personen mit Vitiligo ohne Ausschlussdiagnosen**					
		**Behandelt durch beliebige Ärzt*innen**		**Behandelt durch spezialisierte Fachärzt*innen**	
**Therapieform**	**Wirkstoffkategorie**	**Anzahl der Personen, DADB**	**Anteil der behandelten Personen, %**	**Anzahl der Personen, DADB**	**Anteil der behandelten Personen, %**
**≥ 12 Jahre**					
Topisch Medikamentös[1]	Ruxolitinib und/oder Glukokortikosteroide und/oder Calcineurininhibitoren	121	77,1	94	87,9
	Glukokortikosteroide und/oder Calcineurininhibitoren	97	61,8	73	68,2
	Nur Glukokortikosteroide	53	33,8	38	35,5
	Nur Calcineurininhibitoren	21	13,4	20	18,7
	Nur Ruxolitinib	24	15,3	21	19,6
Systemisch Medikamentös[1]	Systemische Glukokortikosteroide	43	27,4	15	14,0
UV-Therapie[1]	UV-Therapie gesamt	< 10	-	< 10	-
	UV- und topische Therapie	0	-	0	-
	UV-Therapie und systemische Glukokortikosteroide	0	-	0	-
**≥ 18 Jahre**					
Topisch Medikamentös[1]	Ruxolitinib und/oder Glukokortikosteroide und/oder Calcineurininhibitoren	112	75,7	87	87,0
	Glukokortikosteroide und/oder Calcineurininhibitoren	90	60,8	68	68,0
	Nur Glukokortikosteroide	50	33,8	35	35,0
	Nur Calcineurininhibitoren	19	12,8	18	18,0

	Nur Ruxolitinib	22	14,9	19	19,0
Systemisch medikamentös[1]	Systemische Glukokortikosteroide	43	29,1	15	15,0
UV-Therapie[1]	UV-Therapie gesamt	< 10	-	< 10	-
	UV- und topische Therapie	0	-	0	-
	UV-Therapie und systemische Glukokortikosteroide	0	-	0	-

[1] Personen, die Behandlungen aus mehr als einer Therapieform erhielten, wurden mehrfach erfasst.
